# Oleanolic Acid Lactones as Effective Agents in the Combat with Cancers—Cytotoxic and Antioxidant Activity, SAR Analysis, Molecular Docking and ADMETox Profile

**DOI:** 10.3390/ijms26094099

**Published:** 2025-04-25

**Authors:** Barbara Bednarczyk-Cwynar, Andrzej Günther, Piotr Ruszkowski, Szymon Sip, Przemysław Zalewski

**Affiliations:** 1 Department of Organic Chemistry, Faculty of Pharmacy, Poznan University of Medical Sciences, Collegium Pharmaceuticum 2 (CP.2), Rokietnicka Str. 3, 60-806 Poznan, Poland; andrzej.gunther@me.pl; 2 Center of Innovative Pharmaceutical Technology (CITF), Rokietnicka Str. 3, 60-806 Poznan, Poland; 3 Department of Pharmacology, Faculty of Pharmacy, Poznan University of Medical Sciences, Collegium Pharmaceuticum 1 (CP.1), Rokietnicka Str. 3, 60-806 Poznan, Poland; pruszkowski@gmail.com; 4 Department of Pharmacognosy and Biomaterials, Faculty of Pharmacy, Poznan University of Medical Sciences, Collegium Pharmaceuticum 1 (CP.1), Rokietnicka Str. 3, 60-806 Poznan, Poland; szymonsip@ump.edu.pl (S.S.); pzalewski@ump.edu.pl (P.Z.)

**Keywords:** natural products, natural products’ modifications, triterpenes, oleanolic acid, triterpene lactones, oleanolic acid lactones, SAR, cytotoxic activity, cytotoxic activity of triterpenes, molecular docking, antioxidant activity, antioxidant activity of triterpenes, ADMETox

## Abstract

Oleanolic acid derivatives, specifically lactones (**2**–**8**) and bromolactones (**9**–**14**), were synthesised and evaluated for their cytotoxic, antioxidant, and pharmacokinetic profiles. The compounds were characterised using molecular docking simulations targeting the 1M17 protein, representing the EGFR tyrosine kinase domain. Compound **6** emerged as the most promising candidate, demonstrating strong interactions with residues critical for EGFR activity, such as LYS 721 and ASP 831. Biological assays revealed that compounds **6**, **2**, and **10** exhibited IC_50_ values across various cancer cell lines in the micromolar range, with a favourable Selectivity Index. Antioxidant activity assays (CUPRAC and DPPH) highlighted compound **7** as the most substantial electron donor and compound **10** as the most influential radical scavenger. ADMETox analysis confirmed the favourable pharmacokinetic and safety profiles of the derivatives. These findings underscore the potential of the selected oleanolic acid derivatives as drug candidates for targeted cancer therapies, offering cytotoxic and antioxidant benefits. Despite their promising cytotoxic and antioxidant activities, translating oleanolic acid derivatives to clinical applications remains challenging due to their bioavailability and metabolic stability. Our findings highlight compound 6 as a leading candidate with enhanced activity, providing a foundation for further optimising and developing EGFR-targeted anticancer therapies.

## 1. Introduction

“Cancer” refers to a considerable group of diseases characterised by uncontrolled cell proliferation and growth attacking various organs. According to estimates from the World Health Organization (WHO) in 2019, different types of cancer were the first or second leading cause of death before the age of 70 years in most countries around the world [1]. It is expected that in 2040, there will be nearly 30 million cases of cancer [2]. Thus, searching for new chemotherapeutics is one of scientists’ most crucial research trends worldwide.

Notably, great hopes are associated with substances of natural origin. The advantages of these substances include the high availability of natural sources of these substances, their wide variety, and their generally better bioavailability than synthetic substances. Additionally, compounds of natural origin can be subjected to planned chemical modifications, producing new semisynthetic derivatives. They often exhibit a much higher desired pharmacological activity than the parent substance [3].

Natural products often have complex, three-dimensional structures resulting from millions of years of biological evolution. They contain chiral centres, heteroatoms, and a variety of functional groups that facilitate specific interactions with biological targets. They have a natural “drug chemical space” compatible with the active sites of enzymes and proteins. Natural products also offer higher biocompatibility than synthetic compounds, which minimizes toxicity and increases therapeutic effectiveness. In turn, synthetic compounds can be designed to have a more straightforward structure. Often, this structure is optimised for pharmacokinetics (e.g., bioavailability and stability) and specificity.

A combination of two groups—natural and synthetic compounds—are chemically modified compounds of natural origin. Appropriate design and planning of chemical modifications of compounds of natural origin allows for a more straightforward introduction of these modifications to improve physicochemical properties and enables de novo design of compounds based on structural data about target proteins. The structures of hemisynthetic derivatives can be designed to target specific enzymes or proteins based on structural computer data (e.g., molecular docking). The optimization of chemical modifications can lead to improved ADMETox parameters compared to their natural parent compounds [4,5,6].

Terpenoids (also known as isoprenoids) are a vast group of compounds of natural origin, numbering over 40,000 different species [7], which are particularly promising in the context of chemotherapy to treat cancer. The most significant and crucial isoprenoid subgroup is triterpenes, divided into oleananes, lupanes, and ursanes, and others [8].

**Figure 1:**
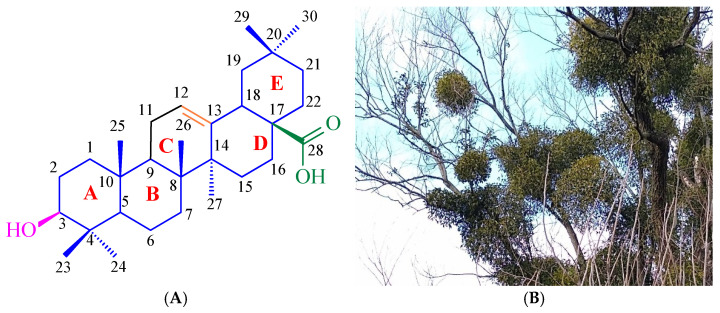
(**A**) Structure of oleanolic acid. (**B**) One source of oleanolic acid is the mistletoe (*Viscum album* L.) herb. Photo by B. Bednarczyk-Cwynar.

A representative of the triterpenes from the oleanane group is oleanolic acid (**1**, Figure 1A). This triterpene is present in over 1600 edible, medicinal, decorative, and wild plants [9]. One of the sources of oleanolic acid (**1**) is common mistletoe (*Viscum album* L., Figure 1B), from which the triterpene mentioned above (**1**) was isolated in the past at our university [10]. Another rich source of oleanolic acid (**1**) is olive leaves (*Olea europea*). Oleanolic acid (**1**) can be obtained from the above two plants with a yield of 1.7–3.0% by weight using standard extraction methods [11,12]. Modern techniques, such as ultrasonic-assisted extraction (UAE), can increase the efficiency of the process by up to 6% [13]. Oleanolic acid (**1**) is now available as a commercial product.

The growing interest in oleanolic acid (**1**) has resulted in numerous scientific publications regarding both chemical transformations and pharmacological tests. So far, several valuable biological properties of oleanolic acid (**1**) have been proven, e.g., anti-inflammatory [14], neuroprotective [15], antidepressant [16], hepatoprotective [17,18], antidiabetic [18], antiosteoporotic [19], antimicrobial [20], antiallergic [21], antiasthmatic [22], hypotensive [23], antihyperlipidemic [24], and others. Moreover, in vitro studies have demonstrated the cytotoxic activity of oleanolic acid (**1**) against various cancer cell lines, such as HepG2 [25], HeLa [26], B16F10 [27], HCT116 [28], L1210, K562 and HL-60 [29], HONE-1, KB, TH29 [30], and many others.

A factor that plays a key role in the pathogenesis of numerous diseases, including cancer, is oxidative stress. This is a state of a disturbed balance between the production of free radicals and the body’s ability to neutralise them via antioxidant systems. Free radicals play essential functions in numerous life processes in a healthy body, such as breathing and some cell-mediated immune functions [31]. Free radicals unused by the body can react with many biomolecules, such as DNA [32], lipids [33], and proteins [34], initiating the peroxidation of membrane lipids, leading to the accumulation of lipid peroxides and the damage of DNA and proteins, and finally resulting in disease conditions. Free radicals play a significant role in the initiation and progression of cancer [35].

Research published by Wang et al. [36] showed that oleanolic acid (**1**) is not only a free radical scavenger acting through direct chemical reactions as it also has an indirect antioxidant effect. The indirect antioxidant effect of the discussed triterpene includes the inhibition of lipid peroxidation and stimulating cellular antioxidant defences.

The antioxidant and free radical scavenging activity of triterpenes is an exciting and promising research direction, so it is the research topic of many scientists, including Ling [37], Günther [38], and others.

One of the most advantageous types of chemical modification of oleanolic acid (**1**) into a product with the highest possible level of cytotoxic activity is to add to the C-12 atom of the oleanane molecule a substituent connected to the parent skeleton with a double bond, i.e., there should be a carbonyl group at the C-12 position, or hydroxyimine (oxime system) [39]. Considering the above observations, it was decided to obtain hydroxyimine derivatives in which, however, the C-17 carboxyl group will be modified differently than before [39], i.e., it will be involved in forming the lactone system. For comparison, it was also decided to obtain other lactone derivatives, namely those in which a bromine atom will be attached to the C-12 position, preventing further chemical transformations at this position.

The obtained results indicate that carrying out certain chemical modifications of the oleanolic acid (**1**) molecule leads to obtaining effective anticancer agents (IC_50_ below 5 micromoles) with a favourable ADMETox profile which may become drug candidates in the future.

## 2. Results

### 2.1. Synthesis of Oleanolic Acid Lactones *(**2**–**8**)* and Bromolactones *(**9**–**14**)*

**Figure 2:**
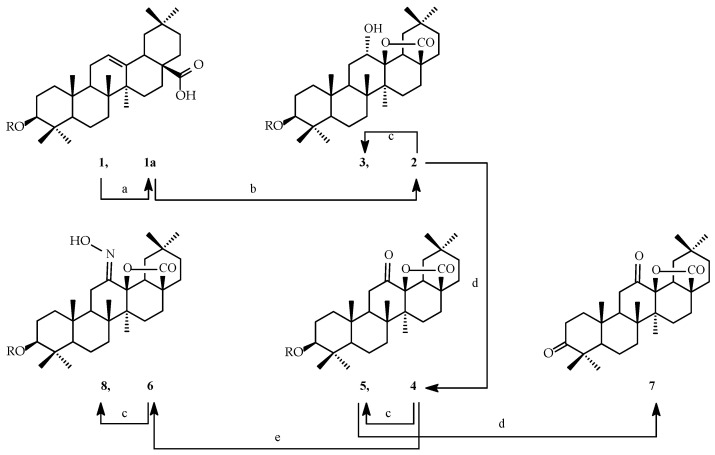
Synthesis of cytotoxic agents **2**–**8**. Legend: **1a**, **2**, **4**, **6**: **R** = CH_3_CO; **1**, **3**, **5**, **8**: **R** = H. Reagents and conditions: a = (CH_3_CO)_2_O, reflux; b = CHCl_3_, *m*-CPBA, r.t.; c = EtOH, KOH, reflux; d = acetone, Jones’ reagent, r.t.; e = EtOH, NH_2_OH × HCl, CH_3_COONa, reflux.

**Figure 3:**
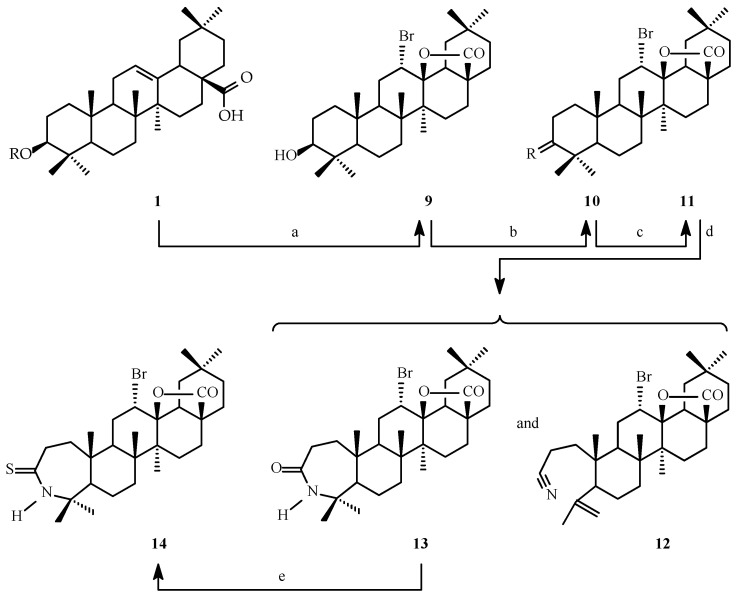
Synthesis of bromolactones **9**–**14**. Legend: **10**: R = O, **11**: R = NOH. Reagents and conditions: a = CH_3_COOH, Br_2_, r.t.; b = acetone, Jones’ reagent, r.t.; c = EtOH, NH_2_OH × HCl, CH_3_COONa, reflux; d = pyridine, POCl_3_, r.t.; e = benzene, Lawesson’s reagent, reflux.

Oleanolic acid of natural origin underwent a series of chemical transformations, resulting in derivatives with lactone or bromolactone systems. The performed chemical modifications are presented in Figure 2 and Figure 3.

### 2.2. SAR Analysis of Oleanolic Acid Lactones *(**2**–**8**)* and Bromolactones *(**9**–**14**)*

The highest results of SAR (Structure–Activity Relationship) analysis (P_a_ ≥ 0.700) for oleanolic acid (**1**), lactones **2**–**8** (Figure 2), and bromolactones **9**–**14** (Figure 3), based on predicted activities, are given in Table 1 and Table 2.

### 2.3. MTT Assay of Oleanolic Acid Lactones *(**2**–**8**)* and Bromolactones *(**9**–**14**)*

#### 2.3.1. Cytotoxic Properties

The cytotoxic activity of oleanolic acid (**1**), lactones **2**–**8** (Figure 2), and bromolactones **9**–**14** (Figure 3) was tested with the MTT assay for the following cell lines: A-549 (*human lung carcinoma*), CHO (*Chinese hamster ovary*), PC-3 (*human prostate carcinoma*), and SKOV-3 (*human ovarian carcinoma*) and compared to standard cell line HDFs (*human dermal fibroblasts*). The results are presented in Table 3.

#### 2.3.2. Selectivity Index

The Selectivity Index values for oleanolic acid (**1**), lactones **2**–**8** (Figure 2), and bromolactones **9**–**14** (Figure 3) determined in the MTT assay are given in Table 4.

#### 2.3.3. Apoptotic Index

The Apoptotic Index values for oleanolic acid (**1**), the most active lactones (compounds **2** and **6**, Figure 2), and bromolactones (compounds **10** and **12**, Figure 3) determined in the Elisa Plus assay are given in Table 5.

**Table 4:** Selectivity Index for oleanolic acid (**1**) and lactones **2**–**8** and bromolactones **9**–**14** determined in the MTT assay.

≥2.50	2.49–2.00	1.99–1.50	1.49–1.00	≤0.99

Comp. No.	Cell Line, SI			
	CHO	A-549	PC-3	SKOV-3
**1**	2.71	2.83	1.33	1.32
**2**	1.79	1.60	2.30	2.54
**3**	0.81	1.64	1.75	1.81
**4**	0.79	1.70	1.62	1.84
**5**	1.35	1.36	1.67	1.40
**6**	0.71	**3.08**	2.08	2.26
**7**	--- *	--- *	--- *	--- *
**8**	--- *	--- *	--- *	--- *
**9**	1.21	1.71	1.95	1.93
**10**	1.83	2.51	**3.02**	**3.24**
**11**	0.52	0.83	1.15	1.33
**12**	0.99	1.15	1.46	1.47
**13**	--- *	1.00	1.22	1.18
**14**	0.97	1.27	1.28	1.23

**Legend**: **SI** = Selectivity Index; **A-549** = *human lung carcinoma*; **CHO** = *Chinese hamster ovary*; **PC-3** = *human prostate carcinoma*; **SKOV-3** = *human ovarian carcinoma*; * = Selectivity Index was not calculated because the IC_50_ value exceeded 100 µM; yellow area = lactones; green area = bromolactones.

### 2.4. Molecular Docking of Oleanolic Acid Lactones *(**2**–**8**)* and Bromolactones *(**9**–**14**)*

#### 2.4.1. Detecting Cavities

The size of cavities, from the largest (C1) to the smallest (C5), are shown in Table 6.

#### 2.4.2. Molecular Docking Results

Results were obtained for 14 compounds, oleanolic acid (**1**) and its lactones **2**–**8** (Figure 2) and bromolactones **9**–**14** (Figure 3), and are presented in Figure 4, Figure 5, Figure 6, Figure 7, Figure 8, Figure 9 and Figure 10. Docking results for pockets C1 and C2, presented as Vina score values (kcal·mol^−1^), are given in Table 7.

**Table 7:** Docking results for pockets C1 and C2 presented as Vina score values (kcal·mol^−1^).

Pocket ID	1	2	3	4	5	6	7	8	9	10	11	12	13	14
**C1**	−8.0	−8.0	−7.8	−8.0	−8.5	−9.8	−7.9	−7.9	−9.7	−8.3	−9.0	−6.9	−8.6	−8.1
**C2**	−8.9	−9.4	−8.8	−9.2	−8.9	−10.3	−9.1	−9.2	−8.7	−9.7	−9.6	−8.1	−9.8	−9.9

**Figure 4:**
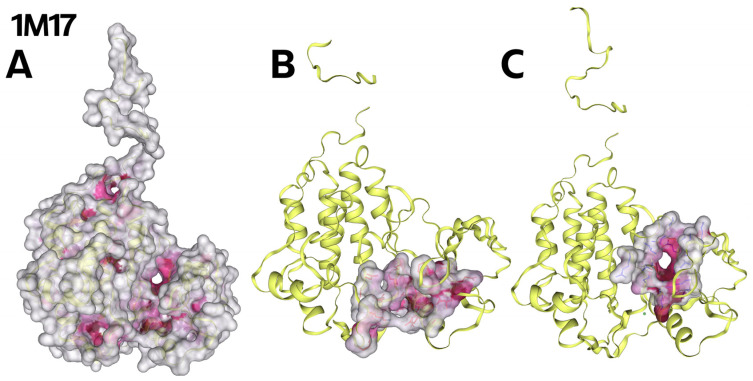
(**A**) Whole EGFR tyrosine kinase domain molecule (PDB ID 1M17); the dark pink colour indicates the cavity of the molecule. (**B**) View of the largest pocket, C1, with a volume of 991 Å^3^. (**C**) View of pocket number two, C2, with a volume of 665 Å^3^.

**Figure 5:**
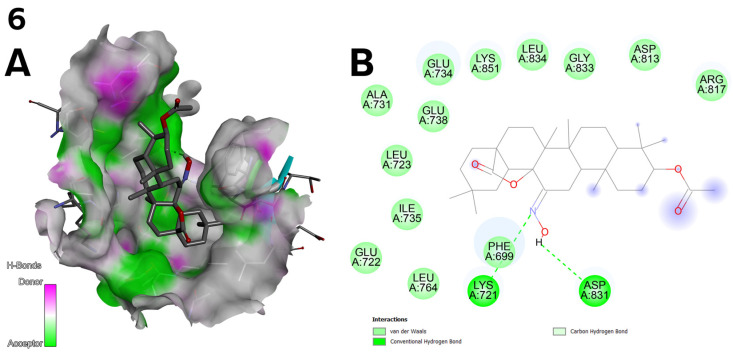
(**A**) Three-dimensional surface of structure of EGFR tyrosine kinase domain molecule (PDB ID 1M17) with ligand compound **6** with H-Bonds gradient map in C1 pocket. (**B**) Diagram in 2D with interactions.

**Figure 6:**
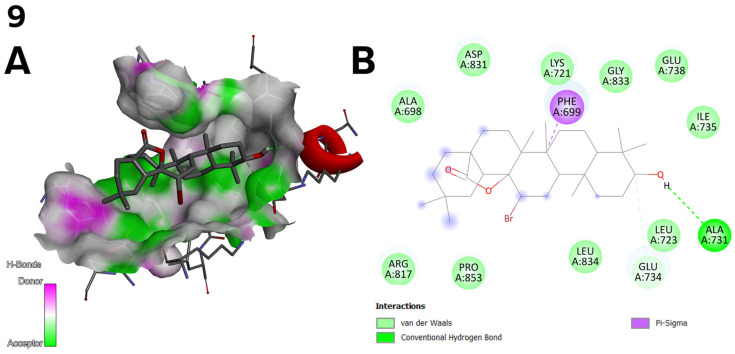
(**A**) Three-dimensional surface of structure of EGFR tyrosine kinase domain molecule (PDB ID 1M17) with ligand compound **9** with H-Bonds gradient map in C1 pocket. (**B**) Diagram in 2D with interactions.

**Figure 7:**
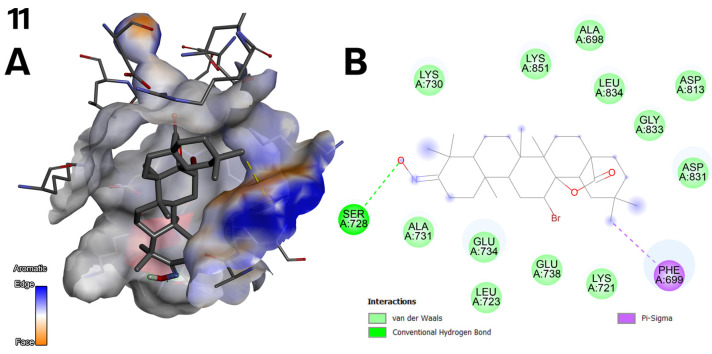
(**A**) Three-dimensional surface of structure of EGFR tyrosine kinase domain molecule (PDB ID 1M17) with ligand compound **11** with aromatic gradient map in C1 pocket. (**B**) Diagram in 2D with interactions.

**Figure 8:**
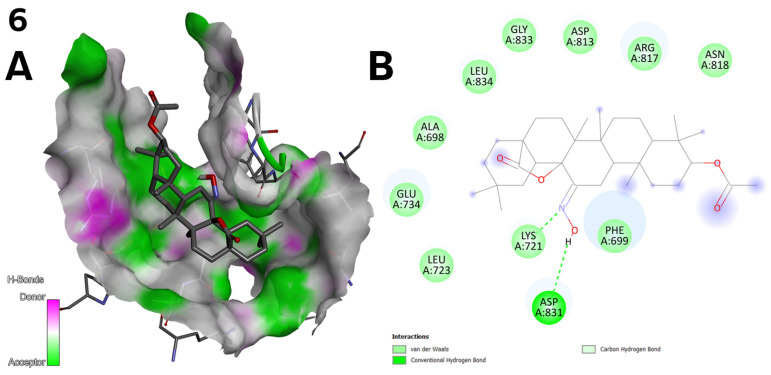
(**A**) Three-dimensional surface of structure of EGFR tyrosine kinase domain molecule (PDB ID 1M17) with ligand compound **6** with H-Bonds gradient map in C2 pocket. (**B**) Diagram in 2D with interactions.

**Figure 9:**
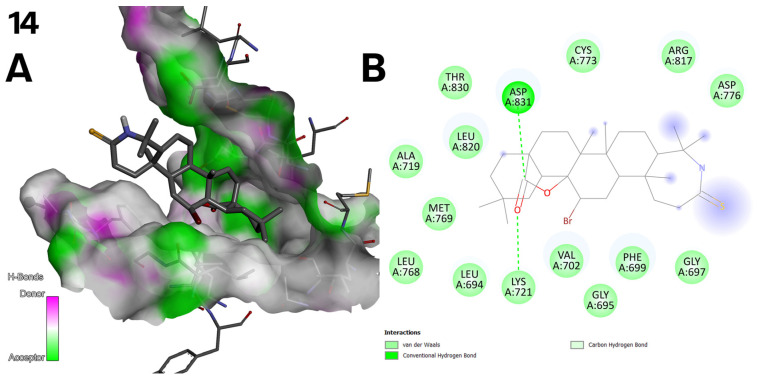
(**A**) Three-dimensional surface of structure of EGFR tyrosine kinase domain molecule (PDB ID 1M17) with ligand compound **14** with H-Bonds gradient map in C2 pocket. (**B**) Diagram in 2D with interactions.

**Figure 10:**
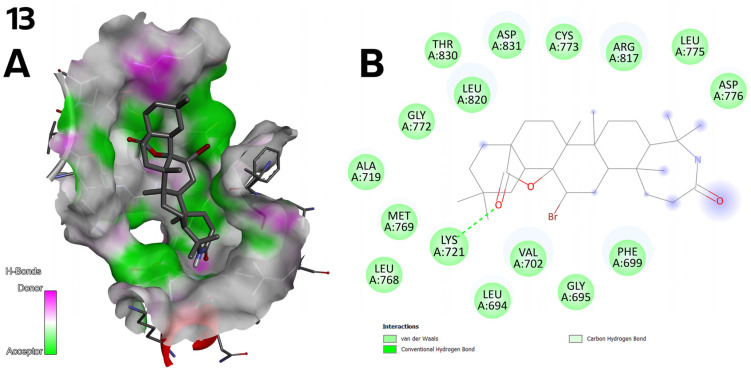
(**A**) Three-dimensional surface of structure of EGFR tyrosine kinase domain molecule (PDB ID 1M17) with ligand compound **13** with H-Bonds gradient map in C2 pocket. (**B**) Diagram in 2D with interactions.

### 2.5. Antioxidant Activity of Oleanolic Acid, Lactones *(**2**–**8**)*, and Bromolactones *(**9**–**14**)*

**Figure 11:**
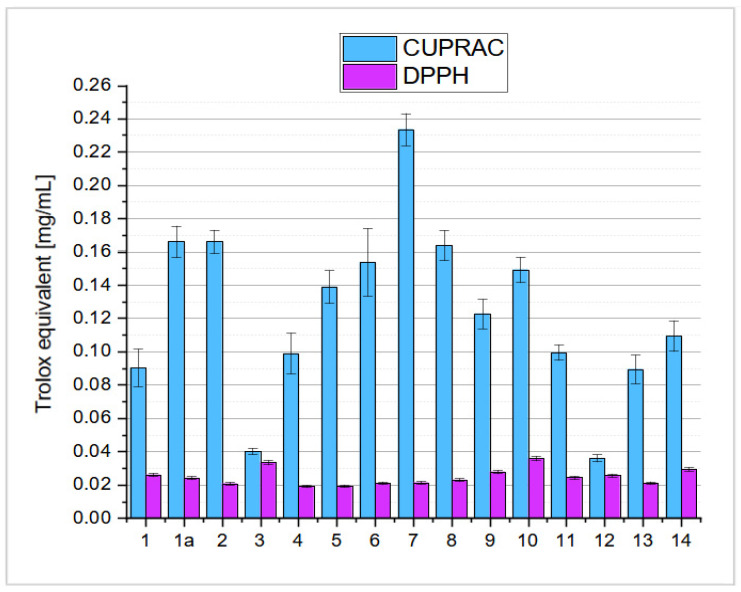
Antioxidant activity in CUPRAC and DPPH assays of lactones **2**–**8**, bromolactones **9**–**14**, and oleanolic acid (**1**), expressed as Trolox equivalent.

The results of the antioxidant activity of oleanolic acid (**1**) and its lactones **2**–**8** (Figure 2) and bromolactones **9**–**14** (Figure 3), evaluated with CUPRAC and DPPH assays [42], are given in Figure 11. The results are presented as % inhibition of the copper(II) ions and Trolox equivalent (blue bars) calculated from the standard curve (Supplementary Materials, File S1, Figure S1A) and as % inhibition of the DPPH radical and Trolox equivalent (pink bars), calculated from the standard curve (Supplementary Materials, File S1, Figure S1B).

### 2.6. ADMETox Analysis of Oleanolic Acid Lactones *(**2**–**8**)* and Bromolactones *(**9**–**14**)*

The detailed results of computer calculations regarding ADMETox parameters obtained for lactones (**2**–**8**, Figure 2) and bromolactones (**9**–**14**, Figure 3) are provided in the Supplementary Materials (File S2, Tables S1 and S2).

## 3. Discussion

### 3.1. Synthesis of Oleanolic Acid Lactones *(**2**–**8**)* and Bromolactones *(**9**–**14**)*

As a result of the chemical transformations carried out, 13 oleanolic acid derivatives were obtained, each having either (i) a lactone system within a triterpene skeleton or (ii) a bromolactone system within a triterpene skeleton. These transformations are presented in Figure 2 and Figure 3.

To obtain the first group of planned compounds (**2**–**8**), oleanolic acid (**1**) was reversibly acylated with acetic acid anhydride.

In oleanolic acid derivatives, the 28β→13β-olide system with a hydroxyl group at the C-12α position is formed under the action of ozone or KMnO_4_, e.g., [43,44,45]. According to our method in this publication, the same transformation was performed by treating 3β-acetoxyolean-12-en-28-oic acid (**1a**) with *m*-chloroperbenzoic acid (*m*-CPBA) in chloroform. The obtained 3β-acetoxy-12α-hydroxylactone (**2**, Figure 2) was quickly subjected to alkaline hydrolysis, forming 3β,12α-dihydroxylactone (**3**). In the molecule of derivative **2**, besides the planned 28β→13β-olide system, there was a hydroxyl group at the C-12 position. This C-12 hydroxyl group was oxidised to the C-12 ketone group, obtaining compound **4** (3-acetoxy-12-oxolactone). The melting points and spectral data of all three compounds (i.e., **2**, **3**, and **4**) were consistent with the relevant literature [43,46]. Derivative **4**, like **2**, readily underwent alkaline hydrolysis of the C-3 acetoxy moiety to the C-3 hydroxyl (**5**, Figure 2).

Reactions of converting the C-12 ketone group in the molecule of oleanolic acid derivatives into a 12-hydroxyimine (oxime) group are known from the literature, e.g., [39]. An analogous reaction was performed for derivative **4** (3-acetoxy-12-oxolactone) [46], and the obtained oxime **6** (Figure 2) was also subjected to alkaline hydrolysis (like compounds **2** and **4**). This reaction allowed to obtain the corresponding oxime with the C-3 hydroxyl group (compound **8**, Figure 2). In turn, 3-hydroxy-12-ketone **5** was oxidised with Jones’ reagent to obtain 3,12-diketone **7** (Figure 2), which was to be expected. The oxime **6** (3-acetoxy-12-hydroxyiminolactone) was subjected to Beckmann rearrangement according to the procedure known in the literature [46]. The obtained nitrile and lactam were characterised by very poor solubility in common organic solvents, including solvents used for biological research. For this reason, the above two compounds are not presented in Figure 2 nor in the Section 4 Materials and Methods.

To obtain the second group of the planned derivatives (**9**–**14**, Figure 3), oleanolic acid (**1**), dissolved in acetic acid, was treated with bromine. During this reaction, the C-17 carboxyl group in the parent triterpene molecule (**1**) was transformed into the 28β→13β-olide system, similarly as for derivative **2**. However, at the same time, one bromine atom was added to the double bond and finally derivative **9** (3-hydroxy-12-bromolactone, Figure 3) was obtained. Derivative **9** obtained according to our procedure was characterised by high purity.

The molecule of bromolactone **9** contained the C-3 hydroxyl group, which was oxidised to a ketone group (derivative **10**, Figure 3), and the resulted 3-oxo-12-bromolactone **10** was next transformed into the corresponding oxime (derivative **11**, Figure 3). The 3-oxime-12-bromolactone **11** was subjected to a Beckmann rearrangement reaction [39], which resulted in the production of two products with significantly different polarity: a slightly polar (compared to oleanolic acid, **1**) nitrile **12** and a highly polar lactam **13** (Figure 3). The last planned chemical transformation was the exchange of the oxygen atom in the lactam system of bromolactone **13** on the sulphur atom, resulting in the thiolactam-bromolactone **14** (Figure 3).

The spectral data of the obtained derivatives **1a**–**14** confirmed, beyond any doubt, the structures of all the derivatives.

### 3.2. SAR Analysis of Oleanolic Acid Lactones *(**2**–**8**)* and Bromolactones *(**9**–**14**)*

In silico predictions (e.g., PASS; Prediction of Activity Spectra for Substances) are not real experimental results, but they are a supporting tool that requires experimental verification. The PASS results only indicate potential biological activities and cannot be treated as definitive evidence of compound activity.

Computer simulations of the relationship between the structure of the substances and their pharmacological activity indicate that oleanolic acid (**1**) and its lactones (**2**–**8**; Figure 2) and bromolactones (**9**–**14**; Figure 3) may exhibit up to 25 types of biological activity in in vitro tests (Table 1 and Table 2). These activities are indicated by a high probability of occurring, more than 0.700. The most common directions of pharmacological activity are related to cytotoxic effects on cancer cells.

The SAR analysis showed that most of the obtained substances could be effective antineoplastic agents, apoptosis agonists, caspase-3 and caspase-8 stimulants, and TF, particularly TF NF kappa β (NF-κB), stimulants. Pro-apoptotic caspases, such as caspase-3 and caspase-8, are enzymes that degrade cancer cell proteins when activated by apoptosis signals. The caspase activation mechanism is a significant signalling pathway leading to apoptosis [47]. Nuclear-kappa B factor is a transcription factor that plays a key role in the expression of genes encoding proteins produced in response to pro-inflammatory stimuli and is involved in cell proliferation and apoptosis [48]. Based on the data obtained, we can assume that the cytotoxic activity of the tested triterpenes against cancer lines will be based on the mechanism of stimulating the action of the transcription factor NF kappa B.

From the data included in Table 1 and Table 2, it is easy to notice the impact of the presence or absence of various substituents at the C-3 and/or C-12 positions upon the five most probable directions of pharmacological activity of derivatives **2**–**14** (antineoplastic activity, apoptosis agonist activity, caspase-3- and caspase-8-stimulating activity, and TF NF kappa β-stimulating activity). Without any doubt, the transformation of the carboxyl group within the oleanolic acid molecule (**1**) into the lactone system, regardless of the type of substituent at the C-3 and C-12 positions (Figure 2), significantly increased the probability of antineoplastic activity (Table 1): P_a_ = 0.876 for oleanolic acid (**1**) and P_a_ from 0.928 to 0.951 for derivatives **2**–**8**, with the highest chance of neoplastic activity for 3-acetoxy-12-oxolactone **4** and 3,12-dioxolactone **7** (Figure 2). Therefore, it can be concluded from the above data that an important functional group influencing the antineoplastic activity is probably the carbonyl group at the C-12 position. Moreover, it is highly probable that the second of the analysed derivatives, i.e., 3,12-dioxolactone **7** (Figure 2), would be the most active apoptosis agonist among lactones **2**–**8** (Table 1; P_a_ = 0.921; for the mother compound, **1**, P_a_ = 0.901). At the same time, 3,12-dioxolactone **7** was less likely than the other lactones (compounds **2**–**6** and **8**, Figure 2) to be a caspase-3 stimulant (Table 1; P_a_ = 0.764). The most active caspase-3 stimulants would be 3-hydroxy-12-oxolactone **5** and 3-hydroxy-12-hydroxyiminolactone **8** (Figure 2), for which the probability of occurrence of the analysed activity exceeded 0.950 (Table 1). A common feature of the structure of the molecule of lactones **5** and **7** (Figure 2) is the presence of a hydroxyl group at the C-3 position. In turn, the transformation of the C-17 carboxyl group into the 28β→13β-olide system resulted in a lower probability of occurrence of caspase-8-stimulating activity, chemopreventive, hepatoprotective, and other activities compared to the probability of occurrence of these activities for the parent oleanolic acid (**1**).

The introduction of a bromine atom into the C-12 position of the oleanolic acid (**1**) molecule and, at the same time, the transformation of the C-17 carboxyl group into the 28β→13β-olide system (Figure 3) is a fascinating transformation from the point of view of chemistry. However, from the point of view of pharmacology, it is not always a favourable reaction. As Table 2 shows, only in cases of several types of pharmacological activities, the probability of occurrence of these activities for bromolactones **9**–**14** (Figure 3) exceeds 0.700. These activities are antineoplastic, apoptosis agonist, and transcription factor-stimulant activities. A moderate, beneficial effect of the C-3 ketone and hydroxyimine groups and the A-ring cleveaged nitrile system on the probability of occurrence of the above pharmacological activities was observed; only for 3-hydroxybromolactone **9** and 3-oxo-bromolactone **10** (Figure 3), the P_a_ value exceeded 0.900 for antineoplastic activity (Table 2).

The reason for the low chances of most activities predicted by the program is the presence of the bromine atom. Of course, the presence of a bromine atom in the chemical structure of a substance can influence its pharmacological activity positively or negatively, depending on the context. If the bromine atom reduces pharmacological activity, the reasons may be related to the chemical and physicochemical properties of bromine and its effect on the structure and properties of the molecule. The main reasons for reducing the level of pharmacological activity are as follows [49]:

- **Impact of bromine atom upon lipophilicity and solubility**: The bromine atom is relatively large and heavy and has moderate polarity. Its presence increases the lipophilicity of the molecule. Excessive lipophilicity may hinder the dissolution of the substance in water, which reduces its bioavailability and causes accumulation in lipid membranes, reducing accessibility to the site of action.
- **Changes in the shape and volume of the molecule**: The bromine atom has a large diameter, which may introduce steric difficulties in the molecule. These difficulties may disturb interactions with the receptor or enzyme (e.g., prevent the substance from adequately binding to the active site) and reduce the affinity of the substance for the biological target.
- **Impact on metabolic reactions**: The bromine atom may affect the metabolic stability of a substance because it may be susceptible to biotransformation reactions (e.g., dehalogenation reaction), leading to the formation of an inactive metabolite or may cause the molecule to become more susceptible to elimination from the body (e.g., by conjugation with glutathione or glucuronic acid).
- **Impact on hydrogen bonds**: The introduction of a bromine atom replaces functional groups that could participate in the formation of hydrogen bonds (e.g., hydroxyl or amino groups). Such a new structure may limit the substance’s ability to form interactions key to its biological activity.

### 3.3. MTT Assay of Oleanolic Acid Lactones *(**2**–**8**)* and Bromolactones *(**9**–**14**)*

#### 3.3.1. Cytotoxic Activity

Biological tests (MTT; 3-(4,5-dimethylthiazol-2-yl)-2,5-diphenyltetrazolium bromide) were performed for the mother compound—oleanolic acid (**1**)—and its 13 derivatives: 7 lactones (**2**–**8**; Figure 2) and 6 bromolactones (**9**–**14**; Figure 3). The results are presented as IC_50_ values (Table 3). The IC_50_ value represents the compound concentration required to inhibit a biological process, such as cell proliferation, by 50%. It is a standard measure of a compound’s potency in pharmacological studies.

The CHO line was the first cell line for which the MTT test was performed for the parent oleanolic acid and its 13 derivatives (Table 3).

The CHO (*Chinese hamster ovary*) cell line is widely used in biological research, including cytotoxicity studies, even though it is not cancerous. The choice of this cell line is due to several key advantages [50]:

1. **Genetic stability and ease of cultivation**: CHO cells are well stabilized and easy to culture in vitro. They are adapted to grow both in suspension and on surfaces, which facilitates their use in various experiments.
2. **Capacity for genetic modification**: CHO cells can be easily subjected to genetic modification, which allows them to be adapted to specific studies, such as assessing the cytotoxicity of drugs or studying the mechanisms of action of various substances.
3. **Non-cancerous**: Because CHO cells are non-cancerous, they are a good model for studies that aim to assess the effect of substances on healthy eukaryotic cells. Using a tumour line could complicate the interpretation of the results because such cells have disturbed regulatory mechanisms, which may lead to atypical responses to the tested substances.
4. **Mammalian representativeness**: CHO cells are of mammalian origin, making them more similar to human cells in structure, metabolism, and biochemical mechanisms. Therefore, they are a better model than bacterial or insect-origin cells.
5. **High sensitivity to toxins**: CHO cells are sensitive to many toxic compounds, making them a good model for cytotoxicity testing. They can be used to evaluate various substances, from drugs to environmental toxins.
6. **Wide acceptance in regulatory research**: The CHO cell line is accepted by many regulatory institutions, e.g., the FDA (Food and Drug Administration) and EMA (European Medicines Agency), as a model used in preclinical research. This acceptance is due to the well-established place of CHO cells in pharmacological and toxicological research.

The choice of CHO cells in cytotoxicity studies is due to their versatility, stability, and ability to reflect the response of healthy mammalian cells to various substances. These reasons make CHO cells an ideal model for basic and applied research, even though they are non-cancerous. In our tests on the CHO line, we have shown that **lactone derivatives 2 and 6 (**Figure 2), i.e., 3-acetoxy-12-hydroxylactone (**2**) and 3-acetoxy-12-hydroxyiminolactone (**6**), are compounds almost twice as active as the parent oleanolic acid (**1**) (Table 3). However, one of the bromolactones, or more precisely A-ring cleveaged nitrile 12-bromolactone **12** (Figure 3), is a comparably active compound as the parent oleanolic acid (**1**) (Table 3). The remaining oleanolic acid lactones and bromolactones **(**Figure 2 and Figure 3) showed moderate or even low activity in the MTT test on the non-cancer CHO line, with IC_50_ values ranging from 38 µM to over 100 µM (Table 3). Because the CHO line is relatively resistant to the action of many compounds, its use in research may help assess the potential non-specific toxicity of compounds **2**, **6**, and **12**.

The results obtained for the CHO line provide an excellent starting point for more advanced studies, i.e., in human cancer lines.

In the next step of our investigations, the MTT assay was conducted for oleanolic acid (**1**) and its seven lactones (**2**–**8**, Figure 2) and six bromolactones (**9**–**14**, Figure 3), with the application of three cancerous (A-549, PC-3, and SKOV-3) cell lines and one non-cancerous (HDFs) cell line. The results, expressed as half maximal inhibitory concentration (IC_50_), are presented in Table 3.

The IC_50_ value for the parent compound (**1**) was 8.79 μM for the A-549 cell line, 18.63 μM for the PC-3 cell line, and 18.81 μM for the SKOV-3 cell line (Table 3).

For the A-549 line, the IC_50_ value ranged from 1.27 μM (for 3-acetoxy-12-hydroxyiminolactone **6**) to >100 μM (for 3-hydroxy-12-hydroxyiminolactone **8**); for the PC-3 and the SKOV-3 cell lines, the IC_50_ values were 1.88 and 1.73 μM, respectively (both values for 3-acetoxy-12-hydroxylactone **2**), and in both cases the less active compounds were lactones **7** (3,12-dioxolactone) and **8** (for both compounds, the IC_50_ exceeded 100 μM) (Table 3).

Among the tested 28β→13β-olides **2**–**8** (Figure 2), with various moieties at the C-3 and the C-12 positions, the most active turned out to be compound **6**, containing an acetoxy function at the C-3 position and a hydroxyimine moiety at the C-12 position. This compound was highly active, at 1.27–1.73 μM for A-549 and CHO cells, respectively (Table 3). This derivative **6** was two to nine times more active than the parent oleanolic acid (**1**). According to the guidelines from the scientific literature [51], derivative **6** can be considered highly pharmacologically active, making it a good and promising drug candidate.

The transformation of 3-acetoxy-12-hydroxyiminolactone **6** into its derivative with an unsubstituted hydroxyl group at the C-3 position (compound **8**, Figure 2) led to a drastic reduction in the level of cytotoxic activity of the new derivative **8**, for which the IC_50_ value for all three cancer cell lines (A-549, PC-3, and SKOV-3) exceeded 100 μM (Table 3). Based on these results, it can be concluded that the acetoxy group at the C-3 position of the oleanane molecule, in addition to the C-12 hydroxyimine group, is an element necessary to obtain a high level of cytotoxic activity of the oleanolic acid derivative.

The second, only slightly less active cytotoxic agent was 3-acetoxy-12-hydroxylactone **2** (Figure 2), for which the IC_50_ value ranged from 2.96 μM (SKOV-3 cell line) to 4.68 μM (A-549 cell line) (Table 3). This compound **2** was 2–6 times more active than oleanolic acid (**1**), and, according to the scientific literature, it also can be considered a highly active drug candidate [51].

The transformation of the highly active 3-acetoxy-12-hydroxylactone **2** into its 3,12-dihydroxy derivative **3** (Figure 2) resulted in a significant reduction in the cytotoxic activity of the new derivative **3**. For this lactone **3**, the IC_50_ value for lines A-549, PC-3 and SKOV-3 ranged from about 21 to about 23 μM (Table 3), which means that this derivative **2** was 2–6 times less active than the parent oleanolic acid (**1**). The above relationship between the structure of the tested triterpene derivative and the level of its cytotoxic activity shows that while the presence of an acetoxy group in the C-3 position of the oleanane skeleton is very advantageous (as for compound **2**, Figure 2), replacing it with a hydroxyl group (as for compound **3**, Figure 2) is unfavourable.

Two further lactones, i.e., 3-acetoxy-12-oxolactone **4** and 3-hydroxy-12-oxolactone **5** (Figure 2), both with oxo substituent at the C-12 position but with different substituents at the C-3 position, were moderately active compounds. The IC_50_ limits for which the tested compound is moderately active are 10 μM < IC_50_ ≤ 30 μM [51]. Lactones **4** and **5** showed an IC_50_ value of about 24–38 μM (Table 3), which means they are less active than the parent oleanolic acid (**1**). However, lactones 4 and 5 were excellent substrates for further chemical transformations due to their structure, leading to more pharmacologically active derivatives.

The last lactone (3,12-dioxolactone **7**, Figure 2) was inactive against all three cancer cell lines used (A-549, PC-3, and SKOV-3; Table 3) and the IC_50_ value for this compound often exceeded 100 μM (Table 3). These results clearly show that the presence of two ketone groups in the molecule of bromolactone **7** is unfavourable and is responsible for the very low level of cytotoxic activity of triterpene derivative **7**.

Among the tested 28β→13β-olides with a bromine atom at the C-12 position (compounds **9**–**14**, Figure 3), the most active was compound **12**, i.e., the product of the Beckmann rearrangement reaction of the corresponding oxime (**11**), containing a cleveaged seven membered A-ring with a nitrogen atom (nitrile; Figure 3). For this compound, the IC_50_ value for both tested lines was below 10.0 μM, while the second, or rather main, product of the above rearrangement reaction, i.e., A-ring lactam (**13**), turned out to be the least active of the bromolactones **9**–**14** (Table 3).

The remaining bromolactones, i.e., compounds **9**, **10**, **13**, and **14** (Figure 3), were moderately or weakly active, and the IC_50_ value for these compounds ranged from about 11 to about 90 μM (Table 3). The most active of the mentioned bromolactones **9**, **10**, **13**, and **14** (Figure 3) was 3-oxobromolactone **10**, for which the IC_50_ values were approximately 12–15 μM. Interestingly, oxime with a bromolactone system, i.e., compound **11**, was moderately active (IC_50_ approximately 22–35 μM; Table 3).

Taking into account all the results obtained for the 13 triterpenes and cancer cell lines, it was observed that three derivatives of oleanolic acid deserve special attention, namely acetoxylactone with the C-12 hydroxyl group (**2**), bromolactone with an A-nitrile system (**12**), and the most active compound, acetoxylactone with a C-12 hydroxyimine group (**6**) (Figure 2 and Figure 3). The half maximal inhibitory concentration (IC_50_) value for the three derivatives mentioned was from 1 to 9 μM (Table 3), so these three compounds may become potent anticancer drug candidates. An additional advantage of these three substances, supporting their potential use as drug candidates, is the favourable parameters of ADMETox, as presented in the Supplementary Materials (File S2, Tables S1 and S2).

The above observations regarding the high level of cytotoxic activity of oxime **6** are consistent with the results of our previous studies [39]. These studies have shown that the most likely element of the structure of the triterpene molecule that has the most beneficial effect on the level of cytotoxic activity is the double bond between the carbon atom and the nitrogen atom, and such a system, present in the oxime molecule and its acyl analogues, should be in the position C-12.

As shown by the results in Table 3 (and also Figure 11), in order to obtain triterpene derivatives of the nature of lactones showing a high level of pharmacological activity, it is advantageous to design the structure of the new lactone derivative in such a way that a hydroxyl or hydroxyimino group is present at the C-12 position, with the hydroxyl group in the C-3 position of the oleanane molecule should be blocked with an acetyl residue. The hydroxyl (-OH) and hydroxyimine (=NOH) groups are beneficial for the biological activity of chemical compounds for several reasons related to their chemical and physicochemical properties and biological interactions.

The most important reasons why these two functional groups are the main element that positively influences the level of cytotoxic activity are as follows [52,53]:

1. **Participation in hydrogen bonds**: Hydrogen bonds are crucial for the ligand–receptor recognition and stabilization of biological complexes, translating into higher biological activity.
  ○ Hydroxyl groups (-OH) can act as hydrogen bond donors and acceptors. They facilitate the formation of specific interactions with the functional groups of target proteins (e.g., amino acid residues in the enzyme’s active site).
  ○ The hydroxyimine group (=NOH) can act as a hydrogen bond donor and the nitrogen atom is an acceptor. This structure allows an oxime to form stable complexes with proteins or nucleic acids.
2. **Stabilization of enzymatic reactions**: This property allows precise regulation of enzymatic functions.
  ○ Hydroxyl groups can stabilize the transient states of enzymes during catalysis and interact with metals in the active sites of enzymes, acting as coordinating ligands (e.g., in metalloproteins).
  ○ Hydroxyimine groups, thanks to the presence of nitrogen and oxygen atoms, can inhibit the activity of some enzymes by chelating metal ions (e.g., Zn^2+^ in enzymes of the metalloenzyme class).
3. **Impact on hydrophobic and hydrophilic interactions**: Hydroxyl and hydroxyimine groups facilitate the introduction of polar sites on the surface of molecules and may increase the selectivity of interactions with biological targets that have polar surfaces (e.g., active sites of enzymes).
4. **Capacity for chemical reactions**: Modifications may affect the biological activity or stability of the compound in the body. Hydroxyl and hydroxyimine groups can be the site of chemical reactions, e.g., phosphorylation, glycosylation or acetylation (important in biological processes), and the formation of covalent bonds with target proteins (e.g., irreversible enzyme inhibitors).
5. **Antioxidant properties**: The hydroxyl group can neutralize reactive oxygen species (ROS) or reactive nitrogen species (RNS) by donating hydrogen. The hydroxyimine group has a similar activity thanks to a hydroxyl group constituting its fragment.
6. **Increasing specificity of action**: Hydroxyl and hydroxyimine groups can improve the binding specificity of the ligand to the target protein, avoiding interactions with unrelated proteins.

A big surprise, however, is the high cytotoxic activity of nitrile **12** (Table 3). Our previous research showed that lactams are cytotoxically active compounds [54]. The structures of these lactams [54] clearly show that the C-17 carboxyl group is not involved in forming the 28β→13β-olide system in these compounds. In turn, the corresponding nitriles, formed concurrently during the Beckmann rearrangement reaction of the appropriate oximes, are generally inactive or weakly active [54]. It can be suspected that the surprisingly high level of cytotoxic activity towards to all cancer cell lines (A-549, PC-3, and SKOV-3, Table 3), but also in relation to non-cancerous cells (CHO and HDF; Table 3) is related to the simultaneous presence of two characteristic elements of the structure of derivative **12** (Figure 3), i.e., the nitrile group within the cleveaged A-ring and bromine atom attached at the C-12 position. This is only a hypothesis, and its confirmation (or denial) will be carried out in the future because it is planned to obtain several new derivatives in which various chemical transformations will be carried out. However, it can also be suspected that the simultaneous presence of a bromine atom and a nitrile group in the structure of a chemical compound may significantly improve its biological properties thanks to the synergy resulting from the unique features of each of these elements.

The bromine atom introduces local polarity to the molecule, which may increase its ability to interact with the active sites of enzymes or receptors. This atom often stabilises the structure of a chemical compound, which may delay its metabolism and extend its duration of action in the body. In the case of biological systems, the bromine atom can act as a “contact point”, increasing the bond strength to the biological target. In turn, the nitrile group is less polar and allows the formation of specific interactions (hydrogen bonds, dipole–dipole interactions) with target proteins. It improves the specificity of interactions with receptors, increasing biological activity. The high activity of bromolactone with the A-ring cleveaged nitrile system is probably due to the bromine atom’s synergistic action and the molecule’s nitrile group. The bromine atom and the nitrile group introduce two polar elements that may make it easier for the molecule to fit into the active sites of receptors or enzymes. The presence of two different interaction centres (bromine and nitrile) allows the formation of more stable ligand–receptor complexes. The presence of both groups may increase the specificity of interactions with biomolecules, minimizing off-target effects.

The influence of the presence of a bromine atom on the level of pharmacological activity is presented in Section 3.2. The results of the pharmacological activity of oleanolic acid bromolactones **9**–**14** (Figure 3), particularly the results of cytotoxic activity (Table 3), are consistent with these facts.

#### 3.3.2. Selectivity Index

The Selectivity Index (SI) is an essential indicator for single chemical substances, preparations containing two or more chemical substances, and extracts from materials of natural origin, and this indicator provides information about cytotoxicity towards cancer cells and normal cells. This indicator is the quotient of the IC_50_ value of a normal cell line (e.g., HDFs, as in the presented experiments) and the IC_50_ value of a cancer cell line. The Selectivity Index often determines further research on the cytotoxic activity of a single substance, mixture, or extract. This is because a material constituting a potential drug and exhibiting a high cytotoxic activity towards cancer cells is often highly toxic against healthy cells. The scientific literature shows that the limit value determining whether a given research material can be considered a drug candidate is 10 [55]. In turn, Valderrama states that for a single substance to be considered a drug candidate, its Selectivity Index value should be equal to or higher than 2 [56].

The Selectivity Index is expressed as a simple ratio of IC_50_ calculated for healthy and cancer cells [57]. The HDF (*human dermal fibroblast*) cell line is most often used to determine the Selectivity Index. This is due to several important features of this cell line and its usefulness in assessing the safety and effectiveness of the compound. HDFs are healthy human fibroblast cells that do not show cancerous features. Therefore, their use allows for assessing the tested compound’s potential toxicity towards healthy cells. The results obtained with HDFs may be more consistent with actual toxicity in the human body. Fibroblasts are present in the skin, mucous membranes, and other connective tissues. They are, therefore, an excellent model for assessing potential damage to healthy tissues. Research on HDFs helps assess whether the compound can be safely used in humans, minimizing the risk of damage to healthy tissue. The high SI suggests that the compound has the potential for further clinical trials because it may act selectively on cancer cells.

The IC_50_ values for oleanolic acid (**1**) and its seven lactones (**2**–**8**, Figure 2) and six bromolactones (**9**–**14**, Figure 3) that were experimentally determined for the cancer cell lines used (A-549, PC-3, and SKOV-3) and for normal fibroblasts (HDF cell line) are included in Table 3. The Selectivity Index values calculated for oleanolic acid (**1**) and its seven lactones (**2**–**8**, Figure 2) and six bromolactones (**9**–**14**, Figure 3) are listed in Table 4.

As Table 4 shows, apart from oleanolic acid (**1**), the parent compound in our experiments, drug candidates may be lactones **2** (3-acetoxy-12-hydroxylactone) and **6** (3-acetoxy-12-hydroxyiminolactone), as well as bromolactone **10** (3-oxo-12-bromolactone). For these compounds, the Selectivity Index value for almost all tested cell lines significantly exceeds 2, which, according to Valderrama’s theorem [56], is necessary to be considered as drug candidates. For the next two lactones (3,12-dihydroxylactone **3** and 3-acetoxy-12-oxolactone **4**) and one bromolactone (3-hydroxy-12-bromolactone **9**), the Selectivity Index value is close to 2 (Table 4).

Taking into account the IC_50_ values for the tested cell lines (A-549, CHO, PC-3, and SKOV-3) for lactones and bromolactones with the highest Selectivity Index value (Table 3 and Table 4), it is easy to notice that 3-acetoxy-12-hydroxylactone **2**, 3-acetoxy-12-hydroxyiminolactone **6** (Figure 2), and 3-oxo-12-bromolactone **10** (Figure 3) may be considered as excellent drug candidates for which it would be worth conducting more advanced research in the future, including the mechanism of their cytotoxic activity.

As Table 4 presents, some of the derivatives we obtained have a low Selectivity Index (e.g., compounds **7** and **8**, Figure 2). The structure of triterpene derivatives with low SI is an important indication for the future, regarding the directions of further chemical modifications of triterpene compounds. The two derivatives with the lowest IC_50_ value for all tested cell lines, and at the same time with the lowest SI value, are 3-12-dioxolactone **7** and 3-hydroxy-12-hydroxyiminolactone **8** (Figure 2 and Table 3 and Table 4). Taking into account the structure of these chemical compounds, it should be concluded that the simultaneous presence of these three functional group systems (i.e., 3-ketone + 12-ketone + olide system, compound **7**; 3-hydroxyl + 12-oxime + olide system, compound **8**) is in both cases the most unfavourable arrangement of substituents in the oleanane molecule and to obtain effective anticancer agents, the combination of probably two of the three substituents present in the derivative molecule **7** or **8** should be avoided.

#### 3.3.3. Apoptotic Index

The ability of all tested compounds to induce apoptosis was investigated with two cancer cell lines (SKOV-3 and PC-3). Cells were treated with three different concentrations of each compound (0.1, 1.0, and 10.0 µg/mL) and DNA fragmentation was determined as an indicator of apoptosis using a cell death ELISA assay. All fourteen compounds (oleanolic acid, **1**, seven lactones: **2**–**8**; Figure 2, and six bromolactones: **9**–**14**; Figure 3) induced apoptosis similarly in both cancer cell lines, with an Apoptotic Index within 4–7 (Table 5). Oleanolic acid (**1**), the internal standard for investigated triterpenoids, showed an AI of 5.16 and 5.27, respectively, for the SKOV-3 and PC-3 cell lines. For the other compounds, it was found that an increased concentration also increased apoptotic indices. The investigation has shown that compound **6** (Figure 2) was the most effective in inducing apoptosis. All these results are shown in Table 5.

### 3.4. Molecular Docking of Oleanolic Acid Lactones *(**2**–**8**)* and Bromolactones *(**9**–**14**)*

#### 3.4.1. Discussion of Molecular Docking Results

Results were obtained for oleanolic acid (**1**) and its lactones (**2**–**8**; Figure 2) and bromolactones (**9**–**14**; Figure 3). Based on the data presented in Table 7 and Table S3 (Supplementary Materials, File S3, Figures S2–S29), it can be concluded that the docking process occurs in all cavities (C1–C5) of the EGFR tyrosine kinase domain molecule (PDB ID 1M17) and that all compounds tested show an excellent ability to interact with this structure. This research focused on the two most significant pockets, C1 and C2, as they demonstrated the highest potential for ligand binding to the EGFR protein. Due to space limitations in the article and the superior binding results observed for C1 and C2, pockets C3, C4, and C5 were excluded from detailed analysis.

The top three docking results were examined for the larger C1 pocket, with a calculated volume of 991 Å^3^, with compound **6** (Figure 2) achieving the best performance, followed by compounds **9** and **11** (Figure 3). Similarly, for the smaller C2 pocket, with a volume of 665 Å^3^, the top three docking results were also evaluated, and compound **6** (Figure 2) again demonstrated the highest performance. The detailed docking results for these two prominent pockets are reported, while the findings for the remaining smaller pockets are not discussed in depth but can be found in the Supplementary Materials.

The electronegative oxygen and nitrogen atoms of the hydroxyimino moiety in compound **6** (Figure 2) facilitate the formation of conventional hydrogen bonds with residues LYS 721, ASP 831, and PHE 699. Additionally, the oxygen atoms enable van der Waals interactions (Figure 5A).

Compounds **7** and **8** (Figure 2) show lower docking values in the C1 and C2 pockets (e.g., −7.9 kcal/mol for both in C1; Table 7), corresponding to their low biological activity (IC_50_ > 100 µM; Table 3). The weaker results may be due to the absence of key functional groups enabling hydrogen bonding and hydrophobic interactions at key binding sites.

Compound **9** (Figure 3) forms halogen interactions with GLU 734 and ALA 731 and engages in pi-sigma bonding with PHE 699. The compound’s bromine and oxygen atoms also facilitate van der Waals’s formation (Figure 6).

Compound **11** (Figure 3) exhibits hydrophobic interactions with the phenylalanine residue at position 699. The electronegative oxygen atom of the carboxyl group facilitates the formation of a conventional hydrogen bond with the serine residue at position 728. In contrast, the compound’s bromine and oxygen atoms enable van der Waals interactions (Figure 7).

The C2 pocket exhibited the most promising results for compound **6**, which formed stable hydrogen bonds. Specifically, the electronegative oxygen atom engaged in a hydrogen donor interaction with the ASP 831 residue, while the nitrogen atom participated in a hydrogen acceptor bond with LYS 721 (Figure 8).

In compound **14** (Figure 3), the oxygen atom in the lactone group that is doubly bonded to the carbon forms a hydrogen bond acceptor interaction with the LYS 721 residue, while the carbon atom in the lactone group forms a hydrogen bond donor interaction with the ASP 831 residue. These hydrogen bonding interactions contribute to the stable ligand binding observed for this compound (Figure 9).

Compound **13** (Figure 3) also exhibits stable hydrogen bonding interactions. The carbonyl oxygen within the lactone moiety participates in a hydrogen acceptor bond with the LYS 721 residue (Figure 10).

#### 3.4.2. Correlation of Molecular Docking Results, Biological Activity, and Chemical Modifications

The correlation between molecular docking results, biological activity, and chemical modifications is a key issue in drug design and research on biologically active compounds. Molecular docking is a computational method used to predict interactions between small molecules (ligands) and target macromolecules (e.g., proteins and nucleic acids). This study aims to assess the binding strength of a ligand to the active site of a biological target and identify potential mechanisms of action and key molecular interactions (e.g., hydrogen bonds and hydrophobic interactions). Ideally, low binding energy in docking (high stability of the ligand–receptor complex) correlates with high biological activity. Docking does not always consider dynamic changes in the protein structure and the surrounding environment (e.g., interactions in the aquatic environment). Furthermore, despite good docking results, pharmacokinetic barriers (e.g., solubility and metabolism) may limit the activity. Docking results indicate key interactions that can be optimized through targeted chemical modifications.

The correlation of molecular docking results, biological activity, and chemical modifications of the obtained oleanolic acid lactones (**2**–**8**; Figure 2) and bromolactones (**9**–**14**; Figure 3) covers the following areas:

- **Accuracy and repeatability of docking**: Molecular docking was performed rigorously. For each compound, docking was performed three times in two significant pockets (C1 and C2) of the EGFR protein (PDB: 1M17). The results presented in the tables are the average of these replicates, ensuring their reliability. Using the CB-Dock2 server, based on machine learning algorithms, represents a modern approach to docking analysis. Unlike traditional methods such as AutoDock or Vina, CB-Dock2 automatically identifies the optimal binding pockets, which minimises subjective user decisions and increases the precision of the results.
- **Correlation between docking and in vitro results**: The two best examples of correlation between molecular docking results and in vitro results are as follows:
  ○ Compound **6** (Figure 2) achieved the highest binding energy in the C1 (−9.8 kcal/mol) and C2 (−10.3 kcal/mol) pocket (Table 7) and showed the best IC_50_ values in the cytotoxic assay (1.27 µM for line A-549; Table 3). Strong hydrogen bonds with LYS 721 and ASP 831 and van der Waals interactions with protein residues explain its high activity.
  ○ Compound **10** (Figure 3) achieved a high docking score in the C2 pocket (−9.7 kcal/mol; Table 7), which correlates with its moderate biological activity (IC_50_ = 15.55 µM for line A-549; Table 3). Interactions with GLU 734 and ALA 731 and halogen interactions may be key to its activity.
- **Correlation between docking and structural design**: The structural modifications of the compounds were not accidental but resulted from rational design:
  ○ A hydroxyimine group (C=NOH) at the C-12 position, as in compound **6** (Figure 2), was introduced to increase the number of possible hydrogen bonds with key residues of the EGFR protein. Docking results confirm that such bonds (e.g., with ASP 831) are responsible for the high activity of this compound.
  ○ The bromine atom in the bromolactones **9**–**14** (Figure 3) was introduced to increase lipophilicity and allow additional halogen interactions. In the case of compound **10**, the bromine enhances interactions with the C2 pocket, as reflected in the docking results.
- **A critical approach to the results**: As Table 3, Table 4 and Table 5 and Figure 11 present, not all compounds showed high biological activity, which is natural in finding new drugs. However, the lack of activity of some compounds (e.g., compounds **7** and **8**; Figure 2) provided valuable information on the structural determinants of activity. The results indicate that the presence of two carbonyl groups in compound **7** reduces the ability to bind to EGFR. Moreover, the absence of the corresponding hydrogen bond-donating groups in compound **8** explains its low activity.

### 3.5. Antioxidant Activity of Oleanolic Acid Lactones *(**2**–**8**)* and Bromolactones *(**9**–**14**)*

The antioxidant activity results for compounds **1**–**14** (Figure 2 and Figure 3), measured using both the CUPRAC (cupric ion reducing antioxidant capacity) and DPPH (2,2-diphenyl-1-picrylhydrazyl hydrate) assays [42], are presented on Figure 11, and these results reveal significant variation in performance, underscoring the complex nature of their antioxidant mechanisms.

In the CUPRAC assay, 3,12-dioxolactone **7** exhibits the highest antioxidant activity (0.23347 ± 0.00959), indicating electron-donating solid capabilities essential for reducing copper(II) ions in this assay. Other compounds, such as 3-acetoxy-12-hydroxylactone **2** (0.16606 ± 0.00697) and 3-hydroxy-12-hydroxyiminolactone **8** (0.16382 ± 0.00896), also show relatively high CUPRAC activity (Figure 11), suggesting their structural features (probably the C-12 hydroxy group; Figure 2) are well-suited for electron transfer. In contrast, compounds such as **3** (3,12-dihydroxylactone; 0.04021 ± 0.0018) and **12** (A-nitrilo-12-bromolactone; 0.03606 ± 0.00209) exhibit much lower CUPRAC activity (Figure 11), indicating they are less efficient in redox reactions. In the DPPH assay, compound **10** (3-oxo-12-bromolactone) shows the highest antioxidant activity (0.03591 ± 0.00135), reflecting a solid ability to donate hydrogen atoms to neutralise free radicals. Other compounds, including **3** (3,12-dihydroxylactone; 0.03338 ± 0.00125) and **9** (3-hydroxy-12-bromolactone; 0.02780 ± 0.00104), demonstrate significant DPPH activity (Figure 11), highlighting their efficacy in hydrogen atom transfer. On the other hand, 12-oxolactones **4** (0.01918 ± 0.000718) and **5** (0.01931 ± 0.000723) have comparatively lower DPPH activity, suggesting their structures are less favourable for this mechanism.

Several compounds show divergent activity profiles when comparing results between the CUPRAC and DPPH assays. For example, 3,12-dioxolactone **7** (Figure 2), which had the highest CUPRAC activity, shows only moderate DPPH activity (0.02130 ± 0.000797). This result suggests that its antioxidant mechanism primarily involves electron transfer rather than hydrogen donation. Conversely, 3-oxo-12-bromolactone **10** (Figure 3), which exhibited the most robust DPPH activity, shows moderate CUPRAC activity (0.14919 ± 0.00757), indicating a stronger preference for hydrogen atom donation. Compounds such as **1** (oleanolic acid) and **1a** (3-acetoxyoleanolic acid; Figure 2) show balanced activities across both assays, with **1a** demonstrating 0.16603 ± 0.00938 in CUPRAC and 0.02414 ± 0.000903 in DPPH, suggesting that these compounds have versatile antioxidant mechanisms involving both electron transfer and hydrogen atom donation. In contrast, 3,12-dihydroxylactone **3** (Figure 2) shows a significant discrepancy between the two assays, with low CUPRAC activity (0.04021 ± 0.0018) and relatively high DPPH activity (0.03338 ± 0.00125), indicating a preference for radical scavenging over electron transfer.

In summary, the antioxidant activity of these oleanolic acid derivatives varies significantly depending on the assay, reflecting different underlying mechanisms. Some compounds, such as 3,12-dioxolactone **7** (Figure 2), excel in electron transfer as measured by the CUPRAC assay, while others, like 3-oxo-12-bromolactone **10** (Figure 3), are more effective in hydrogen atom donation, as reflected in the DPPH assay. These differences underscore the importance of evaluating antioxidant activity using multiple assays to fully capture the complexity of these compounds’ mechanisms of action.

Determining the antioxidant activity of chemical substances is of key importance in designing pharmacologically active substances to prevent and treat diseases and in designing cosmetic ingredients and nutrients. These substances can support the body’s natural defence mechanisms, counteract the effects of oxidative stress, and improve the quality of life. The two most popular and most commonly used tests for this purpose are the cupric ion reducing (CUPRAC) and the 2,2-diphenyl-1-picrylhydrazyl (DPPH) antioxidant capacity assays [58]. The task of both techniques is to assess the ability of a chemical compound to scavenge radicals. Since the mentioned tests differ in their mechanism of action, a difference in the activity of the same substance subjected to both tests is observed.

The differences in the results obtained by the CUPRAC and DPPH methods for the same chemical substance result not only from the different mechanisms of these two reactions but also from the differences like the radicals and oxidants, the polarity and partition behaviour of the antioxidant compounds within the sample matrix, and the kinetics of the antioxidant–radical interactions [58,59,60].

The CUPRAC (Cupric Reducing Antioxidant Capacity) test assesses the antioxidant capacity of chemical substances. It involves measuring the ability of an antioxidant to reduce copper(II) (Cu^2+^) to copper(I) (Cu^+^) in the presence of neocuproine (2,9-dimethyl-1,10-phenanthroline), which forms a stable complex with an intense yellow colour with Cu+ ions. The CUPRAC test has a wide range of applications: it can assess the antioxidant capacity of both hydrophilic and lipophilic compounds [58].

The DPPH (2,2-diphenyl-1-picrylhydrazyl) test is also used to assess chemical compounds’ antioxidant capacity, but based on a different mechanism. It involves measuring the ability of an antioxidant to neutralize the stable DPPH^●^ radical, which is reduced during the reaction and can be assessed spectrophotometrically. The reaction mechanism involves the transfer of an electron or hydrogen atom from the antioxidant to the DPPH^●^ radical. The DPPH^●^ free radical has an intense purple colour, is stable, and absorbs light at a wavelength of approximately 517 nm. After donating an electron or a hydrogen atom by the antioxidant, DPPH^●^ is reduced to the DPPH-H form, which leads to the disappearance of the purple colour and reduced absorption [58].

Our research results show that hydroxyl groups present in the structures of compounds such as **2** and **3** (Figure 2) promote electron transfer in the CUPRAC method. This happens because hydroxyl groups can interact with Cu(II) ions, reducing them to Cu(I). This enables these compounds to participate in redox reactions requiring electron transfer effectively.

In turn, carbonyl groups and bromine atoms, such as those present in compound **10** (Figure 3), prefer the mechanism of donating hydrogen atoms, which is crucial in neutralizing DPPH radicals. The carbonyl group stabilizes free radicals through a resonance mechanism, which helps neutralize radicals. The bromine atom enhances this effect by stabilizing the redox reaction products thanks to its electrophilic properties and the ability to form hydrogen bonds. The difference in the results of both tests (CUPRAC and DPPH) emphasizes that antioxidant activity strictly depends on the compound’s structure and the type of redox reaction [58,59,60].

### 3.6. ADMETox Analysis of Oleanolic Acid Lactones *(**2**–**8**)* and Bromolactones *(**9**–**14**)*

Each newly obtained chemical substance, which has a chance to become a drug, should not only show the appropriate level of the desired pharmacological activity, but also be safe to use, efficient, and have a favourable pharmacokinetic profile (ADMET properties). As such, there is a need to predict the ADMET (Adsorption, Distribution, Metabolisms, Excretion, and Toxicity) properties during the early stages of development to increase the success rate of compounds that reach the lead optimisation process. The results of computer calculations regarding ADMETox parameters obtained for the oleanolic acid lactones (**2**–**8**, Figure 2) and bromolactones (**9**–**14**, Figure 3) are presented in Supplementary Materials, File S2, Tables S1 and S2.

The parent compound (**1**) and its lactones (**2**–**8**, Figure 2) and bromolactones (**9**–**14**, Figure 3) demonstrated favourable physicochemical properties, with only a few cases showing moderately favourable parameters (e.g., molecular weight, nHA, nHD, nRot, nRing, nHet, fChar, nRig, flexibility, and TPSA; Supplementary Materials, File S2, Tables S1 and S2). Because of the low solubility of the above compounds in water, the value of logS, logP, and logD was moderately favourable.

The medicinal chemistry parameters suggest that all tested lactones (**2**–**8**, Figure 2) and bromolactones (**9**–**14**, Figure 3) are easy to synthesise, as the Synthetic Accessibility value is around 6. Other parameters of this group suggest a high level of novelty that follows the trends currently observed in medicinal chemistry. The NP value (Natural Product-likeness) confirms a high similarity to compounds **2**–**14** (Figure 2 and Figure 3) of natural origin (from which these triterpenes were obtained). PAINS, BMS, and Chelator tests are negative for almost all tested triterpenes, which means that there are no unfavourable elements of the structure of the molecules of these substances, which can be potentially responsible for toxicity or may, for example, enter into chemical interaction with other chemical substances present in the body.

The information contained in Supplementary Materials, File S2, Tables S1 and S2 clearly shows that the transformation of the C-17 carboxyl group of oleanolic acid (**1**) into the lactone system (derivatives **2**–**8**, Figure 2) or bromolactone system (derivatives **9**–**14**, Figure 3) and the introduction of some groups functionalities can influence the ability to inhibit Pgp: the ADMETlab Manual (2.0) program [61] predicts that oleanolic acid (**1**) can be both an excellent Pgp inhibitor (p-glycoprotein inhibitor) and a Pgp substrate, while its lactones (**2**–**8**, Figure 2) and bromolactones (**9**–**14**, Figure 3) can only be excellent Pgp substrates. A p-glycoprotein substrate is a substance that uses the P-glycoprotein transporter for various activities, including drug absorption, drug excretion, and other important activities that can lead to changes in the body or changes in the effects of other drugs on the body [62].

The oleanolic acid lactones we have presented (**2**–**8**, Figure 2) may also be excellent CYP2C19 and CYP3A4 substrates and CYP3A4 inhibitors. In turn, most bromolactones (**9**–**14**, Figure 3) may be excellent substrates of cytochromes CYP1A2, CYP2C19, and CYP3A4 or inhibitors of cytochromes CYP2C9 and CYP3A4. Cytochrome inhibitors inhibit the activity of cytochrome P450 (CYP) family enzymes. These enzymes play a key role in metabolising many drugs, toxins, and other chemicals in the body. Inhibiting their activity may affect the pharmacokinetics of substances, increasing their concentration in the body, which may lead to side effects or toxicity. In turn, CYP substrates are chemical compounds that are metabolised by enzymes from the cytochrome P450 (CYP) family. These substrates may include drugs, toxins, endogenous metabolites (e.g., hormones and fatty acids), and other xenobiotics. Cytochromes P450 play a key role in the biotransformation of these compounds, converting them into more polar metabolites that can be more easily excreted from the body [63].

Most of the tested derivatives (**2**–**14**, Figure 2 and Figure 3) show, with a few exceptions, favourable or quite favourable absorption and distribution parameters, which indicates their good permeability and migration throughout the body. As indicated by the subsequent parameters of the ADMETox tests, the tested derivatives **2**–**14** (Figure 2 and Figure 3) should be appropriately metabolised and excreted, which, combined with low toxicity (apart from aquatic toxicity and respiratory toxicity), is further evidence that the derivatives **2**–**14** (Figure 2 and Figure 3) may in the future become good drug candidates.

### 3.7. Challenges in Translating Natural Compound Derivatives to Clinical Settings

Translating derivatives of natural compounds into clinical settings involves numerous challenges that result from their chemical nature, mechanism of action, pharmacokinetic and pharmacodynamic processes, and regulatory requirements. The most important challenges include [64,65,66].

1. **Complex chemical structures**: the structures of derivatives of natural compounds are often characterized by a complicated structure. This factor may significantly complicate the synthesis of these derivatives on a large scale. The synthesis of such compounds often requires a complex cycle of chemical transformations, requiring expensive, hard-to-access or dangerous reagents and solvents, and the process of synthesizing, isolating and purifying these derivatives can be time-consuming, labour-intensive and energy-intensive. Obtaining high-purity compounds is time-consuming and expensive but, at the same time, crucial for clinical applications. In the case of the triterpene derivatives we presented, there is no need to use multi-stage chemical reaction systems, and the reagents used are readily available, low-cost and safe to use. Our optimization of the synthesis methods of these compounds allows us to obtain them in quantities necessary not only for micro-scale research but, if necessary, on a much larger scale.
2. **Low bioavailability**: Many natural compound derivatives have poor solubility in water, which limits their absorption from the gastrointestinal tract. Moreover, such compounds may be unstable in a physiological environment (e.g. in the presence of enzymes or low pH). The high susceptibility to first-pass metabolism in the liver limits their availability in general circulation.
3. **Pharmacokinetic and pharmacodynamic problems**: Many semi-synthetic derivatives have a short half-life in the body, requiring frequent administration or controlled release systems. Such derivatives may bind to plasma proteins in an unfavourable manner, which limits their biological activity. In addition, some natural compounds have low specificity of action, which may lead to side effects.
4. **Toxicity and side effects**: Many natural compound derivatives are characterized by a narrow therapeutic index, a narrow margin of safety between the therapeutic and toxic doses. There may be unexpected toxic effects in the body that were not apparent in preclinical studies.
5. **Technological challenges**: Extraction and purification of natural compounds from plant raw materials or microorganisms can be complicated and expensive. The production of such compounds on a large scale in industrial conditions is difficult, especially for compounds obtained from rare or difficult-to-access sources. Moreover, developing appropriate drug forms (e.g. capsules, tablets, injections) requires additional research and technology.
6. **Complexity of preclinical and clinical studies:**
  ○ In vitro and in vivo modelling: Difficulty in creating appropriate biological models to study the activity and toxicity of natural compounds.
  ○ Transfer to clinical trials: Natural compounds may be potent in the laboratory, but their effectiveness in living organisms may be limited.
  ○ Durability of clinical trials: Conducting clinical trials (phase I-III) is time-consuming and expensive, especially for compounds with uncertain therapeutic potential.
7. **Regulatory issues**:
  ○ Registration and approval: Registration requirements for natural medicines are stringent, especially regarding safety, effectiveness and quality.
  ○ Standardization: Difficulties in standardizing natural compounds, especially when obtained from different sources (e.g. different plant species).
  ○ Intellectual property: Problems related to patent protection of natural compounds that have often been known for a long time and may be considered unpatentable.
8. **Sustainable sourcing of raw materials**: Natural compounds that are the subject of research by scientists in many fields of science often come from rare plants, microorganisms or marine organisms, the exploitation of which may be irreversible.

Natural compounds and their semi-synthetic derivatives, including triterpene derivatives, remain a valuable source of pharmacologically active substances despite numerous challenges. Their therapeutic potential is enormous, but their implementation into clinical practice requires interdisciplinary cooperation (chemistry, pharmacology, biotechnology, medicine). Advances in chemical engineering, computer modelling, and biotechnology are helping to overcome many of these barriers.

## 4. Materials and Methods

### 4.1. Materials

#### 4.1.1. NMR

The ^1^H- and ^13^C-NMR spectra of oleanolic acid lactones **2**–**8** (Figure 2) and bromolactones **9**–**14** (Figure 3) were recorded using CDCl_3_ as a solvent with tetramethylsilane (TMS) as an internal standard (Sigma-Aldrich^®^, Darmstadt, Germany).

#### 4.1.2. Syntheses, Column Chromatography, TLC and HP TLC and Antioxidant Activity

Oleanolic acid (**1**) was purchased from Bio-Tech^®^ (Beijing, China). All commercially available solvents and reagents used in our experiments were graded “pure for analysis” (Sigma-Aldrich^®^, Darmstadt, Germany; Fluka^®^, Charlotte, NC, USA; Chempur^®^, Piekary Śląskie, Poland; and POCh^®^, Gliwice, Poland). The solvents were dried according to the usual procedures. Column chromatography for the separation and/or purification of obtained derivatives was performed with silica gel 70–230 mesh (Merck Millipore, Darmstadt, Germany). TLC and HP TLC analyses were performed with silica gel aluminium sheets (Merck Millipore, Darmstadt, Germany).

#### 4.1.3. MTT Assay

All the cell lines and mediums were obtained from the American Type Culture Collection (ATCC), supplied by LGC-Standards (Lomianki, Poland).

#### 4.1.4. Apoptosis

The induction of apoptosis by tested compounds was determined with Cell Death Detection ELISA Plus from Roche Diagnostics GmbH (Mannheim, Germany).

#### 4.1.5. Antioxidant Activity

The CUPRAC reagent (7.5 mM ethanolic 96% neocuproine solution, 10 mM CuCl_2_ × H_2_O solution, and an ammonium acetate buffer of pH 7.0) and DPPH reagent (2,2-diphenyl-1-picrylhydrazyl 0.2 mM solution) were applied.

### 4.2. Methods

#### 4.2.1. NMR Spectra of Oleanolic Acid Lactones **2**–**8** and Bromolactones **9**–**14**

The ^1^H- and ^13^C-NMR spectra were recorded using a Bruker Advance 600 MHz spectrometer (Billerica, MA, USA). Chemical shifts (δ) were expressed in parts per million (ppm) relative to tetramethylsilane (TMS) as an internal standard, using CDCl_3_ as a solvent (unless written otherwise). Coupling constants (*J*) are expressed in Hertz (Hz).

#### 4.2.2. Synthesis of Oleanolic Acid Lactones **2**–**8** and Bromolactones **9**–**14**

Products were purified by column chromatography. The progress of the reaction and the preliminary evaluation of products’ purity were made by the TLC method. The chromatograms were developed in mixtures of C_6_H_6_ and AcOEt in different volume ratios, given in brackets (e.g., 2:1). The chromatograms were then visualised by spraying them with 10% solution of H_2_SO_4_ in EtOH and heating them at 110 °C for about 5 min. The spots deriving from individual compounds were observed in VIS. Melting points were measured with the Büchi apparatus in an open capillary and were uncorrected.

#### 4.2.3. General Procedures

**Procedure A. Alkaline hydrolysis**: A saturated solution of 1.0 mmol of acetoxytriterpene in a 5% ethanolic NaOH solution was refluxed until the reaction was completed (TLC control). The post-reaction mixture was cooled and poured into 6 times the volume of water. The formed precipitate was filtered off, washed with water, dried, and then crystallised.

**Procedure B. Jones Oxidation**: To a saturated solution of 1.0 mmol of hydroxytriterpene in acetone, with gentle stirring, an excess of Jones’ reagent was added dropwise, then gently stirred at room temperature until the reaction was completed (TLC control). The excess of oxidising reagent was decomposed by adding isopropyl alcohol dropwise. The post-reaction mixture was poured into 6 times the volume of water. The formed precipitate was filtered off, washed with water, dried, and then crystallised.

**Procedure C. Oxime formation**: To a hot saturated solution of 1.0 mmol of oxotriterpene in ethanol, 347 mg (5.0 mmol) of hydroxylamine hydrochloride was added, followed by the addition of 656 mg (8.0 mmol) of anhydrous sodium acetate, and the solution was heated in reflux until the reaction was completed (TLC control). The post-reaction mixture was cooled and poured into 6 times the volume of water. The formed precipitate was filtered off, washed with water, dried, and then crystallised.

#### 4.2.4. Obtaining of Oleanolic Acid Lactones **2**–**8** and Bromolactones **9**–**14**

**3β-Acetoxyolean-12-en-28-oic acid (1a):** Synthesis was performed based on literature protocol [54].

**R_f_**: 0.91 (1:1); 0.85 (2:1); 0.79 (4:1); 0.55 (9:1); 0.37 (15:1); 0.27 (25:1); pink-brown spot. **Yield**: 486 mg (97.5%). **M.p.**: 267–268 °C (EtOH), lit. m.p.: 268–269 °C [67]. Spectral data are consistent with the literature [67]. **HR MS**: For M^+•^ ion of composition C_32_H_50_O_4_ calcd.: 498.3709, obtd.: 493.711. **Elem. anal.**: calcd.: C% = 77.06; H% = 10.10; obtd.: C% = 77.05; H% = 10.12.

**3β-Acetoxy-12α-hydroxyolean-28-oic acid 28β→13β-olide** (**2**): Synthesis was conducted according to the procedure given elsewhere [46].

**R_f_**: 0.66 (4:1); 0.31 (9:1); grey-brown spot. **Yield**: 312 mg (60.6%). **M.p.**: 302–303 °C (EtOH), lit. m.p. 301–303 °C [68]. Spectral data are consistent with the literature [43]. **HR MS**: For M^+•^ ion of composition C_32_H_50_O_5_ calcd.: 514.3658, obtd.: 514.3652. **Elem. anal.**: calcd.: C% = 74.67; H% = 9.79; obtd.: C% = 74.66; H% = 9.82.

**3β,12α-Dihydroxyolean-28-oic acid 28β→13β-olide** (**3**): Synthesis was conducted according to Procedure A, starting with 515 mg (1.0 mmol) of 3β-acetoxy-12α-hydroxyolean-28-oic acid 28β→13β-olide (**2**).

**R_f_**: 0.93 (EtOH); 0.78 (AcOEt); 0.58 (1:1); 0.33 (2:1); 0.20 (4:1); 0.06 (9:1); 0.03 (15:1); yellow-brown spot. **Yield**: 486 mg (97.5%). **M.p.**: 247–248 °C (EtOH), lit. m.p.: 247–249 °C [43]. Spectral data are consistent with literature data [43]. **HR MS**: For M^+•^ ion of composition C_30_H_48_O_4_ calcd.: 472.3553, obtd.: 472.3555. **Elem. anal.**: calcd.: C% = 76.23; H% = 10.23; obtd.: C% = 76.25; H% = 10.22.

**3β-Acetoxy-12-oxoolean-28-oic acid 28β→13β-olide** (**4**): Synthesis was conducted according to the procedure given elsewhere [46], with the application of Procedure B, starting with 515 mg (1.0 mmol) of 3β-acetoxy-12α-hydroxyolean-28-oic acid 28β→13β-olide (**2**).

**R_f_**: [43]. **Yield**: 471 mg (92.0%). **M.p.**: 289–290 °C (EtOH, decomp.), lit. m.p.: 287–290 °C (decomp.) [46]; lit. m.p.: 287–289 °C [43]. Spectral data are consistent with the literature [46]. **HR MS**: For M^+•^ ion of composition C_32_H_48_O_5_ calcd.: 512.3502, obtd.: 512.3508. **Elem. anal.**: calcd.: C% = 74.96; H% = 9.44; obtd.: C% = 74.99; H% = 9.45.

**3β-Hydroxy-12-oxoolean-28-oic acid 28β→13β-olide** (**5**): Synthesis was conducted according to Procedure A, starting with 512 mg of 3β-acetoxy-12-oxoolean-28-oic acid 28β→13β-olide (**4**).

**R_f_**: 0.76 (1:1); 0.61 (2:1); 0.46 (4:1); 0.23 (9:1); 0.11 (15:1); 0.07 (25:1); yellow-brown spot. **Yield**: 449 mg (95.5%). **M.p.**: 290–291 °C (EtOH). **^1^H NMR** (δ): 3.20 (1H, dd, *J* = 4.3 and 11.4 Hz, C_3_-H_α_); 2.71 (1H, t, *J* = 14.3 Hz, C_18_-H_β_); 1.32, 1.00, 0.98, 0.97, 0.96, 0.91, 0.79 (7 × 3H, 7 × s, 7 × CH_3_ group). **^13^C NMR** (δ): 206.2 (C_q_, C-12); 178.5 (C_q_, C-28); 91.0 (C_q_, C-13); 78.4 (CH, C-3); 43.9 (C_q_, C-17). **DEPT**: 7 × CH_3_, 10 × CH_2_, 4 × CH, 30 × C atom. **HR MS**: For M^+•^ ion of composition C_30_H_46_O_4_ calcd.: 470.3396, obtd.: 470.3399. **Elem. anal.**: calcd.: C% = 76.55; H% = 9.85; obtd.: C% = 76.58; H% = 9.84.

**3β-Acetoxy-12-hydroxyiminoolean-28-oic acid 28β→13β-olide** (**6**): Synthesis was conducted according to procedure given elsewhere [46], with the application of Procedure C, starting with 512 mg of 3β-acetoxy-12-oxoolean-28-oic acid 28β→13β-olide (**4**).

**R_f_**: [46]. **Yield**: 486 mg (92.2%). **M.p.**: 229–231 °C (EtOH), lit. m.p.: 228–231 °C [46]. Spectral data are consistent with the literature [46]. **HR MS**: For M^+•^ ion of composition C_32_H_49_NO_5_ calcd.: 527.3610, obtd.: 527.3611. **Elem. anal.**: calcd.: C% = 72.83; H% = 9.36; N% = 2.65; obtd.: C% = 72.82; H% = 9.35; N% = 2.66.

**3,12-Dioxoolean-28-oic acid 28β→13β-olide** (**7**): Synthesis was conducted according to Procedure B, starting with 470 mg of 3β-hydroxy-12-oxoolean-28-oic acid 28β→13β-olide (**5**).

**R_f_**: 0.90 (1:1); 0.85 (2:1); 0.83 (4:1); 0.53 (9:1); 0.33 (15:1); 0.24 (25:1); brown-yellow spot. **Yield**: 427 mg (91.1%). **M.p.**: 273–275 °C (EtOH). **^1^H NMR** (δ): 2.78 (1H, t, *J* = 14.3 Hz; C_18_-H_β_); 1.37, 1.10, 1.06, 1.04, 0.99, 0.98, 0.97 (7 × 3H, 7 × s, 7 × CH_3_ group). **^13^C NMR** (δ): 216.4 (C_q_, C-3); 205.5 (C_q_, C-12); 178.3 (C_q_, C-28); 90.9 (C_q_, C-13); 44.1 (C_q_, C-17); 21.2 (CH_3_, CH_3_COO). **DEPT**: 5 × CH_3_ + 1 × 2CH_3_ (6 signals, 7 CH_3_ groups), 10 × CH_2_, 3 × CH, 30 × C atom. **HR MS**: For M^+•^ ion of composition C_30_H_44_O_4_ calcd.: 468.3240, obtd.: 468.3239. **Elem. anal.**: calcd.: C% = 76.88; H% = 9.46; obtd.: C% = 76.86; H% = 9.45.

**3β-Hydroxy-12-hydroxyiminoolean-28-oic acid 28β→13β-olide** (**8**): Synthesis was conducted according to Procedure A, starting with 527 mg of 3β-acetoxy-12-hydroxyiminoolean-28-oic acid 28β→13β-olide (**6**).

**R_f_**: 0.93 (acetone); 0.75 (AcOEt); 0.64 (1:1); 0.41 (2:1); 0.26 (4:1); 0.05 (9:1), brown-yellow spot. **Yield**: 463 mg (95.5%). **M.p.**: 193–196 °C (precipt. from EtOH sol.). **^1^H NMR** (DMSO-*d*_6_, δ): 10.29 (1H, s, =NOH); 3.21 (1H, dd, *J* = 4.3 and 11.4 Hz, C_3_-H_α_); 2.92 (1H, t, *J* = 8.2 Hz; C_18_-H_β_); 0.84, 0.83, 0.82, 0.78, 0.77 × 2, 0.60 (5 × 3 + 1 × 6H, 6 × s, 7 × CH_3_ group). **^13^C NMR** (DMSO-*d*_6_, δ): 177.2 (C_q_, C-28); 156.2 (C_q_, C-12); 91.6 (C_q_, C-13); 77.1 (CH, C-3); 44.1 (C_q_, C-17). **DEPT**: 7 × CH_3_, 10 × CH_2_, 4 × CH, 30 × C atom. **HR MS**: For M^+•^ ion of composition C_30_H_47_NO_4_ calcd.: 485.3505, obtd.: 485.3509. **Elem. anal.**: calcd.: C% = 74.19; H% = 9.75; N% = 2.88; obtd.: C% = 74.22; H% = 9.77; N% = 2.85.

**12α-Bromo-3β-hydroxyolean-28-oic acid 28β→13β-olide** (**9**): To a stirred saturated solution of 564 mg (1.0 mmol) oleanolic acid (**1**) in glacial acetic acid, a solution of 118 mg (0.043 mL, 1.5 mmol) bromine in 3 mL glacial acetic acid was added dropwise and stirred at the room temperature until the reaction was completed (TLC control). The post-reaction mixture was poured into 6 times the volume of water. The separated precipitate was filtered off, washed with water, dried, and then crystallised.

**R_f_**: 0.86 (1:1); 0.77 (2:1); 0.68 (4:1); 0.64 (9:1); 0.29 (15:1); 0.18 (25:1); brown spot. **Yield**: 471 mg (88.3%). **M.p.**: 239–240 °C (EtOH), lit. m.p.: 225–226 °C [69]). Spectral data are consistent with the literature [69,70]. **HR MS**: For M^+•^ ion of composition C_30_H_47_BrO_3_ calcd.: 534.2708, obtd.: 534.2811. **Elem. anal.**: calcd.: C% = 67.28; H% = 8.84; obtd.: C% = 67.24; H% = 8.80.

**12α-Bromo-3-oxoolean-28-oic acid 28β→13β-olide** (**10**): Synthesis was conducted according to Procedure B, starting with 534 mg of 12α-bromo-3β-hydroxyolean-28-oic acid 28β→13β-olide (**9**).

**R_f_**: 0.90 (1:1); 0.89 (2:1); 0.85 (4:1); 0.68 (9:1); 0.54 (15:1); 0.33 (25:1); brown spot. **Yield**: 510 mg (95.8%). **M.p.**: 244–245 °C (EtOH), lit. m.p.: 238–241 °C [69]. **^1^H NMR** (δ): As in the literature [69]. **^13^C NMR** (δ): 217.6 (CH, C-3), 178.7 (C_q_, C-28), 91.5 (C_q_, C-13), 56.2 (CH, C-12), 44.8 (C_q_, C-17). **DEPT**: 7 × CH_3_, 10 × CH_2_, 4 × CH, 30 × C atom. **HR MS**: For M^+•^ ion of composition C_30_H_45_BrO_3_ calcd.: 532.2552, obtd.: 532.2558. **Elem. anal.**: calcd.: C% = 67.53; H% = 8.50; obtd.: C% = 67.50; H% = 8.52.

**12α-Bromo-3-hydroxyiminoolean-28-oic acid** (**11**): Synthesis was conducted according to Procedure B, starting with 523 mg of 12α-bromo-3-oxoolean-28-oic acid 28β→13β-olide (**10**).

**R_f_**: 0.90 (1:1); 0.86 (2:1); 0.76 (4:1); 0.51 (9:1); 0.29 (15:1); 0.19 (25:1); brown-purple spot. **Yield**: 500 mg (91.3%). **M.p.**: 267–269 °C (EtOH, decomp.). **^1^H NMR** (δ): 9.43 (1H, s, =NOH); 4.30 (1H, t, *J* = 1.9 Hz; C_12_-H_β_); 2.33 (1H, t, *J* = 4.5 Hz; C_18_-H_β_); 1.44, 1.26, 1.18, 1.08, 1.00, 0.99, 0.91 (7 × 3H, 7 × s, 7 × CH_3_ group). **^13^C NMR** (δ): 178.8 (C_q_, C-28); 166.5 (C_q_, C-3); 91.6 (C_q_, C-13); 56.3 (C_q_, C-12); 45.1 (C_q_, C-17). **DEPT**: 7 × CH_3_, 10 × CH_2_, 4 × CH, 30 × C atom. **HR MS**: For M^+•^ ion of composition C_30_H_46_BrNO_3_ calcd.: 547.2661, obtd.: 547.2662. **Elem. anal.**: calcd.: C% = 65.68; H% = 8.45; N% = 2.55; obtd.: C% = 65.65; H% = 8.47; N% = 2.56.

**12α-Bromo-3-nitrilo-3,4-secoolean-4(23)-ene-28-oic acid 28β→13β-olide** (**12**) and **12α-bromo-3-oxo-3a-aza-A-homoolean-28-oic acid 28β→13β-olide** (**13**): To a saturated solution of 547 mg (1.0 mmol) of 12α-bromo-3-hydroxyiminoolean-28-oic acid 28β→13β-olide (12), 460 mg (0.28 mL, 3.0 mmol) of POCl_3_ (phosphorus oxychloride) was added and left at room temperature overnight (TLC control). The post-reaction mixture was poured into 6 times the volume of 5% solution of HCl. The separated precipitate was filtered off, washed with water, dried, and then subjected to column chromatography on silica gel, using methylene chloride and next ethyl acetate as an eluent.

**Compound 12**: **R_f_**: 0.91 (1:1); 0.90 (2:1); 0.87 (4:1); 0.81 (9:1); 0.71 (CHCl_3_); 0.49 (15:1); 0.56 (25:1); brown-purple spot. **Yield**: 130 mg (23.8%, in conversion to starting oxime). **M.p.**: 243–245 °C (EtOH). **^1^H NMR** (δ): 4.93 (1H, t, *J* = 1.5 Hz, C_23_-H_a_); 4.71 (1H, t, *J* = 0.5 Hz; C_23_-H_b_); 4.34 (1H, dd, *J* = 3.9 and 2.3 Hz, C_12_-H_β_); 2.33 (1H, t, *J* = 4.7 Hz, C_18_-H_β_); 1.75 (3H, s, C_24_-H_3_); 1.45, 1.27, 1.00, 0.91, 0.90 (5 × 3H, 5 × s, 5 × CH_3_ group). **^13^C NMR** (δ): 178.5 (C_q_, C-28); 146.3 (C_q_, C-4); 120.2 (C_q_, C-3); 114.6 (CH_2_, C-23); 91.2 (C_q_, C-13); 56.2 (C_q_, C-12); 45.4 C_q_, (C-17). **DEPT**: 6 × CH_3_, 11 × CH_2_, 4 × CH; 30 × C atom. **HR MS**: for M^+•^ ion of composition C_30_H_44_BrNO_2_ calcd.: 529.2555, obtd.: 529.2557. **Elem. anal.**: calcd.: C% = 67.91; H% = 8.36; N% = 2.64; obtd.: C% = 67.90; H% = 8.34; N% = 2.66.

**Compound 13**: **R_f_**: 0.82 (acetone); 0.78 (EtOH); 0.46 (AcOEt); 0.35 (1:1); 0.16 (4:1); 0.02 (9:1); violet spot. **Yield**: 359 mg (65.7%, in conversion to starting oxime). **M.p.**: 278–279 °C (EtOH). **^1^H NMR** (δ): 5.66 (1H, s, N-H, lactam); 4.30 (1H, t, *J* = 3.0 Hz; C_12_-H_β_); 2.32 (1H, t, *J* = 4.9 Hz; C_18_-H_β_); 1.43, 1.33, 1.26, 1.22, 1.10, 0.99, 0.90 (7 × 3H, 7 × s, 7 × CH_3_ group). **^13^C NMR** (δ): 178.4 (C_q_, C-28); 176.02 (C_q_, C-3); 91.4 C_q_, (C-13); 56.4 (C_q_, C-12); 45.5 (C_q_, C-17). **DEPT**: 7 × CH_3_, 10 × CH_2_, 4 × CH; 30 × C atom. **HR MS**: for M^+•^ ion of composition C_30_H_46_NO_3_Br calcd.: 547.2661, obtd.: 547.2639; **Elem. anal.**: calcd.: C% = 65.68; H% = 8.45; N% = 2.55; obtd.: C% = 65.65; H% = 8.42; N% = 2.57.

**12α-bromo-3-thioxo-3a-aza-A-homoolean-28-oic acid 28β→13β-olide** (**14**): To a saturated boiled solution of 547 mg (1.0 mmol) of 12α-bromo-3-oxo-3a-aza-A-homoolean-28-oic acid 28β→13β-olide (**13**) in benzene, 404 mg (1.0 mmol) of Lavesson’ reagent was added and the mixture was refluxed until the reaction was completed (TLC control). The post-reaction mixture was cooled, then washed with a 5% solution of K_2_CO_3_ and then with water. Next, the mixture was dried with MgSO_4_ and subjected to column chromatography on silica gel, applying CHCl_3_ as an eluent.

**R_f_**: 0.86 (1:1); 0.81 (2:1); 0.69 (4:1); 0.41 (9:1); 0.39 (CHCl_3_); 0.26 (15:1); 0.11 25:1); yellow-brown spot. **Yield**: 511 mg (90.8%). **M.p.**: 253–256 °C (EtOH, decomp.). **^1^H NMR** (δ): 7.81 (1H, s, N-H, thiolactam); 4.29 (1H, t, *J* = 2.9 Hz, C_12_-H_β_); 2.33 (1H, t, *J* = 4.9 Hz; C_18_-H_β_); 1.43, 1.41, 1.30, 1.24, 1.09, 0.98, 0.89 (7 × 3H, 7 × s, 7 × CH_3_ group). **^13^C NMR** (δ): 206.5 (C_q_, C-3); 178.6 (C_q_, C-28); 91.5 (C_q_, C-13); 56.2 (C_q_, C-12); 45.5 (C_q_, C-17). **DEPT**: 7 × CH_3_, 10 × CH_2_, 4 × CH; 30 × C atom. **HR MS**: for M^+•^ ion of composition C_30_H_46_BrNO_2_S calcd.: 563.2433, obtd.: 563.2435. **Elem. anal.**: calcd.: C% = 63.81; H% = 8.21; N% = 2.48; obtd.: C% = 63.82; H% = 8.23; N% = 2.46.

The graphical form of ^1^H NMR and ^13^C NMR spectra of oleanolic acid and its lactones **2**–**8** and bromolactones **9**–**14** are given in the Supplementary Materials, File S4.

#### 4.2.5. SAR Analysis of Oleanolic Acid Lactones **2**–**8** and Bromolactones **9**–**14**

The PASS (Prediction of Activity Spectra for Substance) computer system [71] predicts many types of pharmacological activity and mechanisms of actions, based on the structure of a compound, using for this purpose the appropriate MNA (Multilevel Neighborhoods of Atoms) descriptors. The essential elements of this program are a database (Training Set), structure descriptors (Chemical Structure Descriptors), Biological Activity Descriptors, and Mathematical Approach [72,73].

As a result of the mathematical analysis, the prediction result is obtained as a list of found types of activities. The probability of occurrence of a given activity is defined as P_a_, and the probability of lack of this activity is defined as P_i_. Both values are expressed in a range between 0.000 and 1.000.

#### 4.2.6. Cytotoxic Properties of Oleanolic Acid Lactones **2**–**8** and Bromolactones **9**–**14**

The cell lines were cultured as follows: CHO (*Chinese hamster ovary*) cells were grown in the RMPI 1640 medium; A-549 (*human lung carcinoma*) cells were cultured in the RPMI 1640 medium; PC-3 (*human prostate carcinoma*) cells were cultured in the F-12K medium; SKOV-3 (*human ovarian carcinoma*) cells were cultured in the McCoy’s Modified Medium; and HDFs (*human normal fibroblasts*) were cultured in the Fibroblast Basal Medium. Each medium was supplemented with 10% fetal bovine serum, 1% L-glutamine, and 1% penicillin/streptomycin solution. The cell lines were incubated at 37 °C in a humidified atmosphere containing 5% CO_2_. Cells were counted using the Fuchs–Rosenthal counting chamber (hemocytometer) with modifications.

In short, the MTT assay is based on the reaction between the mitochondria enzymes (dehydrogenases) of tested cell lines and 3-(4,5-dimethylthiazol-2-yl)-2,5-diphenyltetrazolium bromide (MTT). The final product of this reaction gives violet formazan crystals. The number of live cells is proportional to the amount of formazan and is represented by the violet dye reagent. The details of the experiments are given, e.g., in [54].

#### 4.2.7. Apoptotic Activity of Oleanolic Acid Lactones **2**–**8** and Bromolactones **9**–**14**

The Cell Death Detection ELISA PLUS photometric enzyme immunoassay is used to quantitatively in vitro determination of cytoplasmic histone-associated DNA fragments (mono- and oligonucleosomes) after induced cell death. The kit contains a stop solution that allows users to terminate the substrate reaction and run the assay under defined conditions, making it suitable for high-throughput applications. This kit can be used for the relative quantification of histone-complexed DNA fragments (mono- and oligonucleosomes) out of the cytoplasm of cells after the induction of apoptosis or when released from necrotic cells. Since the assay does not require the labelling of cells, it can detect internucleosomal degradation of genomic DNA during apoptosis even in cells that do not proliferate in vitro; for example, freshly isolated tumour cells. The antibodies used in the assay are not species-specific; therefore, the kit may be used to assay cells from various species. The details of the experiments are given in [74]. As an internal reference compound (cytostatic drug), camptothecin (topoisomerase I-inhibitor) was used in this study to induce apoptosis. The negative control is the sample without camptothecin treatment. Cells were treated with three different concentrations of each compound (0.1, 1.0, and 10.0 μg/mL) and DNA fragmentation as an indicator for apoptosis was determined using a cell death ELISA Plus Assay. Cells were counted using a Fuchs–Rosenthal counting chamber (hemocytometer) with modifications. The apoptotic index was calculated based on the enrichment of mono- and oligonucleosomes according to the following equation:

apoptotic index = [{A_405(sample)_ − A_490(sample)_}/{A_405(negative control)_ − A_490(negative control)_}]

#### 4.2.8. Statistical Analysis

Statistical analyses for the cytotoxic activity were carried out using the nonparametric Kruskal–Wallis H test for unpaired data and ANOVA Friedman two-way analysis of variance test for paired data. Statistical significance was tested using Dunn’s post-hoc test. The IC_50_ values compared to the control IC_50_ were calculated using GraphPad Prism v. 10.1.1. software.

#### 4.2.9. Molecular Docking for Oleanolic Acid Lactones **2**–**8** and Bromolactones **9**–**14**

**Ligand Preparation**: The preparation of ligands began with constructing the two-dimensional (2D) structures of compounds **1**–**14** using ChemDraw 22.0.0. These 2D structures were converted into three-dimensional (3D) representations using OpenBabel [75], ensuring the generation of coordinates corresponding to the most energetically favourable conformations. Geometry optimisation was conducted with Avogadro version 1.2.0, employing the Universal Force Field (UFF) and the Steepest Descent algorithm. The optimised 3D structures of the compounds were saved in SDF format and later utilised as input files for docking studies on the CB-Dock2 platform.

**Protein Preparation**: The crystallographic data for the EGFR protein (Epidermal Growth Factor Receptor tyrosine kinase domain) with the 1M17 structure were obtained from the RCSB Protein Data Bank (PDB ID: 1M17) at a resolution of 2.60 Å. In this X-ray crystal structure, EGFR is co-crystallised with the inhibitor erlotinib. The downloaded 1M17.pdb protein molecule was not manually prepared, as processes such as removing ligands, crystal water molecules, and adding missing hydrogen atoms were automatically performed by the CB-Dock2 server.

**Detecting Cavities and Uploading Ligands**: After importing the EGFR molecule (PDB ID: 1M17) into the CB-Dock2 server, the number of cavities for docking was set to 5 under the “more parameters” option. The email address for receiving the output files was provided, and the “Search Cavities” function was selected to detect potential binding sites. Subsequently, the ligands (compounds **1**–**14**) and the EGFR protein were uploaded to the server, with the number of cavities again set to 5 in the “more parameters” option. After filling in the email address, the “Auto Blind Docking” button was selected to initiate the docking process.

#### 4.2.10. Antioxidant Activity of Oleanolic Acid Lactones **2**–**8** and Bromolactones **9**–**14**

The CUPRAC method measures the reducing power of a sample by quantifying its ability to convert the Cu-neocuproine reagent complex into the Cu(I) form. The DPPH assay relies on the ability of an antioxidant to donate a hydrogen atom or an electron to the stable DPPH radical, resulting in its reduction and subsequent colour change. In both methods, the results are presented as % inhibition of the copper(II) ions and Trolox equivalent calculated from the standard curve and % inhibition of the DPPH radical and Trolox equivalent, calculated from the standard curve, respectively. The spectroscopic detection in CUPRAC and DPPH assays were conducted with Synergy H1 Multi-Mode Microplate Reader (BioTek Instruments, Inc., Winooski, VT, USA). The details of the experiments are given in [42].

#### 4.2.11. ADMETox Analysis of Oleanolic Acid Lactones **2**–**8** and Bromolactones **9**–**14**

The physicochemical properties, pharmacokinetics, and ADMETox (adsorption, distribution, metabolisms, excretion, and toxicity) activity of oleanolic acid lactones **2**–**8** (Figure 2) and bromolactones **9**–**14** (Figure 3) were estimated based on the comprehensive database ADMETlab Manual (2.0) [61]. First, the structures of the analysed compounds were prepared using the JSME editor.

## 5. Conclusions

This study demonstrated the potential of oleanolic acid derivatives, particularly lactones and bromolactones, as effective anticancer agents. Compounds **6**, **2** (Figure 2), and **10** (Figure 3) exhibited potent cytotoxic activity, with IC_50_ values significantly lower than the parent oleanolic acid, indicating enhanced efficacy. Molecular docking revealed robust interactions with the EGFR tyrosine kinase domain, specifically in pockets C1 and C2, where key residues like LYS 721 and ASP 831 contributed to stable ligand binding. The SAR analysis highlighted the importance of structural modifications, such as the hydroxyimino and bromolactone moieties, in enhancing biological activity.

The antioxidant assays showed that the derivatives possess diverse mechanisms of action, with compounds **7** (Figure 2) and **10** (Figure 3) excelling in electron transfer and radical scavenging, respectively. ADMETox evaluations confirmed that most derivatives exhibit favourable pharmacokinetic and safety profiles, supporting their potential as drug candidates. While compounds **6**, **2** (Figure 2), and **10** (Figure 3) stand out as the most promising, others like **9** and **12** (Figure 3) may also warrant further investigation due to their unique interaction profiles and structural features. These findings offer a strong foundation for developing triterpene-based therapies targeting EGFR-associated cancers.

To sum up the results of the performed research, the observations made and the conclusions drawn, the following can be stated:

**Key findings and their implications**—potential for new drug development: We identified three compounds (**2**, **6**, **10**; Figure 2 and Figure 3) that exhibited significant cytotoxic effects against the A-549, PC-3, and SKOV-3 cancer cell lines, with favourable selectivity indices (SI > 2). In particular, compound **6** (3-acetoxy-12-hydroxyiminolactone) showed an IC_50_ of 1.27 µM and strong binding with key EGFR residues (LYS721 and ASP831). Such high activity and consistency between in vitro and in silico results confirm its potential as a lead structure for further preclinical development.

**Comparison with existing research**: The obtained results are consistent with earlier reports on triterpene oximes (e.g., [39,54]). Our study additionally demonstrated that the presence of an acetoxy group at position C-3 enhances the activity of the hydroxyimino group at C-12, which was not previously directly shown. A novelty is also the observation of the high activity of the A-ring nitrile (compound **12**), previously considered biologically less relevant.

**Limitations and strengths:**

- Limitations:
  ➢ Some compounds (e.g., **7**, **8**, and **13**) showed poor solubility and limited biological activity;
  ➢ Despite using an advanced CB-Dock2 algorithm, molecular docking does not fully account for receptor flexibility.
- Strengths:
  ➢ A high correlation between docking results and biological testing;
  ➢ Comprehensive assessment of activities: cytotoxicity, pro-apoptotic potential, antioxidant effects, and ADMETox profile;
  ➢ Data provide a basis for designing next-generation semisynthetic triterpenes.

**Suggestions for future research**: We plan further structural modifications:

- Changes at position C-12 (e.g., introducing Zn^2+^ chelators);
- Adding polar fragments to enhance bioavailability;
- Testing additional cancer cell lines and conducting in vivo pharmacokinetic analyses;
- Molecular dynamics (MD) simulations are planned to better understand ligand–receptor complex stability.

## Figures and Tables

**Table 1:** The predicted activity of oleanolic acid (**1**) and lactones **2**–**8** determined by the PASS method.

1.000–0.950	0.949–0.900	0.899–0.850	0.849–0.800	0.799–0.750	0.749–0.700	<0.700

Activity	P_a_ Factor (and P_i_ Factor) of Compounds 1–8 (P_a_ ≥ 0.700)							
	1 (OA)	2	3	4	5	6	7	8
**Acylcarnitine hydrolase inhibitor**	≤0.700	0.731 (0.022)	0.779 (0.016)	≤0.700	≤0.700	≤0.700	≤0.700	≤0.700
**Antileukemic**	≤0.700	≤0.700	≤0.700	≤0.700	≤0.700	≤0.700	0.755 (0.005)	≤0.700
**Antineoplastic**	0.876 (0.005)	0.934 (0.004)	0.928 (0.005)	0.951 (0.004)	0.940 (0.004)	0.947 (0.004)	0.951 (0.004)	0.939 (0.004)
**Antineoplastic** **(colon cancer)**	≤0.700	≤0.700	≤0.700	≤0.700	≤0.700	0.841 (0.004)	≤0.700	0.801 (0.004)
**Antineoplastic (colourectal cancer)**	≤0.700	≤0.700	≤0.700	≤0.700	≤0.700	0.843 (0.004)	≤0.700	0.803 (0.004)
**Antineoplastic** **(lung cancer)**	0.766 (0.005)	0.863 (0.003)	0.855 (0.004)	0.848 (0.004)	0.825 (0.004)	0.808 (0.004)	0.815 (0.004)	0.775 (0.004)
**Antinociceptive**	≤0.700	≤0.700	0.710 (0.003)	≤0.700	≤0.700	≤0.700	≤0.700	≤0.700
**Apoptosis agonist**	0.901 (0.004)	0.879 (0.005)	0.889 (0.004)	0.898 (0.004)	0.898 (0.004)	0.881 (0.005)	0.921 (0.004)	0.884 (0.005)
**Caspase 3 stimulant**	0.984 (0.002)	0.882 (0.004)	0.923 (0.003)	0.905 (0.003)	0.962 (0.003)	0.920 (0.003)	0.764 (0.008)	0.969 (0.002)
**Caspase 8 stimulant**	0.914 (0.001)	0.835 (0.001)	0.830 (0.001)	0.846 (0.001)	0.854 (0.001)	0.724 (0.003)	0.747 (0.003)	0.735 (0.003)
**Chemopreventive**	0.937 (0.002)	0.742 (0.005)	≤0.700	≤0.700	≤0.700	≤0.700	≤0.700	≤0.700
**CYP2J substrate**	0.808 (0.019)	≤0.700	0.734 (0.039)	≤0.700	0.774 (0.027)	≤0.700	≤0.700	≤0.700
**Hepatic disorders treatment**	0.834 (0.004)	≤0.700	≤0.700	≤0.700	≤0.700	≤0.700	≤0.700	≤0.700
**Hepatoprotectant**	0.930 (0.002)	0.755 (0.005)	0.705 (0.007)	≤0.700	≤0.700	≤0.700	≤0.700	≤0.700
**Hypolipemic**	≤0.700	0.805 (0.007)	0.757 (0.010)	0.783 (0.008)	0.774 (0.009)	0.753 (0.010)	≤0.700	0.732 (0.011)
**Insulin promotor**	0.869 (0.004)	≤0.700	0.760 (0.004)	≤0.700	0.753 (0.004)	≤0.700	≤0.700	≤0.700
**Membrane integrity antagonist**	0.928 (0.002)	0.776 (0.009)	≤0.700	0.704 (0.014)	≤0.700	≤0.700	≤0.700	≤0.700
**Mucomembranous protector**	0.894 (0.005)	0.727 (0.045)	≤0.700	0.742 (0.038)	≤0.700	≤0.700	≤0.700	≤0.700
**Nitric oxide antagonist**	≤0.700	0.752 (0.003)	0.775 (0.003)	0.781 (0.003)	0.777 (0.003)	0.701 (0.003)	0.823 (0.002)	0.702 (0.003)
**Oxidoreductase inhibitor**	0.904 (0.002)	0.774 (0.008)	0.714 (0.014)	0.801 (0.006)	0.773 (0.008)	≤0.700	≤0.700	≤0.700
**Phosphatase inhibitor**	0.894 (0.001)	0.711 (0.011)	0.756 (0.005)	0.712 (0.011)	0.761 (0.005)	≤0.700	≤0.700	0.729 (0.008)
**T-17β-dehydratase (NADP+) inhibitor**	0.892 (0.007)	≤0.700	0.746 (0.040)	≤0.700	0.789 (0.028)	≤0.700	≤0.700	≤0.700
**Transcription factor NF kappa B stimulant**	0.954 (0.001)	0.817 (0.002)	0.878 (0.002)	0.827 (0.002)	0.876 (0.002)	0.784 (0.003)	0.881 (0.002)	0.844 (0.002)
**Transcription factor stimulant**	0.954 (0.001)	0.817 (0.002)	0.878 (0.002)	0.827 (0.002)	0.876 (0.002)	0.784 (0.003)	0.881 (0.002)	0.844 (0.002)

**Legend**: **OA** = oleanolic acid (reference compound); **P_a_** = probability of activity; **P_i_** = probability of inactivity; yellow area = lactones.

**Table 2:** The predicted activity of bromolactones **9**–**14** determined by the PASS method.

1.000–0.950	0.949–0.900	0.899–0.850	0.849–0.800	0.799–0.750	0.749–0.700	<0.700

Activity	P_a_ Factor (and P_i_ Factor) of Compounds 9–14 (P_a_ ≥ 0.700)					
	9	10	11	12	13	14
**Acylcarnitine hydrolase inhibitor**	≤0.700	≤0.700	≤0.700	≤0.700	≤0.700	≤0.700
**Antileukemic**	0.707 (0.005)	0.784 (0.004)	≤0.700	≤0.700	≤0.700	≤0.700
**Antineoplastic**	0.904 (0.005)	0.916 (0.005)	0.871 (0.005)	0.860 (0.006)	0.785 (0.014)	0.797 (0.012)
**Antineoplastic** **(colon cancer)**	≤0.700	≤0.700	≤0.700	≤0.700	≤0.700	≤0.700
**Antineoplastic** **(colourectal cancer)**	≤0.700	≤0.700	≤0.700	≤0.700	≤0.700	≤0.700
**Antineoplastic** **(lung cancer)**	0.769 (0.005)	0.738 (0.005)	≤0.700	0.746 (0.005)	≤0.700	≤0.700
**Antinociceptive**	≤0.700	≤0.700	≤0.700	≤0.700	≤0.700	≤0.700
**Apoptosis agonist**	0.872 (0.005)	0.895 (0.004)	0.825 (0.006)	0.724 (0.013)	≤0.700	≤0.700
**Caspase 3 stimulant**	0.811 (0.005)	≤0.700	≤0.700	≤0.700	≤0.700	≤0.700
**Caspase 8 stimulant**	0.777 (0.002)	≤0.700	≤0.700	≤0.700	≤0.700	≤0.700
**Chemopreventive**	≤0.700	≤0.700	≤0.700	≤0.700	≤0.700	≤0.700
**CYP2J substrate**	≤0.700	≤0.700	≤0.700	≤0.700	≤0.700	≤0.700
**Hepatic disorders treatment**	≤0.700	≤0.700	≤0.700	≤0.700	≤0.700	≤0.700
**Hepatoprotectant**	≤0.700	≤0.700	≤0.700	≤0.700	≤0.700	≤0.700
**Insulin promotor**	≤0.700	≤0.700	≤0.700	≤0.700	≤0.700	≤0.700
**Interleukin 2 agonist**	≤0.700	≤0.700	≤0.700	≤0.700	≤0.700	0.732 (0.002)
**Membrane integrity antagonist**	≤0.700	≤0.700	≤0.700	≤0.700	≤0.700	≤0.700
**Mucomembranous protector**	≤0.700	≤0.700	≤0.700	≤0.700	≤0.700	≤0.700
**Nitric oxide antagonist**	0.706 (0.003)	0.762 (0.003)	≤0.700	≤0.700	≤0.700	≤0.700
**Oxidoreductase inhibitor**	≤0.700	≤0.700	≤0.700	≤0.700	≤0.700	≤0.700
**Phosphatase inhibitor**	0.740 (0.007)	≤0.700	≤0.700	≤0.700	≤0.700	≤0.700
**T-17β-dehydratase (NADP+) inhibitor**	≤0.700	≤0.700	≤0.700	≤0.700	≤0.700	≤0.700
**Transcription factor NF kappa B stimulant**	0.857 (0.002)	0.849 (0.002)	0.747 (0.003)	0.786 (0.003)	≤0.700	≤0.700
**Transcription factor stimulant**	0.857 (0.002)	0.849 (0.002)	0.747(0.003)	0.786 (0.003)	≤0.700	≤0.700

**Legend**: **OA** = oleanolic acid (reference compound); **P_a_** = probability of activity; **P_i_** = probability of inactivity; green area = bromolactones.

**Table 3:** Results of MTT assay for oleanolic acid (**1**), lactones **2**–**8**, and bromolactones **9**–**14**.

≤5.00 μM	5.01–10.00 μM	10.01–20.00 μM	20.01–50.00 μM	≥50.01 μM

Comp. No.	IC_50_ [μM] (±s)				
	CHO	A-549	PC-3	SKOV-3	HDFs
**1**	9.16 (0.16)	8.79 (±0.20) *	18.63 (±0.05) **	18.81 (±0.09) **	24.87 (±0.04) **
**2**	4.19 (0.03)	4.68 (0.24)	3.26 (0.06)	2.96 (0.01)	7.51 (0.09)
**3**	46.96 (0.17)	23.27 (0.15)	21.88 (0.08)	21.09 (0.17)	38.27 (0.19)
**4**	52.66 (0.18)	24.67 (0.16)	25.91 (0.16)	22.81 (0.15)	41.95 (0.12)
**5**	38.24 (0.17)	38.03 (0.17)	31.02 (0.07)	36.99 (0.02)	51.84 (0.11)
**6**	5.49 (0.15)	1.27 (0.01)	1.88 (0.01)	1.73 (0.04)	3.91 (0.04)
**7**	>100	66.14 (0.17)	>100	>100	>100
**8**	>100	>100	>100	>100	>100
**9**	42.01 (0.15)	29.87 (0.15)	26.09 (0.03)	26.41 (0.13)	51.03 (0.19)
**10**	21.36 (0.11)	15.55 (0.21)	12.94 (0.06)	12.05 (0.11)	39.11 (0.11)
**11**	55.41 (0.13)	34.63 (0.12)	25.13 (0.11)	21.75 (0.09)	28.94 (0.15)
**12**	9.23 (0.04)	7.88 (0.11)	6.27 (0.03)	6.22 (0.01)	9.13 (0.06)
**13**	>100	89.32 (0.24)	73.09 (0.18)	75.83 (0.19)	89.37 (0.16)
**14**	86.95 (0.22)	66.23 (0.24)	65.88 (0.12)	68.31 (0.18)	84.09 (0.11)

**Legend**: **IC_50_** = half maximal inhibitory concentration; **±s** = standard deviation; **A-549** = *human lung carcinoma*; **CHO** = *Chinese hamster ovary*; **PC-3** = *human prostate carcinoma*; **SKOV-3** = *human ovarian carcinoma*; **HDF** = *human dermal fibroblasts*; * = published in [40]; ** = published in [41]; yellow area = lactones; green area = bromolactones.

**Table 5:** Apoptotic Index for oleanolic acid (**1**) and the most active lactones (**2** and **6**) and bromolactones (**10** and **12**) determined in the Elisa Plus assay.

7.00–7.99	6.00–6.99	5.00–5.00	4.00–4.99

Comp. No.	Cell Line, AI	
	PC-3	SKOV-3
**1**	5.27 (0.04)	5.16 (0.01)
**2**	4.76 (0.02)	4.71 (0.11)
**6**	7.28 (0.02)	7.22 (0.06)
**10**	5.82 (0.04)	5.05 (0.03)
**12**	5.43 (0.05)	5.11 (0.01)

**Legend: AI** = Apoptotic Index; **PC-3** = *human prostate carcinoma*; **SKOV-3** = *human ovarian carcinoma*; yellow area = lactones; green area = bromolactones.

**Table 6:** Division of protein pockets by volume and docking results from the CB-Dock2 web server.

CurPocket ID	Cavity Volume (Å^3^)	Centre (x, y, z)	Cavity Size (x, y, z)	Comments
**C1**	991	36, 8, 51	14, 17, 13	The largest pocket, a typical EGFR active site
**C2**	665	24, 0, 54	15, 12, 9	Strong bonds, particularly for bromolactones
**C3**	416	31, −4, 40	10, 14, 7	Smaller pocket, less favourable results
**C4**	184	40, 18, 67	8, 9, 5	A small cavity with limited bonding possibilities
**C5**	173	3, 15, 63	6, 10, 7	Small pocket, hydrophobic interactions

## Data Availability

All data concerning this paper are available in the manuscript body or in the Supplementary Data.

## Supplementary Materials

The following supporting information can be downloaded at: https://www.mdpi.com/article/10.3390/ijms26094099/s1. Ref. [76] is cited in Supplementary Materials file.

## Abbreviations

The following abbreviations are used in this manuscript:

±s	standard deviation
-OH	hydroxyl group
=NOH	hydroxyimine group
µg	microgram = 10^−6^ g
µg/mL	microgram per milliliter
µM	micromole = 10^−6^ moles
δ	chemical shift
^1^H NMR	proton nuclear magnetic resonance
1H, 2H, etc.	intensity of 1H NMR signal (intensity of 1 proton, 2 protons, etc.)
^13^C NMR	carbon nuclear magnetic resonance
2D	two-dimensional
3D	three-dimensional
AcOEt	ethyl acetate
ADMETox	Adsorption, Distribution, Metabolisms, Excretion, and Toxicity
AI	Apoptotic Index
ATTC	American Type Culture Collection
Å	angstrom
Å^3^	cubic angstrom
BMS	alert for mapping molecular promiscuity and identification of undesirable and reactive compounds
C-3, C-13, etc.	carbon atom at the 3, 12, etc. position of oleanane skeleton
C_3_-H, C_12_-H, etc.	hydrogen atom attached to the C-3, C-12, etc. atom of oleanane skeleton
C_18_-H_β_	hydrogen atom at β orientation attached to the C-18 atom of oleanane skeleton
C_6_H_6_	benzene
calcd.	calculated
CDCl_3_	deutherated chloroform
CH	tertiary carbon atom
CH_2_	secondary carbon atom
CH_3_	primary carbon atom
Comp.	compound
C_q_	quaternary carbon atom
CUPRAC	Cupric Ion Reducing Antioxidant Capacity
CYP	cytochrome P
dd	doublet of doublets
decomp.	decomposition
DMSO-*d*_6_	deutherated dimethylsulfoxide
DNA	deoxyribonucleic acid
DPPH	2,2-diphenyl-1-picrylhydrazyl hydrate
DPPH^·^	2,2-diphenyl-1-picrylhydrazyl hydrate radical
Elem. Anal.	Elemental analysis
ELISA	enzyme-linked immunosorbent assay; a commonly used laboratory test to detect antibodies in the blood
EMA	European Medicines Agency
EtOH	ethanol
F-12K	Kaighn’s Modification of Ham’s F-12 Medium
fChar	formal charge
FDA	Food and Drug Administration
HP TLC	High Performance Liquid Chromatography
HR MS	High Resolution Mass Spectrometry
Hz	Hertz
IC_50_	half maximal inhibitory concentration
*J*	coupling constant
JSME	Japan Society of Mechanical Engineers
kcal/mol	a unit to measure an amount of energy per number of molecules/atoms or other perticles
LogD	logP at physiological pH 7.4
lit.	literature, according to literature data
logP	logaritm of the octanol/water partition coefficient
logS	logaritm of the aqueous solubility
*m*-CPBA	*m*-chloroperbenzoic acid
MHz	megahertz = 10^6^ Hertzes
mmol	milimol = 10^−3^ moles
MMPs	matrix metalloproteinases
MNA	Multilevel Neighborhoods of Atoms
M.p.	melting point
MTT	3-(4,5-dimethylthiazol-2-yl)-2,5-diphenyltetrazolium bromide
NF-κB	nuclear-kappa B
nHA	number of hydrogen bond acceptors
nHD	number of hydrogen bond donors
nHet	number of heteroatoms
No.	number
NP	Natural Product-likeness
nRig	number of rigid bonds
nRing	number of rings
nRot	number of rotatable bonds
OA	oleanolic acid
obtd.	obtained
P_a_	probability of activity
PAINS	Pan Assay Interference Compounds
PASS	Prediction of Activity Spectra for Substances
Pgp	p-glycoprotein
P_i_	probability of inactivity
ppm	part per million
precipt.	precipitated
R_f_	retension factor
RMPI 1640	Roswell Park Memorial Institute 1640 medium
RNS	Reactive Nitrogen Species
ROS	Reactive Oxygen Species
r.t.	room temperature
s	singlet
SAR	Structure—Activity Relationship
SI	Selectivity Index
t	triplet
TLC	Thin Liquid Chromatography
TMS	tetramethylsilane
UFF	Universal Force Field
VIS	visible light
VIS	visible light
Zn^2+^	divalent zinc cation
