# Supplementary material for: Oleanolic Acid Lactones as Effective Agents in the Combat with Cancers—Cytotoxic and Antioxidant Activity, SAR Analysis, Molecular Docking and ADMETox Profile
Source: Int J Mol Sci. 2025 Apr 25;26(9):4099. doi: 10.3390/ijms26094099 (PMC12072072; doi:10.3390/ijms26094099)
Supplement: Supplementary file 1 [file ijms-26-04099-s001.zip › Suppl. Mat. File S1. Standard curves.pdf]

International Journal of
Molecular Sciences

Supplementary Materials. File S1

Figure S1. Standard curve for (**A**) CUPRAC and (**B**) DPPH assays as Trolox equivalent.

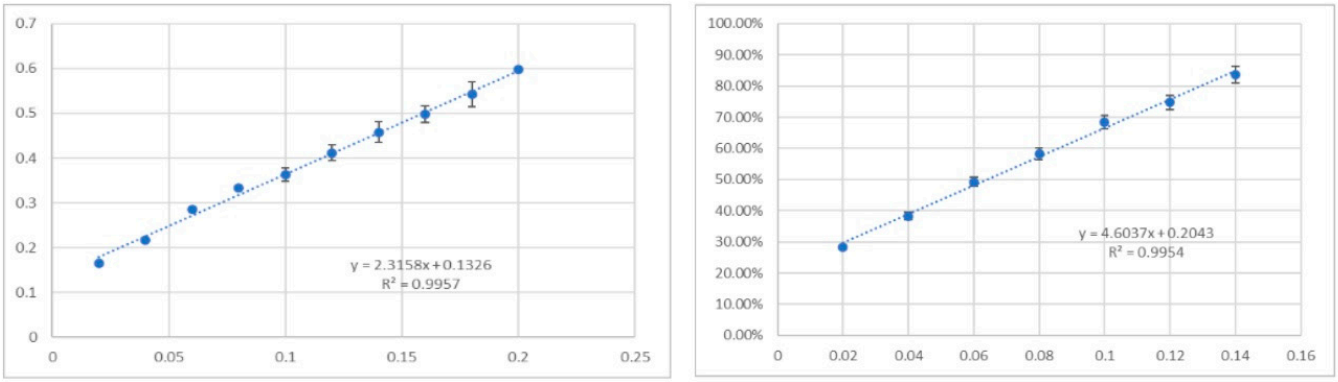

A **B**
