# Supplementary material for: Oleanolic Acid Lactones as Effective Agents in the Combat with Cancers—Cytotoxic and Antioxidant Activity, SAR Analysis, Molecular Docking and ADMETox Profile
Source: Int J Mol Sci. 2025 Apr 25;26(9):4099. doi: 10.3390/ijms26094099 (PMC12072072; doi:10.3390/ijms26094099)
Supplement: Supplementary file 1 [file ijms-26-04099-s001.zip › Suppl. Mat. File S2. ADMETox data.pdf]

International Journal of *Molecular Sciences*

MDPI

Supplementary Materials. File S2

Table S1. ADMETox data for oleanolic acid (**1**) and its derivatives **2** – **8**

High (optimal) value	Moderate value	Low value	Neutral (informative) value

For values given in a range 0.000 – 1.000 (unless stated otherwise): 0.000 – 0.300: low probability, 0.301 – 0.699: moderate probability, 0.700 – 1.000: high probability

Physicochemical Properties (optimal values)	Compound number							
	**1**	**2**	**3**	**4**	**5**	**6**	**7**	**8**
Molecular Weight (100 ~ 600)	456.360	514.370	472.360	512.350	470.340	527.360	468.320	485.350
Volume	505.750	549.367	508.662	546.731	505.985	557.728	503.349	516.982
Density	0.902	0.936	0.929	0.937	0.930	0.946	0.930	0.939
NHA (0 ~ 12)	3	5	4	5	4	6	4	5
nHD (0 ~ 7)	2	1	2	0	1	1	0	2
nRot (0 ~ 11)	1	2	0	2	0	2	0	0
nRing (0 ~ 6)	5	6	6	6	6	6	6	6
maxRing (0 ~ 18)	22	19	19	19	19	19	19	19
nHet (1 ~ 15)	3	5	4	5	4	6	4	5
fChar (-4 ~ +4)	0	0	0	0	0	0	0	0
nRig (0 ~ 30)	27	31	30	32	31	32	32	31
Flexibility (≤ 2)	0.037	0.065	0.000	0.062	0.000	0.062	0.000	0.000
Stereo Centers (≤ 2)	8	10	10	9	9	9	8	9
TPSA (0 ~ 140)	57.530	72.830	66.76	69.670	63.600	85.190	60.440	79.120
LogS (-4 ~ 0.5)	-5.036	-5.583	-4.994	-5.788	-5.408	-5.655	-5.407	-5.306
LogP (0 ~ 3)	6.113	5.462	4.844	5.697	5.089	5.008	4.657	4.445
LogD (1 ~ 3)	4.843	4.441	4.312	4.616	4.474	3.908	4.280	3.813
QED (≥ 0.670)	0.409	0.407	0.432	0.368	0.435	0.227	0.392	0.238

https://doi.org/10.3390/xxxxx

Sascore (≤ 6)	4.589	6.219	6.214	6.226	6.226	6.372	6.197	6.378
Fsp3 (≥ 0.420)	0.900	0.938	0.967	0.906	0.933	0.906	0.900	0.933
MCE-18 (≥ 45)	105.368	170.645	168.559	169.672	167.586	169.672	166.579	167.586
Npscore (-5 ~ 5)	3.272	3.194	3.120	2.980	3.051	2.730	2.980	2.752
Lipinski Rule	A	R	A	R	A	R	A	A
Pfizer Rule	R	R	R	R	R	A	R	A
GSK Rule	R	R	R	R	R	R	R	R
Golden Triangle	A	R	A	R	A	R	A	A
PAINS (alerts)	0	0	0	0	0	0	0	0
ALARM NMR (alerts)	0	1	1	1	1	1	1	1
BMS (alerts)	0	0	0	0	0	1	0	1
Chelator Rule (alerts)	0	0	0	0	0	0	0	0
Caco-2 Permeability (≥ -5.15)	-5.198	-5.086	-5.095	-5.086	-5.146	-5.166	-5.217	-5.260
MDCK Permeability (≤ 2 × 10^{-6} cm/s)	2.00 x 10^{-5}	1.80 x 10^{-5}	1.76 x 10^{-5}	1.81 x 10^{-5}	1.66 x 10^{-5}	2.27 x 10^{-5}	1.59 x 10^{-5}	2.35 x 10^{-5}
Pgp-inhibitor (≤ 0.300)	0.000	0.990	0.910	0.960	0.705	0.969	0.428	0.797
Pgp-substrate (≤ 0.300)	0.000	0.000	0.001	0.000	0.000	0.000	0.000	0.000
HIA (≤ 0.300)	0.012	0.012	0.015	0.028	0.033	0.035	0.126	0.044
$F_{20\%}$ (≤ 0.300)	0.074	0.662	0.880	0.934	0.892	0.962	0.555	0.908
$F_{30\%}$ (≤ 0.300)	0.756	0.899	0.934	0.960	0.983	0.968	0.992	0.970
PPB (≤ 90%)	98.130	93.081	94.030	90.802	92.391	95.195	90.599	95.860
VD (0.04 – 20 L/kg)	0.718	0.990	0.921	0.826	0.842	1.046	0.902	1.086
BBB Penetration (≤ 0.300)	0.674	0.424	0.607	0.857	0.922	0.327	0.980	0.583
Fu (≥ 5.000 %)	3.524	3.655	4.625	4.485	5.482	3.050	5.642	3.598
CYP1A2 inhibitor (≥ 0.700)	0.012	0.012	0.013	0.013	0.015	0.014	0.018	0.016
CYP1A2 substrate (≥ 0.700)	0.323	0.157	0.328	0.281	0.533	0.247	0.768	0.512
CYP2C19 inhibitor (≥ 0.700)	0.028	0.055	0.045	0.077	0.065	0.129	0.116	0.090
CYP2C19 substrate (≥ 0.700)	0.916	0.880	0.892	0.929	0.933	0.542	0.952	0.609
CYP2C9 inhibitor (≥ 0.700)	0.157	0.242	0.172	0.310	0.263	0.629	0.265	0.477
CYP2C9 substrate (≥ 0.700)	0.813	0.091	0.090	0.078	0.079	0.078	0.260	0.076
CYP2D6 inhibitor (≥ 0.700)	0.012	0.009	0.009	0.009	0.011	0.015	0.017	0.014
CYP2D6 substrate (≥ 0.700)	0.528	0.162	0.249	0.099	0.132	0.067	0.172	0.070
CYP3A4 inhibitor (≥ 0.700)	0.172	0.598	0.620	0.681	0.684	0.785	0.769	0.806
CYP3A4 substrate (≥ 0.700)	0.208	0.567	0.372	0.811	0.656	0.761	0.770	0.615

CL (≥15 mL/min/kg)	3.094	7.422	12.411	7.697	13.356	1.876	11.414	4.997
$T_{1/2\ [<3h]}$ (≤ 0.300)	0.023	0.011	0.021	0.012	0.025	0.009	0.103	0.015
hERG Blockers (≤ 0.300)	0.004	0.530	0.164	0.089	0.044	0.158	0.020	0.056
H-HT (≤ 0.300)	0.296	0.582	0.602	0.399	0.458	0.512	0.421	0.553
DILI (≤ 0.300)	0.010	0.029	0.011	0.153	0.025	0.048	0.050	0.015
AMESToxicity (≤ 0.300)	0.008	0.007	0.006	0.015	0.011	0.005	0.018	0.005
Rat Oral Acute Toxicity (≤ 0.300)	0.228	0.605	0.927	0.487	0.964	0.097	0.749	0.767
FDAMDD (≤ 0.300)	0.909	0.883	0.948	0.784	0.921	0.849	0.488	0.929
Skin Sensitization (≤ 0.300)	0.028	0.136	0.133	0.066	0.091	0.043	0.031	0.053
Carcinogencity (≤ 0.300)	0.063	0.231	0.351	0.280	0.397	0.272	0.673	0.392
Eye Corrosion (≤ 0.300)	0.012	0.007	0006	0.012	0.011	0.004	0.008	0.004
Eye Irritation (≤ 0.300)	0.084	0.012	0.013	0.031	0.038	0.023	0.085	0.029
Respiratory Toxicity (≤ 0.300)	0.968	0.965	0.973	0.958	0.966	0.945	0.959	0.956
Bioconcentration Factors	1.944	2.241	2.145	2.203	1.803	2.771	1.033	2.335
IGC_{50}	5.021	5.053	4.970	4.984	4.876	5.010	4.443	4.909
$LC_{50}FM$	5.937	5.936	5.678	5.964	5.704	5.911	4.968	5.651
$LC_{50}DM$	6.337	6.618	6.667	6.545	6.596	6.547	5.510	6.606
NR-AR (≤ 0.300)	0.369	0.950	0.914	0.942	0.902	0.872	0.940	0.795
NR-AR-LBD (≤ 0.300)	0.273	0.927	0.910	0.922	0.900	0.926	0.918	0.905
NR-AhR (≤ 0.300)	0.001	0.001	0.001	0.002	0.002	0.002	0.005	0.001
NR-Aromatase (≤ 0.300)	0.759	0.637	0.682	0.803	0.848	0.832	0.809	0.868
NR-ER (≤ 0.300)	0.412	0.871	0.887	0.869	0.890	0.923	0.876	0.928
NR-ER-LBD (≤ 0.300)	0.593	0.842	0.863	0.835	0.842	0.865	0.798	0.880
NR-PPAR gamma (≤ 0.300)	0.965	0.858	0.824	0.805	0.740	0.941	0.689	0.940
SR-ARE (≤ 0.300)	0.556	0.304	0.356	0.178	0.253	0.615	0.192	0.678
SR-ATAD5 (≤ 0.300)	0.052	0.731	0.580	0.827	0.753	0.891	0.714	0.844
SR-HSE (≤ 0.300)	0.747	0.414	0.314	0.589	0.441	0.851	0.458	0.849
SR-MMP (≤ 0.300)	0.971	0.944	0.970	0.895	0.942	0.951	0.921	0.974
SR-p53 (≤ 0.300)	0.271	0.835	0.831	0.622	0.561	0.894	0.255	0.901
Acute Toxicity Rule (alerts)	0	0	0	0	0	0	0	0
Genotoxic Carcinogenicity Rule (alerts)	0	0	0	0	0	2	0	2
Non Genotoxic Carcinogenicity Rule (alerts)	0	0	0	0	0	0	0	0
Skin SensitizationRule (alerts)	0	0	0	2	2	1	2	1

Aquatic Toxicity Rule (alerts)	1	3	3	3	4	2	3	2
Non Biodegradable Rule (alerts)	0	0	0	1	1	0	1	0
SureChEMBL Rule	0	0	0	0	0	2	0	2
FAF-Drugs4 Rule	0	0	0	0	0	2	0	2
Toxicophores	0	0	0	0	0	2	0	2

Table S2. ADMETox data for oleanolic acid (**1**) and its derivatives **9** –**14**.

High (optimal) value	Moderate value	Low value	Neutral (informative) value

For values given in a range 0.000 – 1.000 (unless stated otherwise): 0.000 – 0.300: low probability, 0.301 – 0.699: moderate probability, 0.700 – 1.000: high probability

**Physicochemical Properties (optimal values)**	**Compound number**					
	**9**	**10**	**11**	**12**	**13**	**14**
**Molecular Weight (100 ~ 600)**	534.270	532.260	547.270	529.260	547.270	563.240
**Volume**	519.115	516.479	527.475	521.969	527.475	537.194
**Density**	1.029	1.031	1.038	1.014	1.038	1.048
**NHA (0 ~ 12)**	3	3	4	3	4	3
**nHD (0 ~ 7)**	1	0	1	0	1	1
**nRot (0 ~ 11)**	0	0	0	3	0	0
**nRing (0 ~ 6)**	6	6	6	5	6	6
**maxRing (0 ~ 18)**	19	19	19	15	20	20
**nHet (1 ~ 15)**	4	4	5	4	5	5
**fChar (-4 ~ +4)**	0	0	0	0	0	0
**nRig (0 ~ 30)**	30	31	31	27	32	32
**Flexibility (≤ 2)**	0.000	0.000	0.000	0.111	0.000	0.000
**Stereo Centers (≤ 2)**	10	9	9	9	9	9
**TPSA (0 ~ 140)**	46.530	43.370	58.890	50.090	55.400	38.330

**LogS (-4 ~ 0.5)**	-6.183	-6.200	-6.241	-6.792	-5.742	-6.112
**LogP (0 ~ 3)**	6.069	5.684	5.244	6.034	5.360	5.831
**LogD (1 ~ 3)**	4.725	4.602	4.048	4.614	4.592	5.021
**QED (≥ 0.67)**	0.262	0.239	0.146	0.211	0.268	0.187
**Sascore (≤ 6)**	6.327	6.304	6.503	6.565	6.416	6.610
**Fsp3 (≥ 0.420)**	0.967	0.933	0.933	0.867	0.933	0.933
**MCE-18 (≥ 45)**	168.559	167.586	167.586	142.071	167.586	167.586
**Npscore (-5 ~ 5)**	2.970	2.965	2.740	2.540	2.334	2.226
**Lipinski Rule**	R	R	R	R	R	R
**Pfizer Rule**	R	R	R	R	R	R
**GSK Rule**	R	R	R	R	R	R
**Golden Triangle**	R	R	R	R	R	R
**PAINS (alerts)**	0	0	0	0	0	0
**ALARM NMR (alerts)**	2	2	2	2	2	3
**BMS (alerts)**	1	1	2	1	1	1
**Chelator Rule (alerts)**	0	0	0	0	0	0
**Caco-2 Permeability (≥ -5.15)**	-5.141	-5.187	-5.250	-5.149	-5.184	-5.121
**MDCK Permeability (≤ 2 × 10^{-6} cm/s)**	1.52 x 10^{-5}	1.38 x 10^{-5}	1.84 x 10^{-5}	2.30 x 10^{-5}	1.62 x 10^{-5}	1.33 x 10^{-5}
**Pgp-inhibitor (≤ 0.300)**	0.965	0.892	0.927	0.977	0.971	0.868
**Pgp-substrate (≤ 0.300)**	0.000	0.000	0.000	0.000	0.000	0.000
**HIA (≤ 0.300)**	0.167	0.470	0.440	0.317	0.215	0.699
**$F_{20\%}$ (≤ 0.300)**	0.068	0.456	0.893	0.159	0.019	0.015
**$F_{30\%}$ (≤ 0.300)**	0.902	0.962	0.965	0.809	0.802	0.818
**PPB (≤ 90%)**	96.906	95.681	99.033	92.328	97.421	99.056
**VD (0.04 – 20 L/kg)**	1.106	1.153	1.289	1.554	0.768	1.356
**BBB Penetration (≤ 0.300)**	0.738	0.885	0.425	0.850	0.768	0.849
**Fu (≥ 5.000 %)**	3.226	3.698	2.369	6.952	4.489	2.852

**CYP1A2 inhibitor (≥ 0.700)**	0.039	0.051	0.064	0.068	0.024	0.062
**CYP1A2 substrate (≥ 0.700)**	0.455	0.706	0.629	0.758	0.877	0.726
**CYP2C19 inhibitor (≥ 0.700)**	0.127	0.281	0.430	0.192	0.398	0.406
**CYP2C19 substrate (≥ 0.700)**	0.918	0.944	0.659	0.691	0.897	0.922
**CYP2C9 inhibitor (≥ 0.700)**	0.504	0.577	0.850	0.542	0.574	0.799
**CYP2C9 substrate (≥ 0.700)**	0.101	0.426	0.168	0.155	0.077	0.241
**CYP2D6 inhibitor (≥ 0.700)**	0.015	0.019	0.070	0.040	0.036	0.158
**CYP2D6 substrate (≥ 0.700)**	0.283	0.351	0.150	0.115	0.411	0.634
**CYP3A4 inhibitor (≥ 0.700)**	0.781	0.827	0.886	0.896	0.864	0.893
**CYP3A4 substrate (≥ 0.700)**	0.626	0.695	0.741	0.811	0.833	0.806
**CL (≥15 mL/min/kg)**	12.195	11.341	2.244	15.679	7.251	7.592
**$T1/2_{<3h}$ (≤ 0.300)**	0.009	0.035	0.017	0.009	0.034	0.027
**hERG Blockers (≤ 0.300)**	0.385	0.103	0.086	0.637	0.151	0.027
**H-HT (≤ 0.300)**	0.609	0.612	0.757	0.715	0.489	0.601
**DILI (≤ 0.300)**	0.024	0.052	0.024	0.082	0.043	0.061
**AMESToxicity (≤ 0.300)**	0.012	0.017	0.008	0.006	0.012	0.010
**Rat Oral Acute Toxicity (≤ 0.300)**	0.897	0.633	0.301	0.902	0.610	0.581
**FDAMDD (≤ 0.300)**	0.938	0.790	0.799	0.862	0.811	0.828
**Skin Sensitization (≤ 0.300)**	0.215	0.107	0.051	0.169	0.190	0.094
**Carcinogencity (≤ 0.300)**	0.379	0.573	0.529	0.901	0.377	0.432
**Eye Corrosion (≤ 0.300)**	0.120	0.069	0.025	0.179	0.005	0.007
**Eye Irritation (≤ 0.300)**	0.032	0.063	0.096	0.020	0.011	0.027
**Respiratory Toxicity (≤ 0.300)**	0.978	0.979	0.979	0.982	0.970	0.980
**Bioconcentration Factors**	2.646	2.329	2.993	2.614	2.011	2.835
**IGC_{50}**	5.230	5.059	5.077	4.745	4.847	5.259
**$LC_{50}FM$**	6.470	5.984	5.991	5.468	5.901	6.443
**$LC_{50}DM$**	6.869	6.365	6.375	6.936	6.263	6.388

**NR-AR (≤ 0.300)**	0.829	0.911	0.804	0.789	0.249	0.029
**NR-AR-LBD (≤ 0.300)**	0.927	0.939	0.940	0.935	0.393	0.628
**NR-AhR (≤ 0.300)**	0.000	0.002	0.002	0.001	0.001	0.001
**NR-Aromatase (≤ 0.300)**	0.712	0.779	0.810	0.740	0.861	0.905
**NR-ER (≤ 0.300)**	0.848	0.856	0.885	0.726	0.398	0.524
**NR-ER-LBD (≤ 0.300)**	0.876	0.846	0.850	0.798	0.511	0.754
**NR-PPAR gamma (≤ 0.300)**	0.951	0.960	0.975	0.950	0.842	0.940
**SR-ARE (≤ 0.300)**	0.448	0.379	0.529	0.656	0.560	0.825
**SR-ATAD5 (≤ 0.300)**	0.617	0.685	0.832	0.599	0.417	0.512
**SR-HSE (≤ 0.300)**	0.755	0.748	0.979	0.771	0.839	0.968
**SR-MMP (≤ 0.300)**	0.957	0.934	0.954	0.916	0.915	0.940
**SR-p53 (≤ 0.300)**	0.723	0.484	0.746	0.784	0.658	0.797
**Acute Toxicity Rule (alerts)**	0	0	0	0	0	0
**Genotoxic Carcinogenicity Rule (alerts)**	3	3	5	3	3	3
**Non Genotoxic Carcinogenicity Rule (alerts)**	2	2	2	2	2	3
**Skin SensitizationRule (alerts)**	1	2	1	1	1	1
**Aquatic Toxicity Rule (alerts)**	5	5	4	4	4	4
**Non Biodegradable Rule (alerts)**	0	1	0	0	0	0
**SureChEMBL Rule**	0	0	2	0	0	1
**FAF-Drugs4 Rule**	0	0	2	1	0	2
**Toxicophores**	0	0	2	0	0	2

Additional information concerning the above tests can be found: Pharmaceutics **2024**, 16, 86. https://doi.org/10.3390/pharmaceutics16010086, **Supplementary Materials.**
