# Supplementary material for: Oleanolic Acid Lactones as Effective Agents in the Combat with Cancers—Cytotoxic and Antioxidant Activity, SAR Analysis, Molecular Docking and ADMETox Profile
Source: Int J Mol Sci. 2025 Apr 25;26(9):4099. doi: 10.3390/ijms26094099 (PMC12072072; doi:10.3390/ijms26094099)
Supplement: Supplementary file 1 [file ijms-26-04099-s001.zip › Suppl. Mat. File S3. Molecular docking.pdf]

International Journal of *Molecular Sciences*

MDPI

Supplementary Materials. File S3

Figures S2–S15 and **S16–S29**: Results of Molecular Docking

Figure S2. (A) Complex of compound 1 with 1M17 in C1 pocket. (B) Diagram in 2D with interactions.

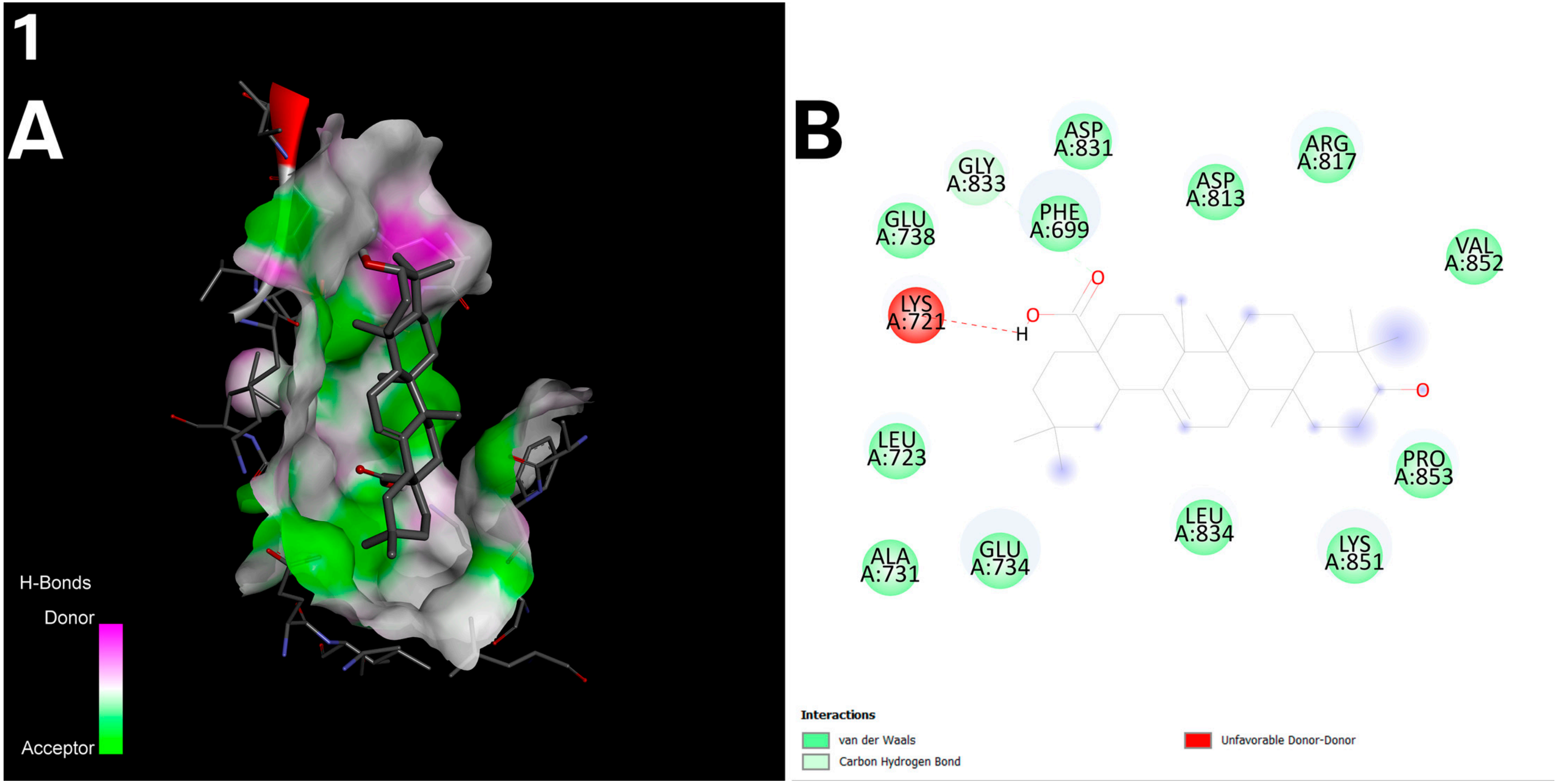

Figure S3. (A) Complex of compound 2 with 1M17 in C1 pocket. (B) Diagram in 2D with interactions.

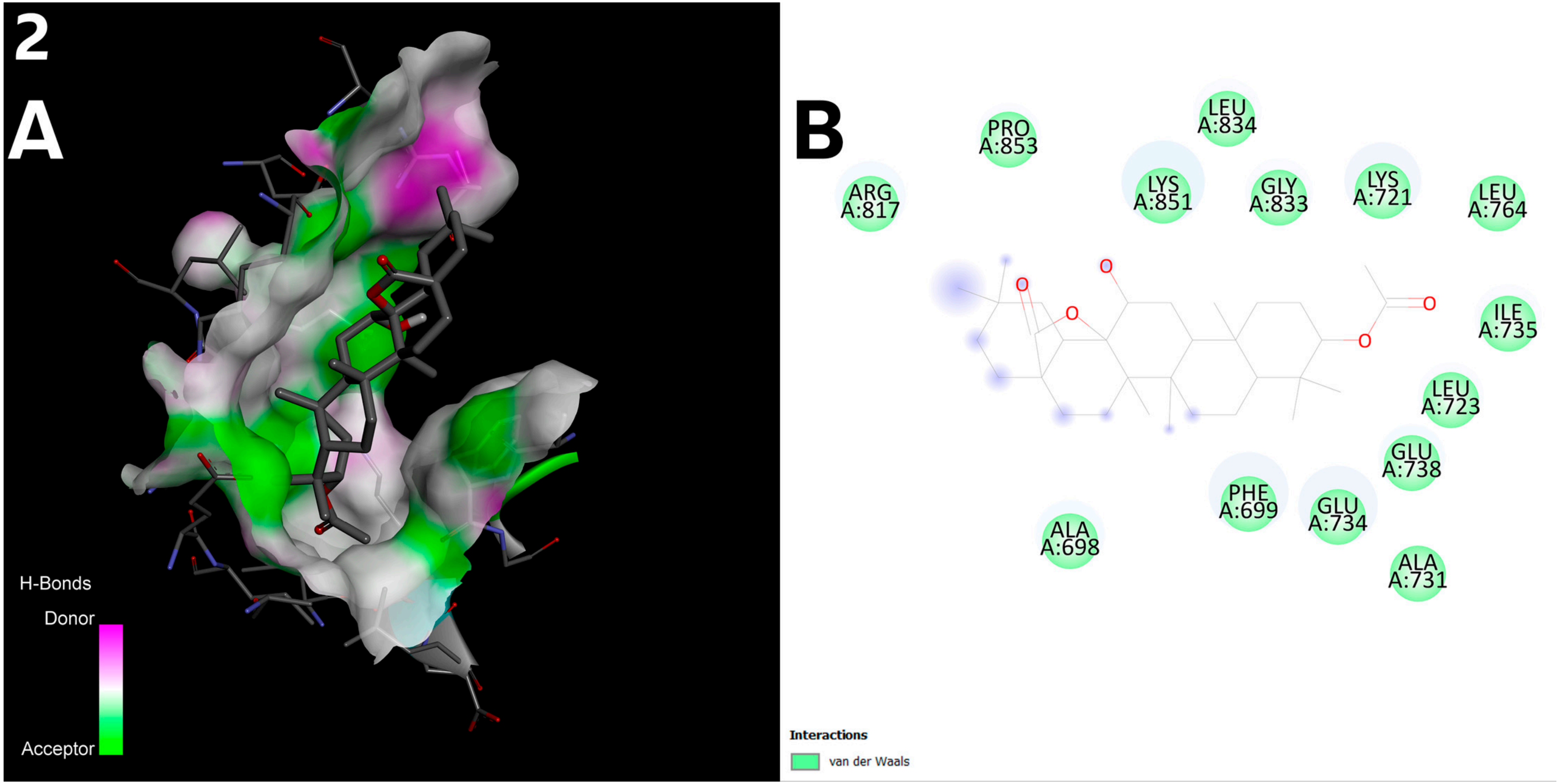

Figure S4. (A) Complex of compound 3 with 1M17 in C1 pocket. (B) Diagram in 2D with interactions.

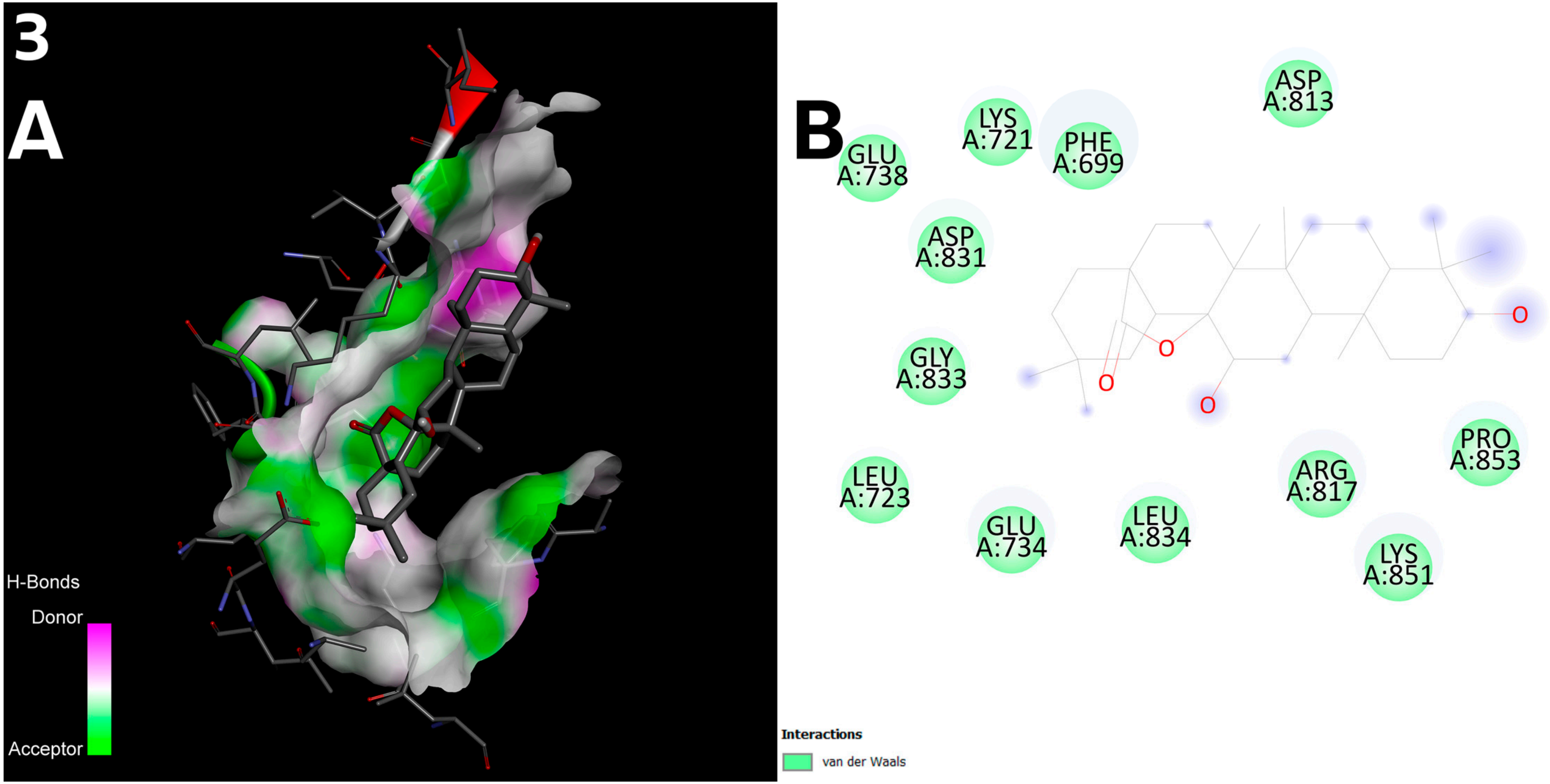

Figure S5. (A) Complex of compound 4 with 1M17 in C1 pocket. (B) Diagram in 2D with interactions.

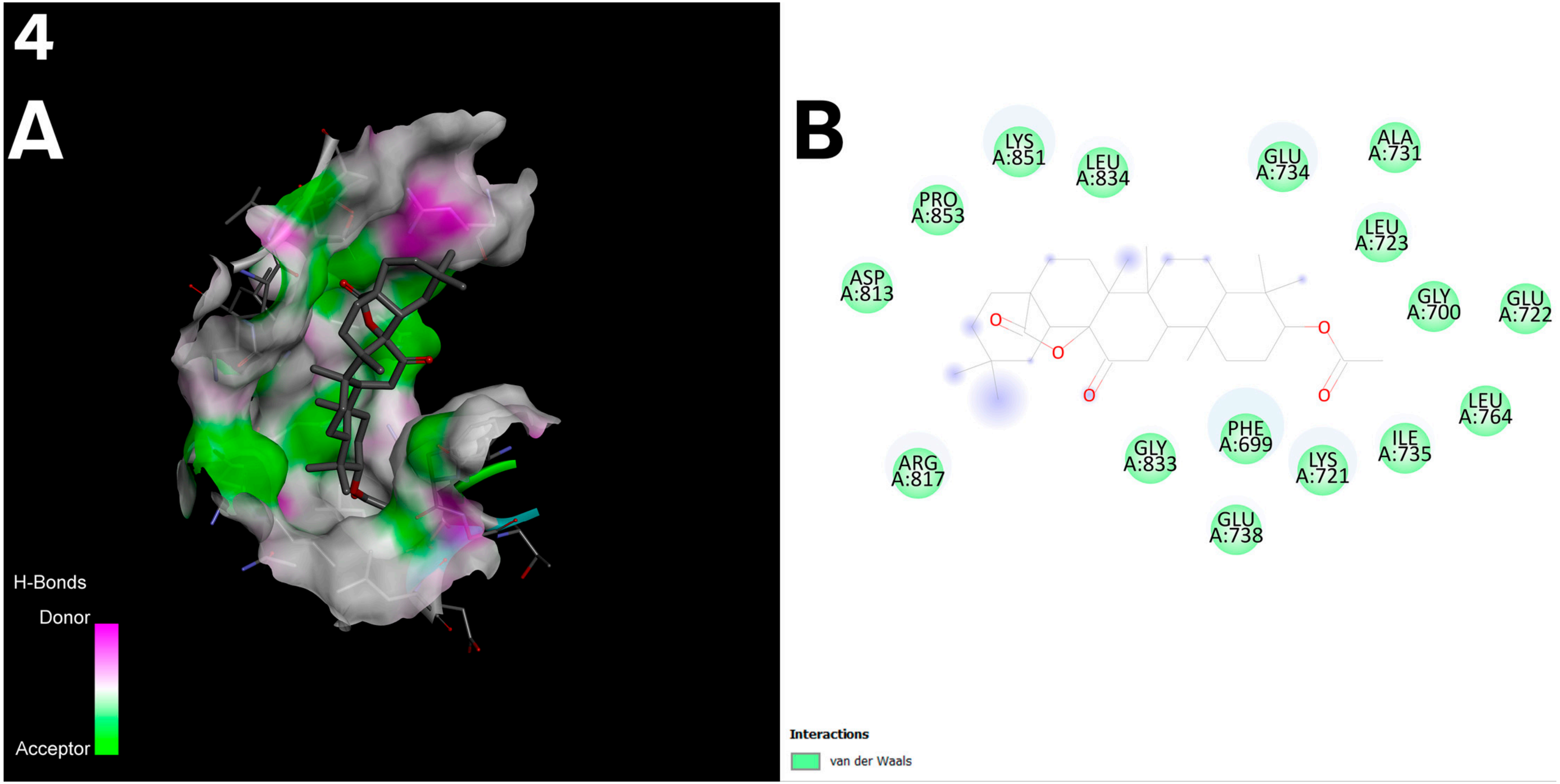

Figure S6. (A) Complex of compound 5 with 1M17 in C1 pocket. (B) Diagram in 2D with interactions.

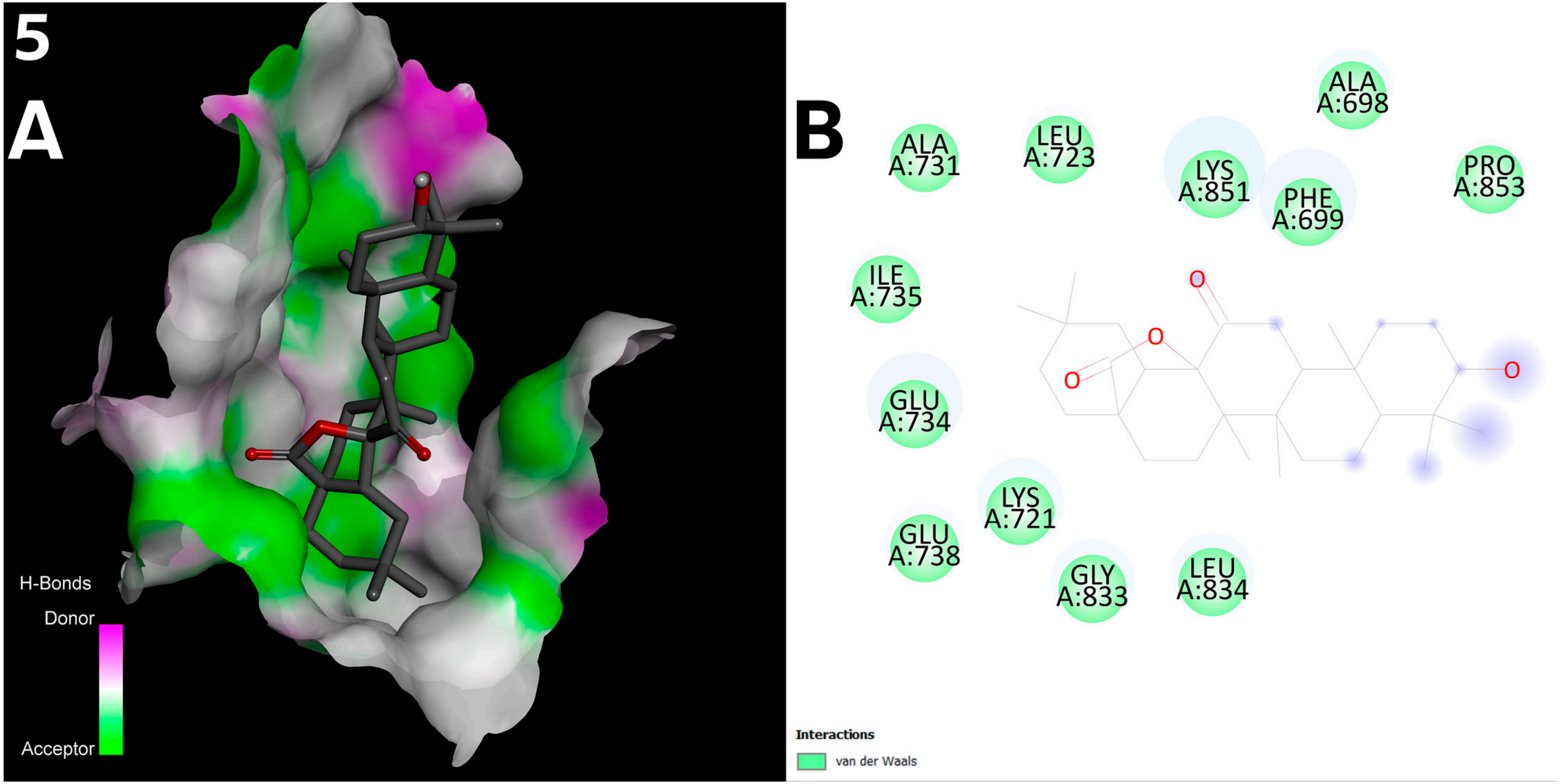

Figure S7. (A) Complex of compound 6 with 1M17 in C1 pocket. (B) Diagram in 2D with interactions.

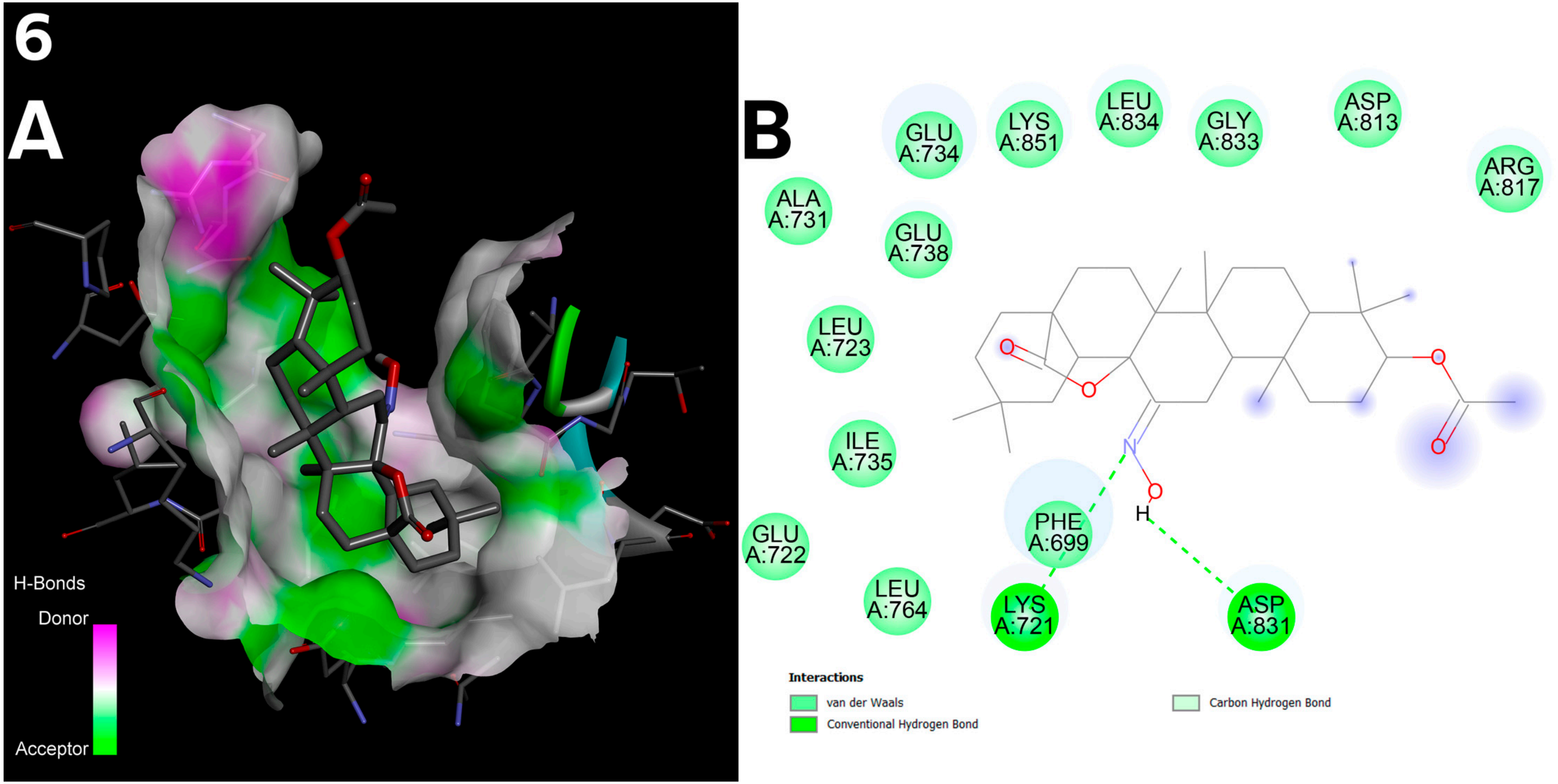

Figure S8. (A) Complex of compound 7 with 1M17 in C1 pocket. (B) Diagram in 2D with interactions.

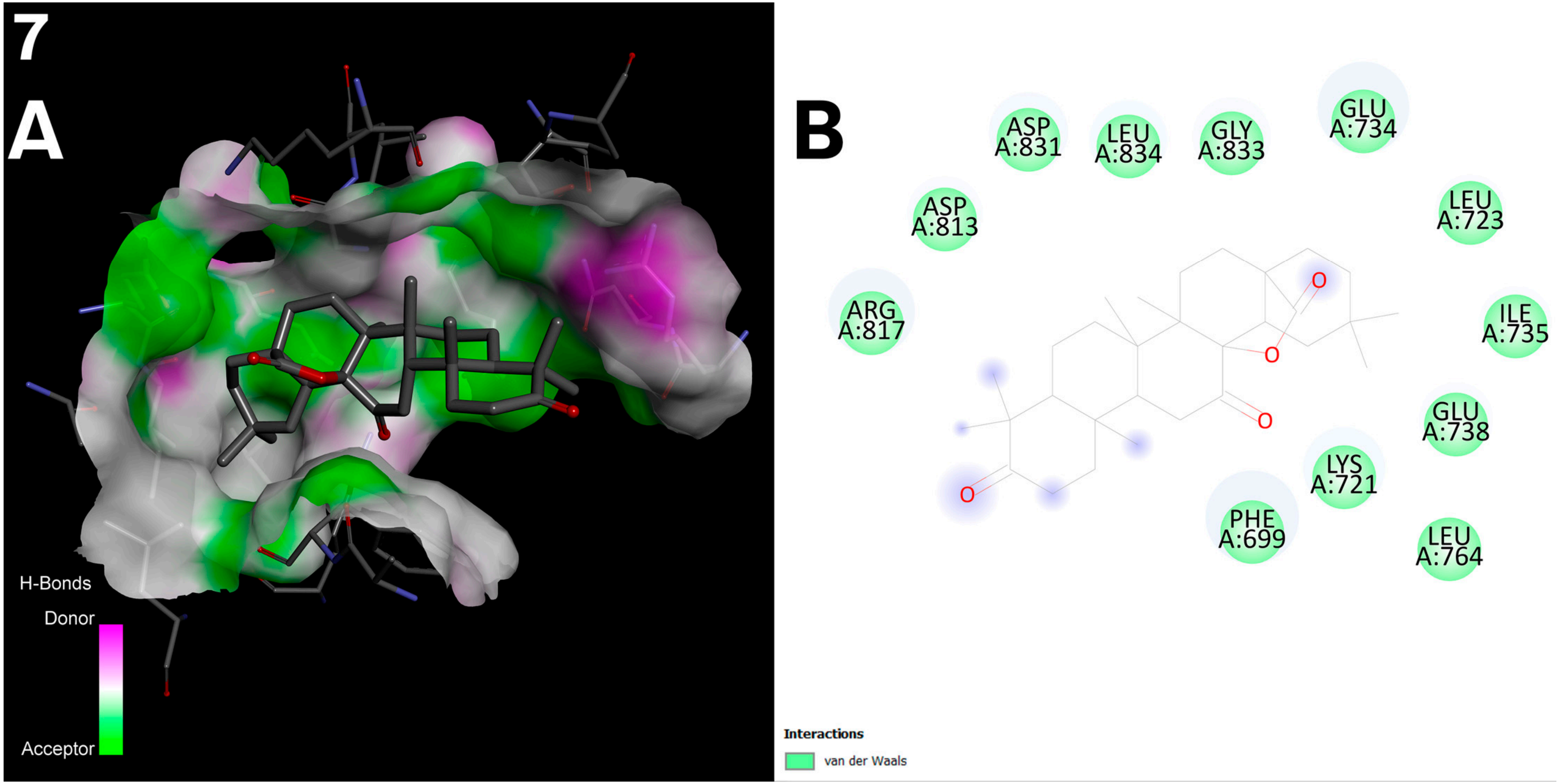

Figure S9. (A) Complex of compound 8 with 1M17 in C1 pocket. (B) Diagram in 2D with interactions.

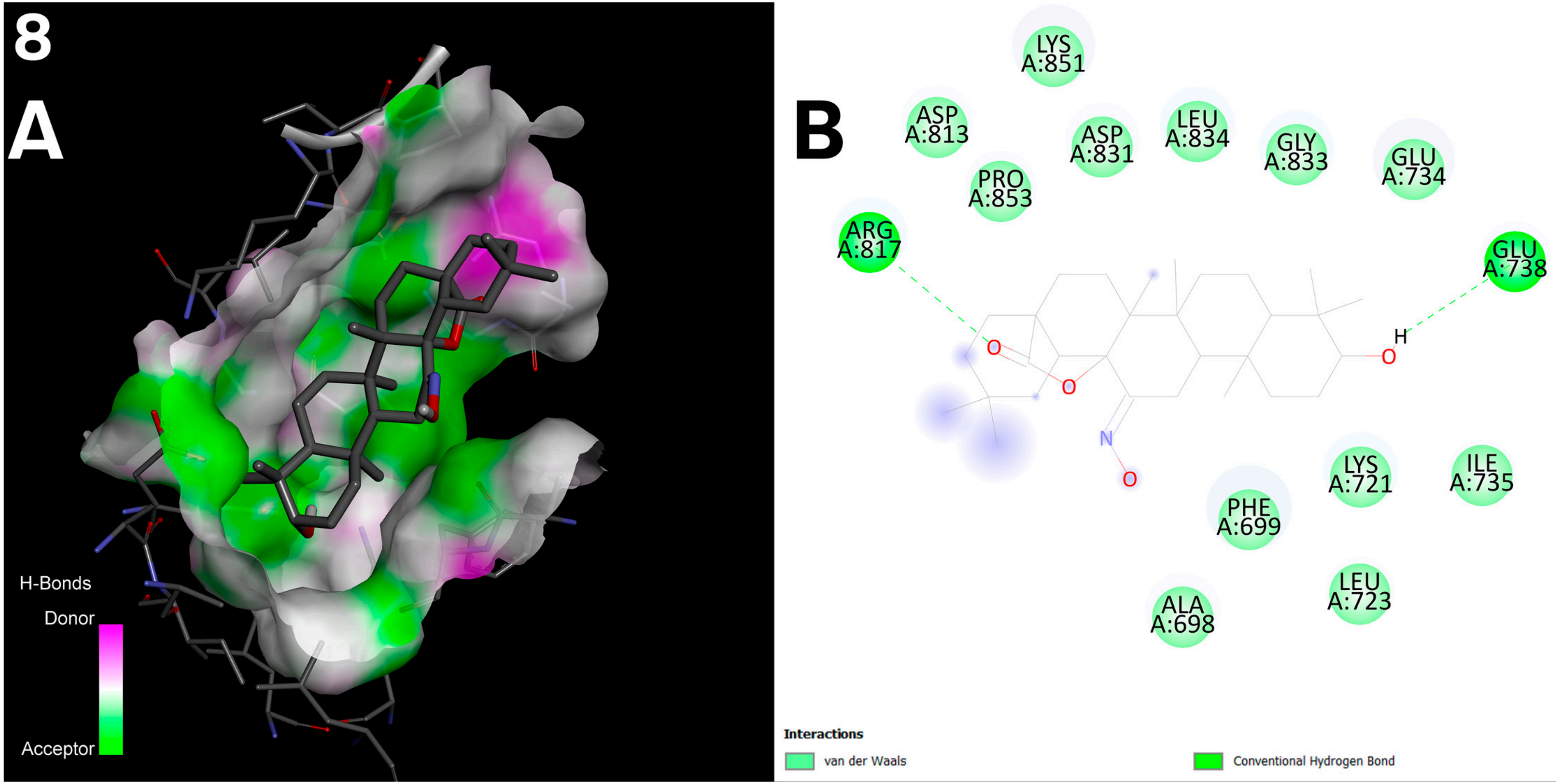

Figure S10. (A) Complex of compound 9 with 1M17 in C1 pocket. (B) Diagram in 2D with interactions.

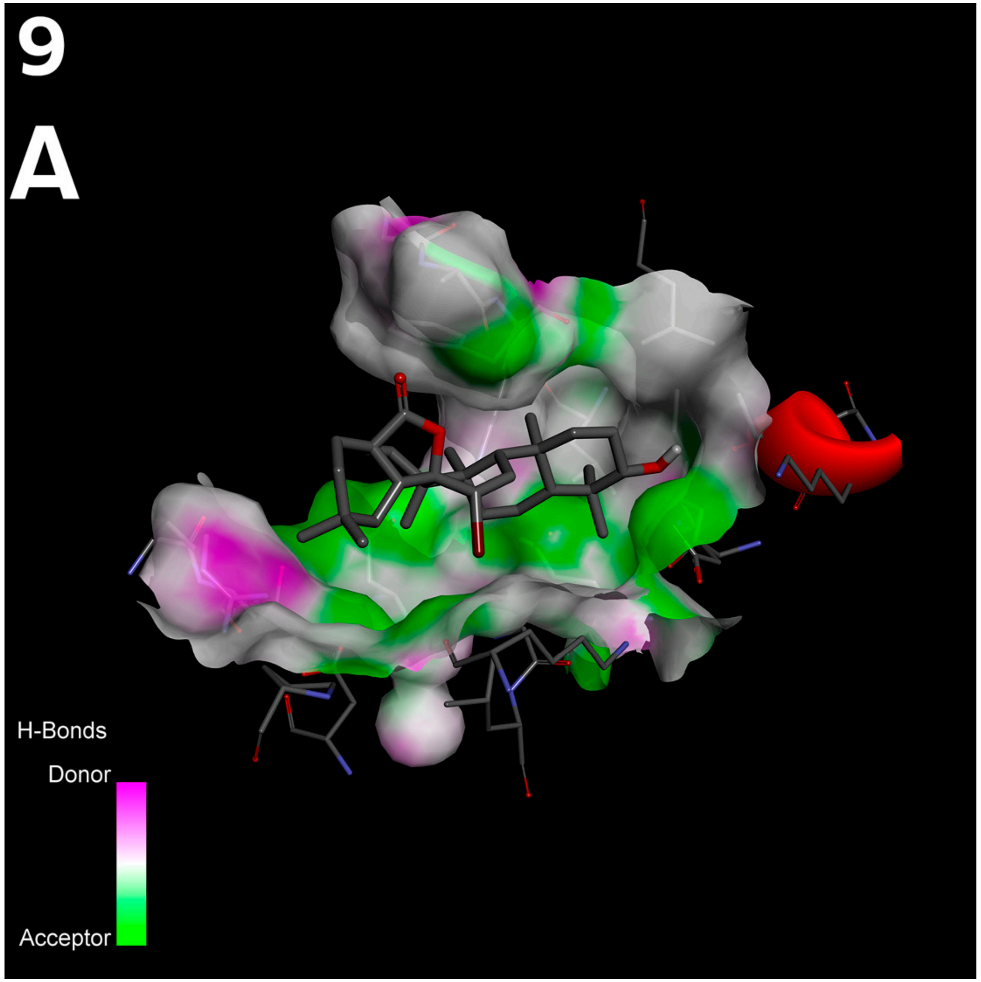

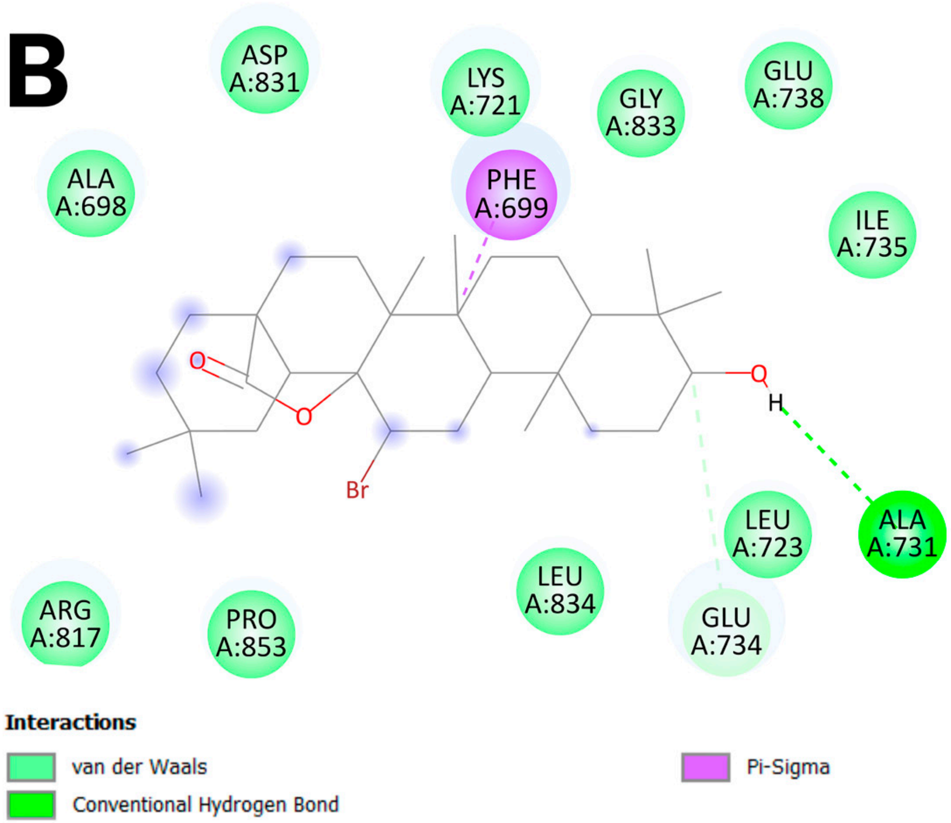

Figure S11. (A) Complex of compound 10 with 1M17 in C1 pocket. (B) Diagram in 2D with interactions.

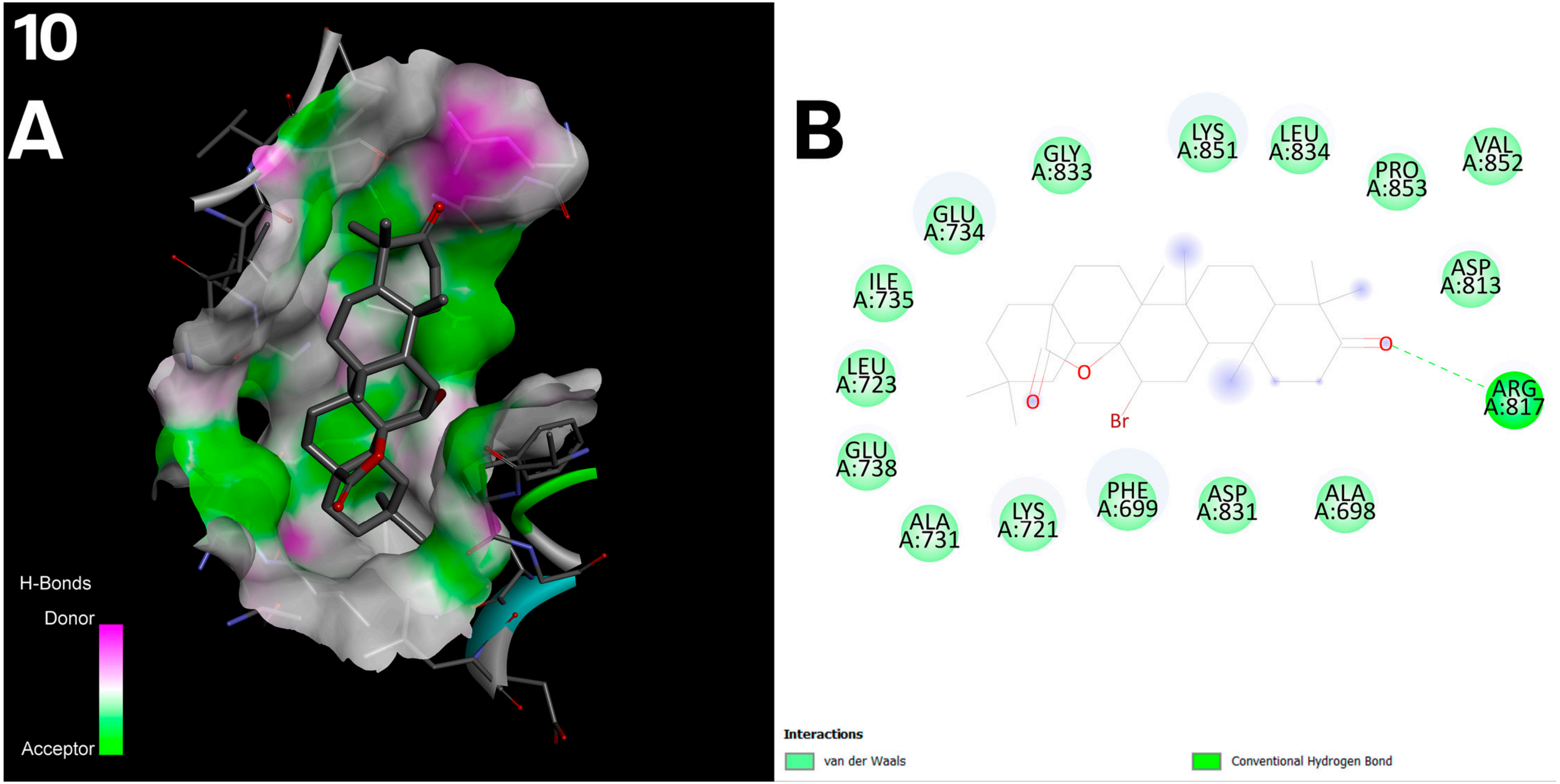

Figure S12. (A) Complex of compound 11 with 1M17 in C1 pocket. (B) Diagram in 2D with interactions.

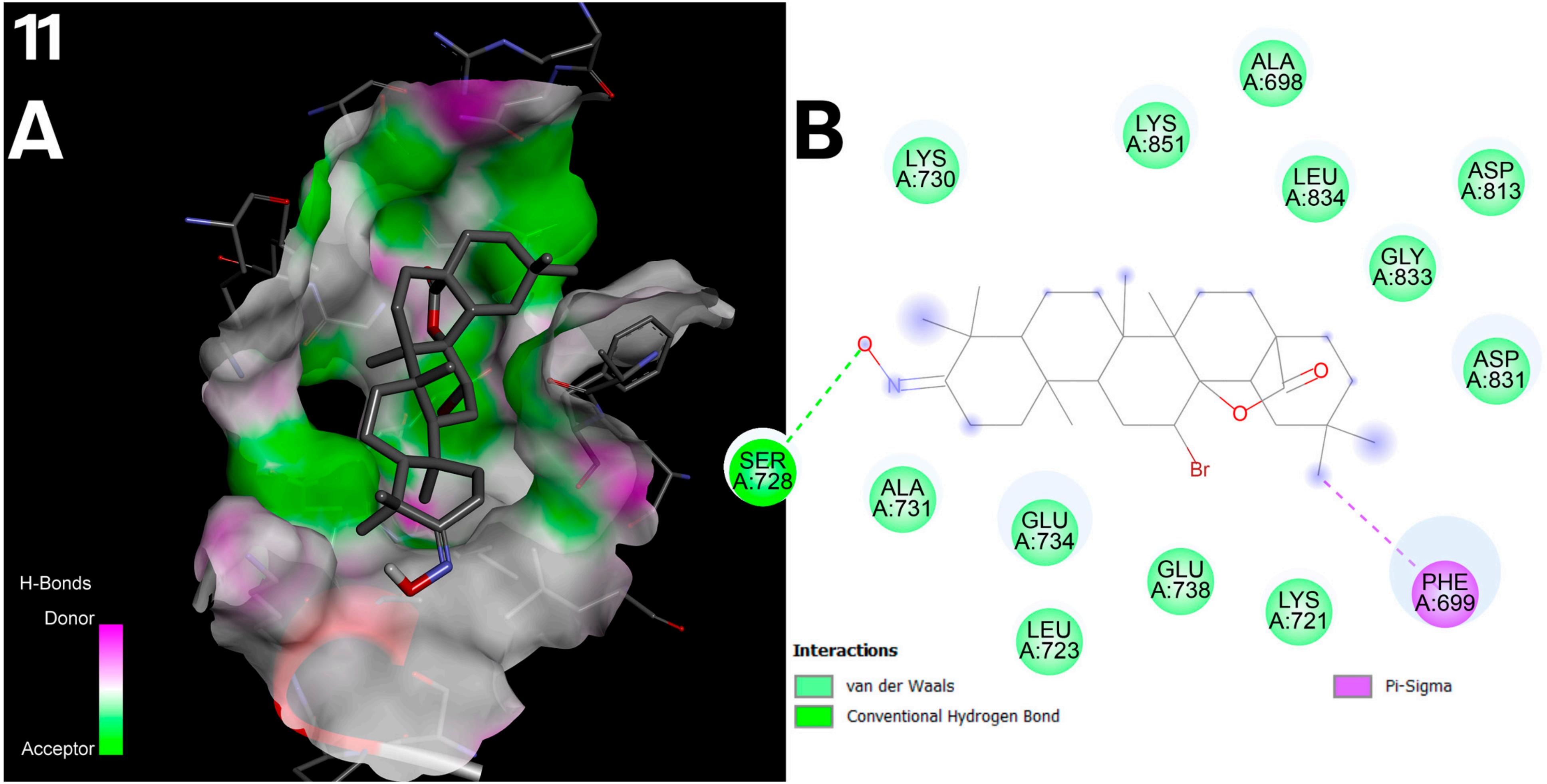

Figure S13. (A) Complex of compound 12 with 1M17 in C1 pocket. (B) Diagram in 2D with interactions.

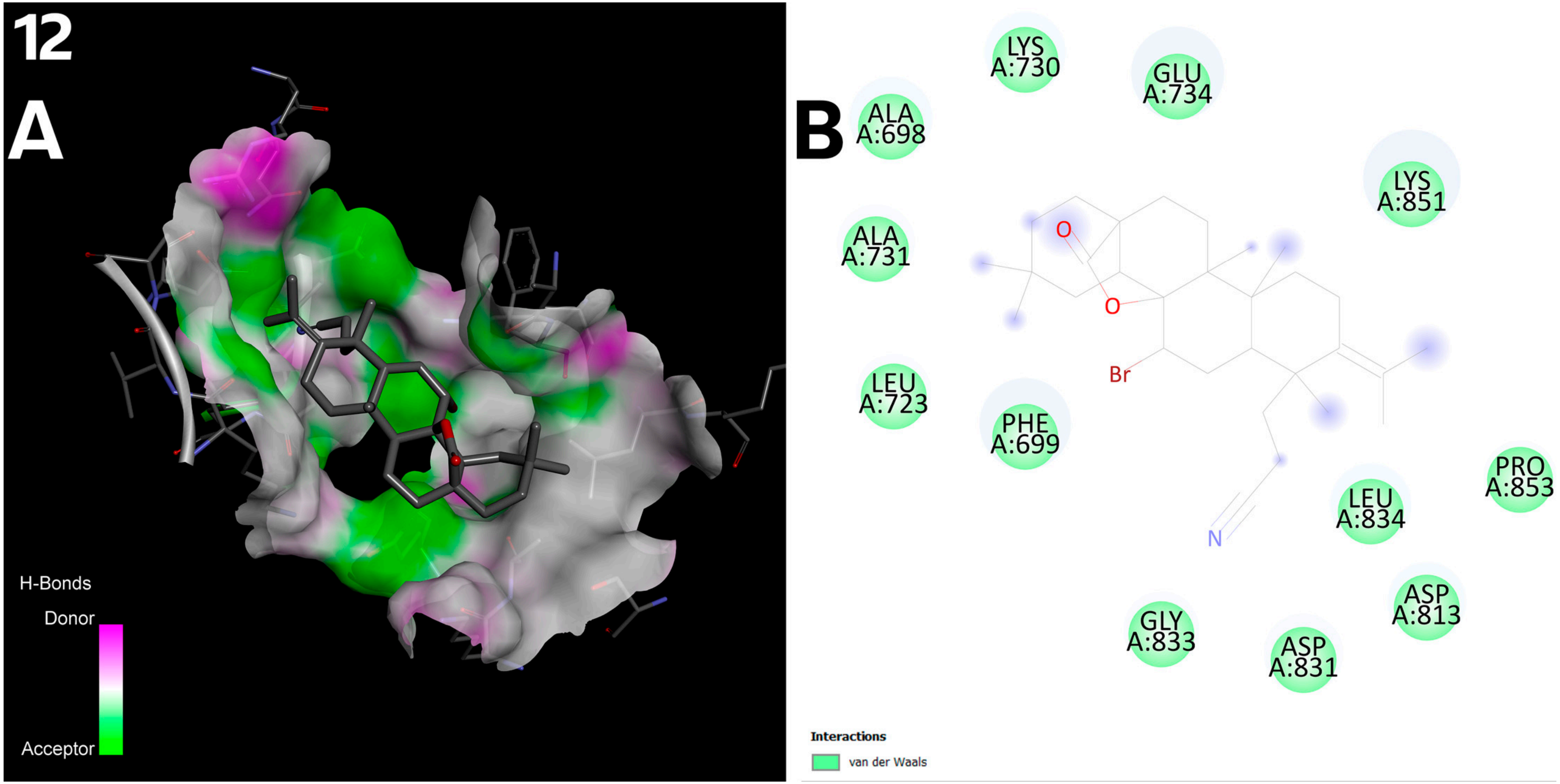

Figure S14. **(A) Complex of compound 13 with 1M17 in C1 pocket. (B) Diagram in 2D with interactions.**

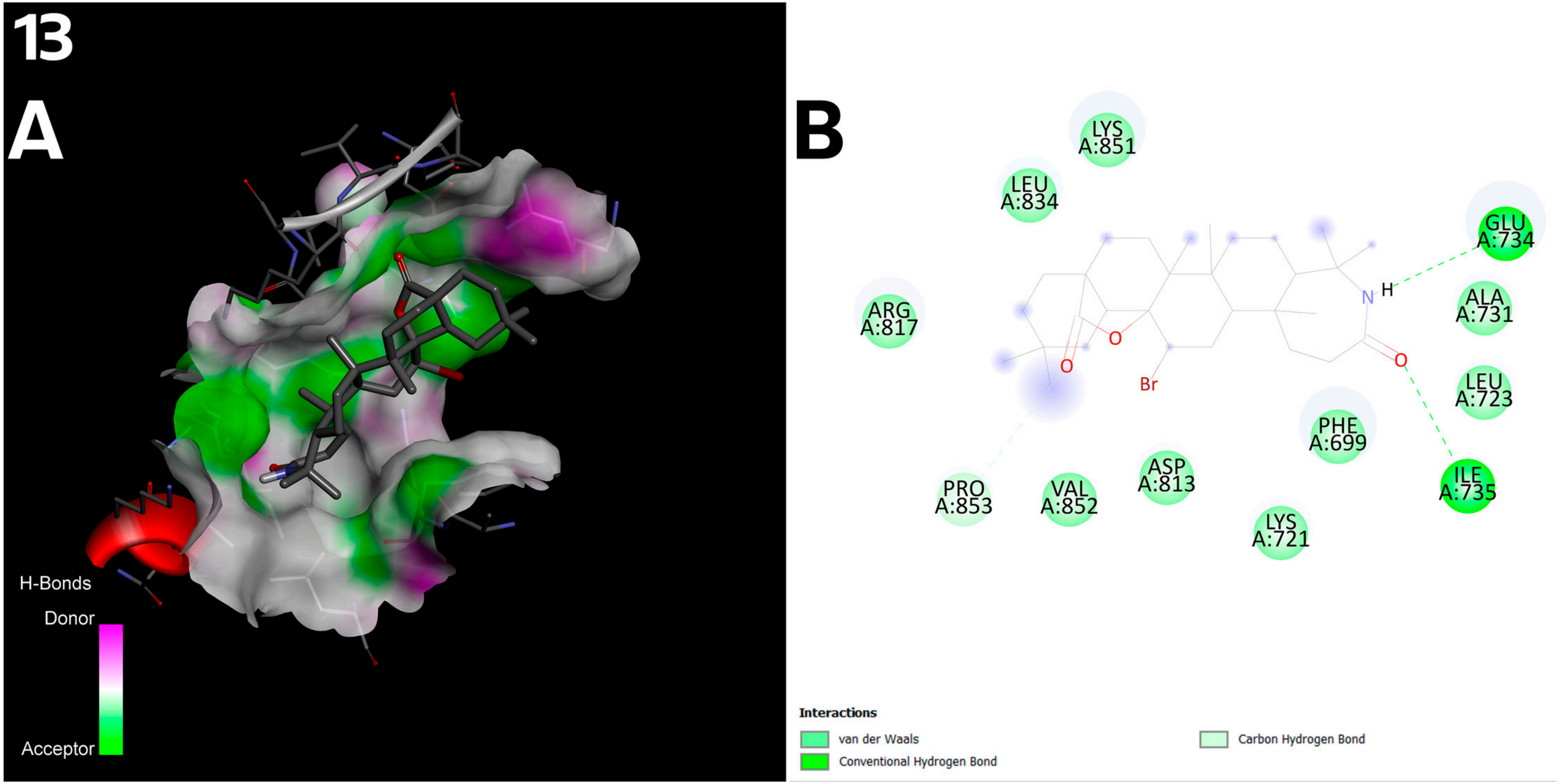

Figure S15. (A) Complex of compound 14 with 1M17 in C1 pocket. (B) Diagram in 2D with interactions.

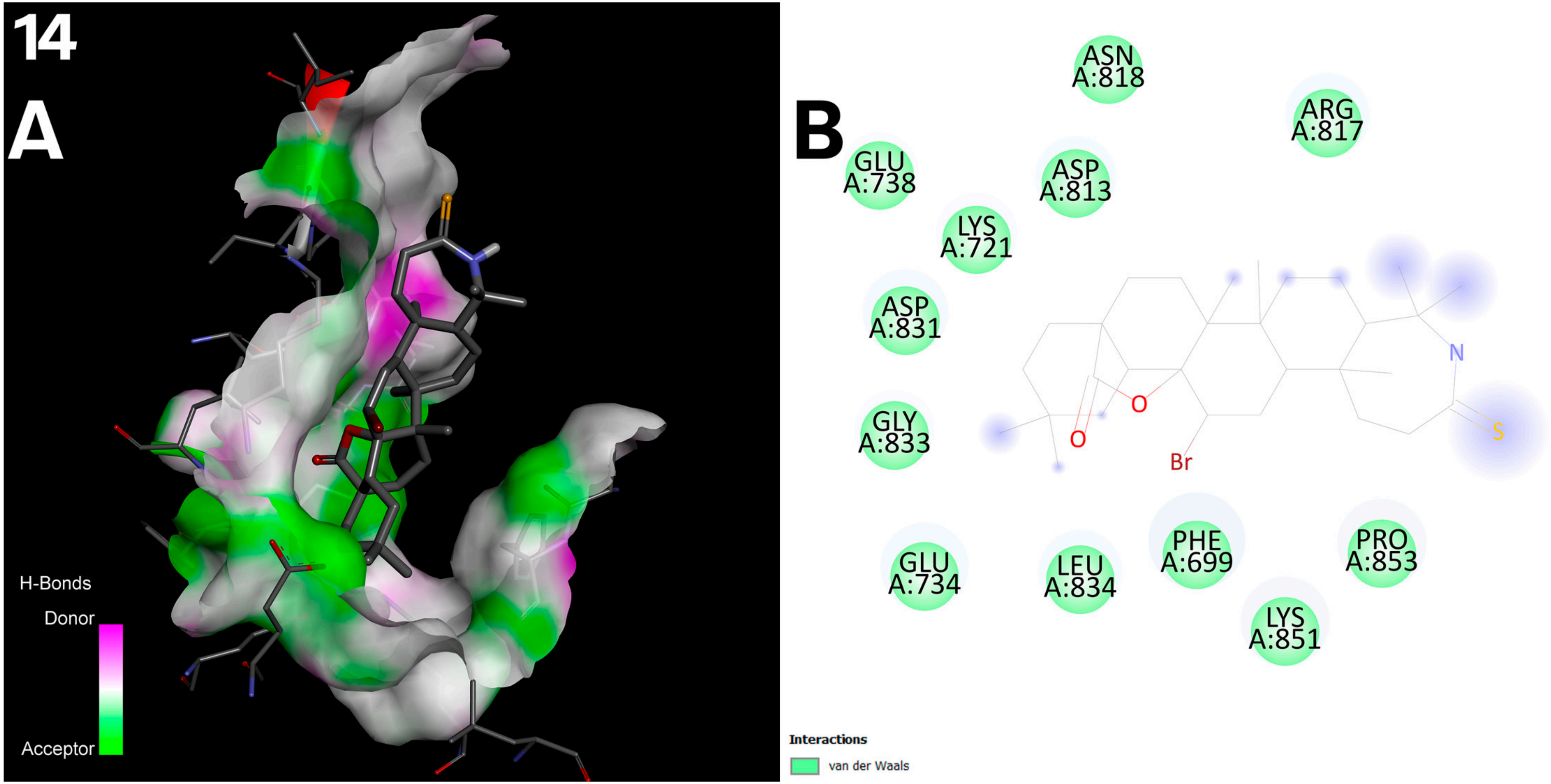

Figure S16. (A) Complex of compound 1 with 1M17 in C2 pocket. (B) Diagram in 2D with interactions.

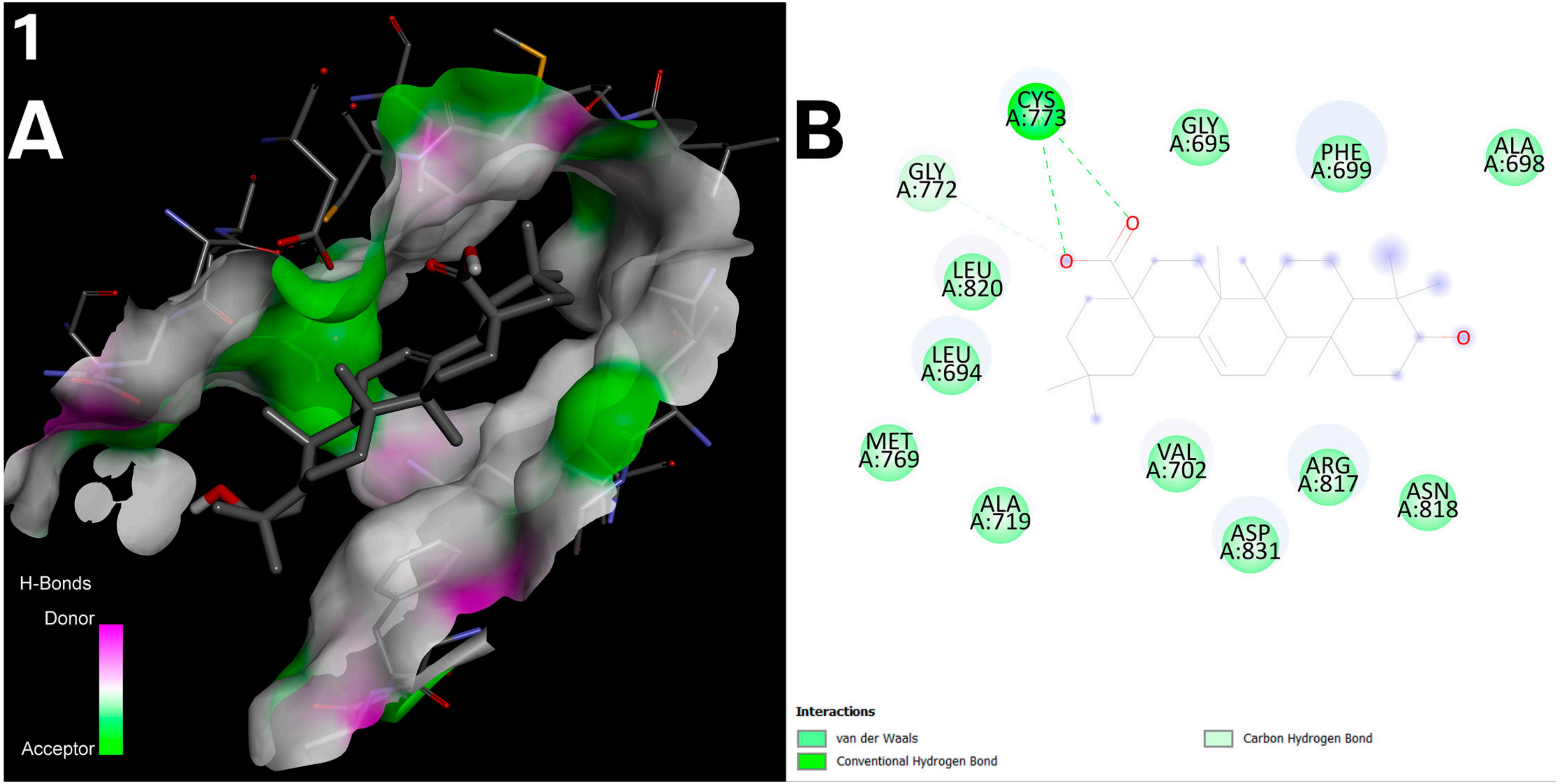

Figure S17. (A) Complex of compound 2 with 1M17 in C2 pocket. (B) Diagram in 2D with interactions.

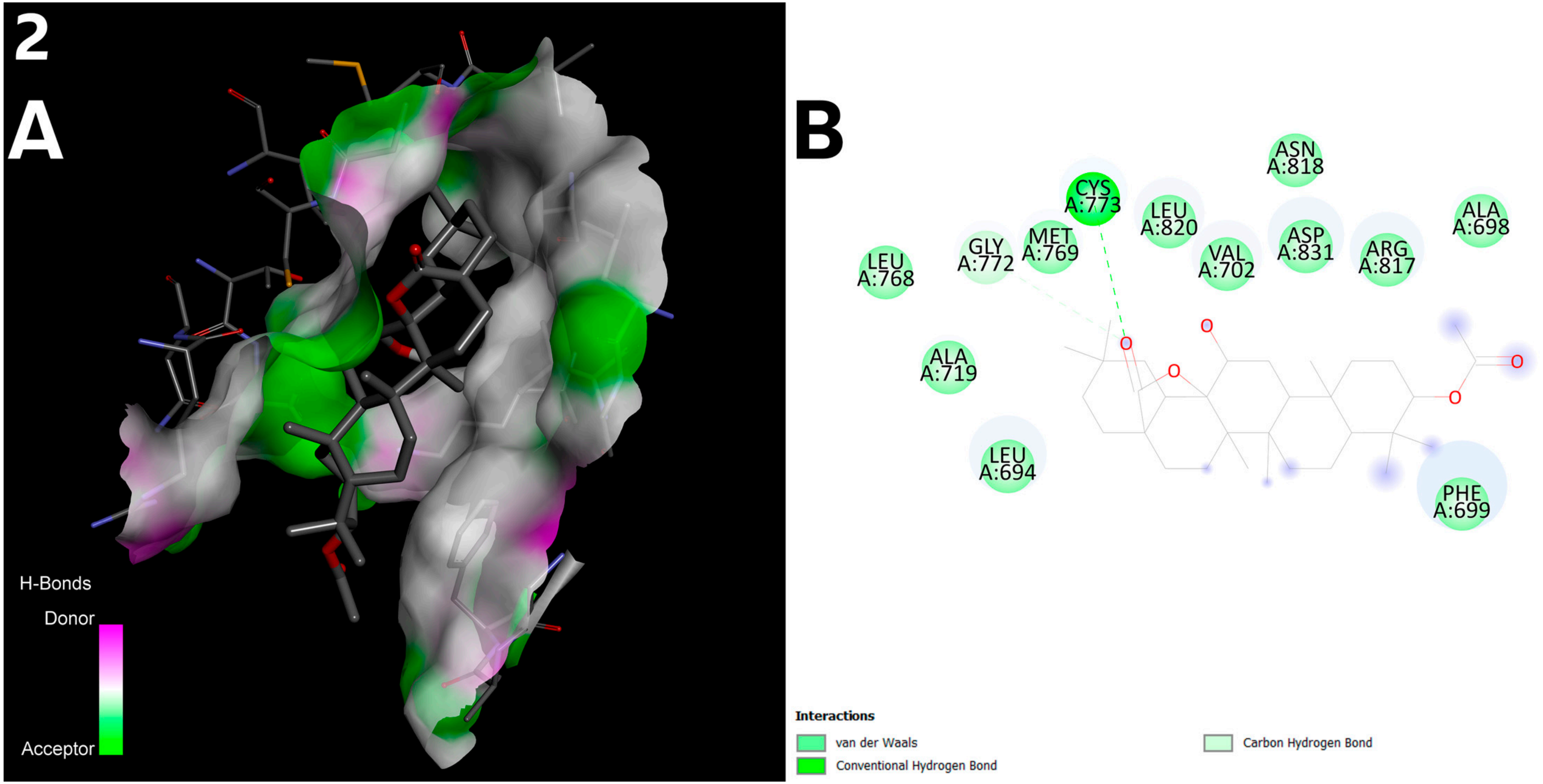

Figure S18. (A) Complex of compound 3 with 1M17 in C2 pocket. (B) Diagram in 2D with interactions.

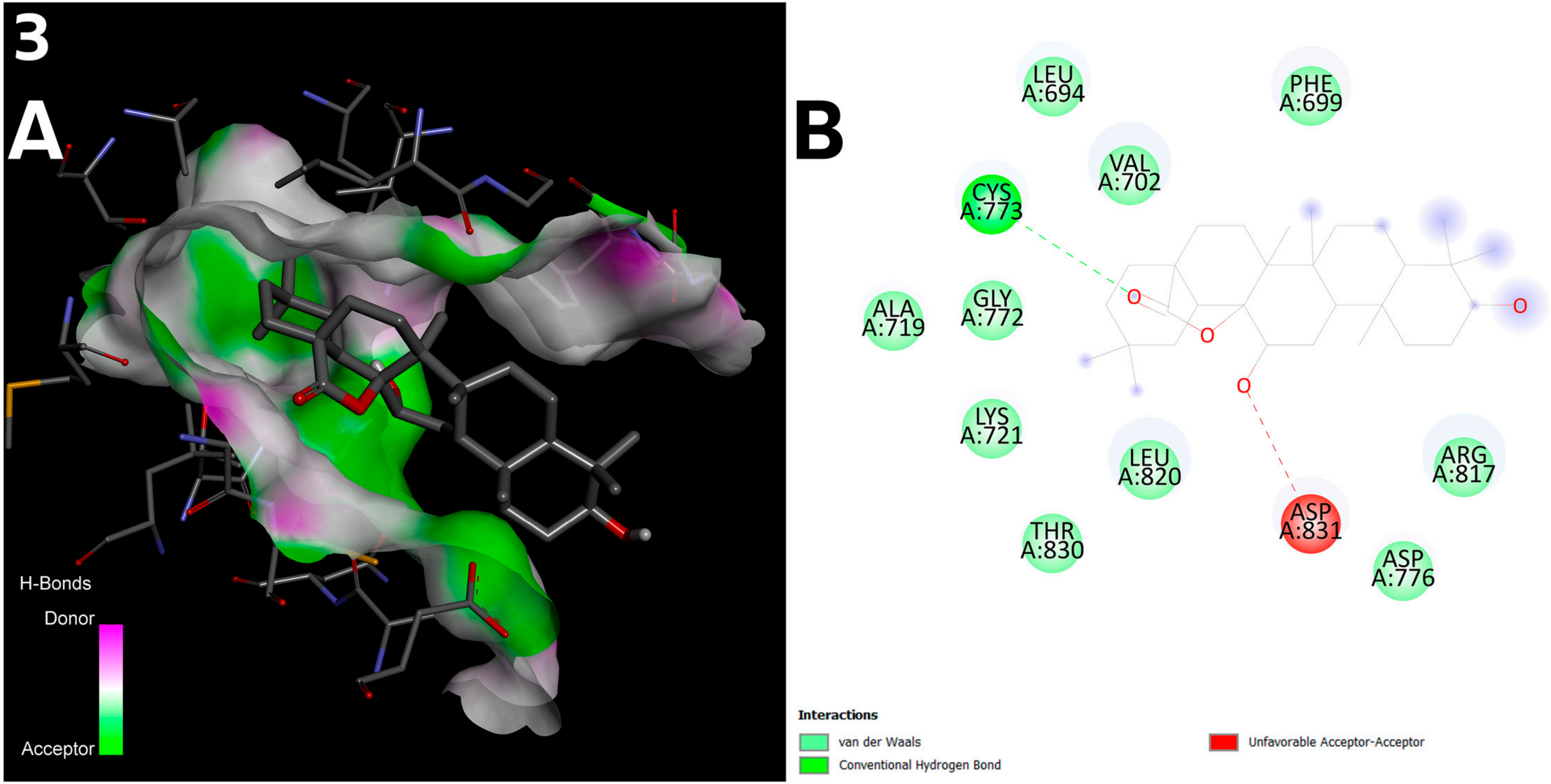

Figure S19. (A) Complex of compound 4 with 1M17 in C2 pocket. (B) Diagram in 2D with interactions.

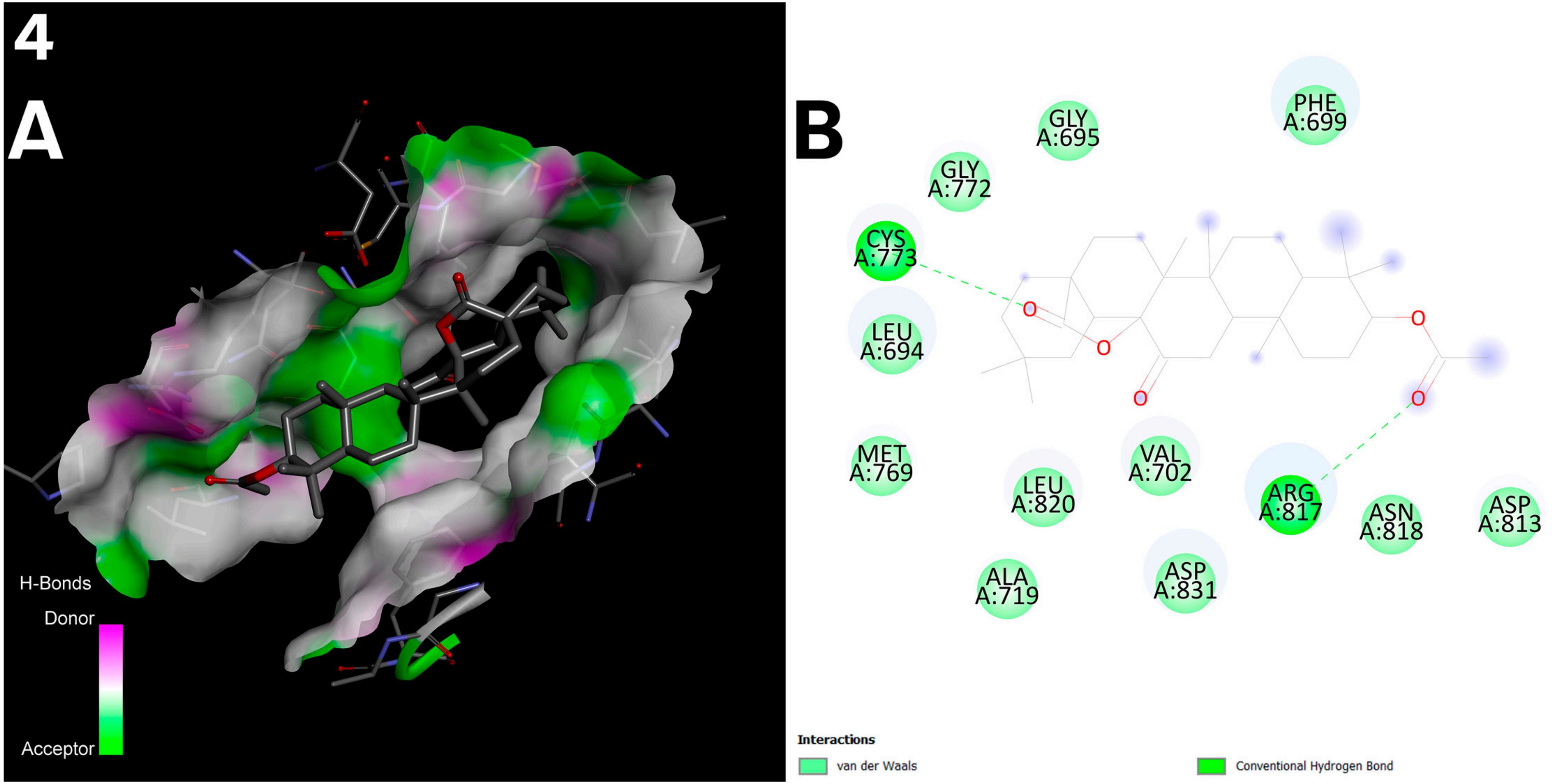

Figure S20. (A) Complex of compound 5 with 1M17 in C2 pocket. (B) Diagram in 2D with interactions.

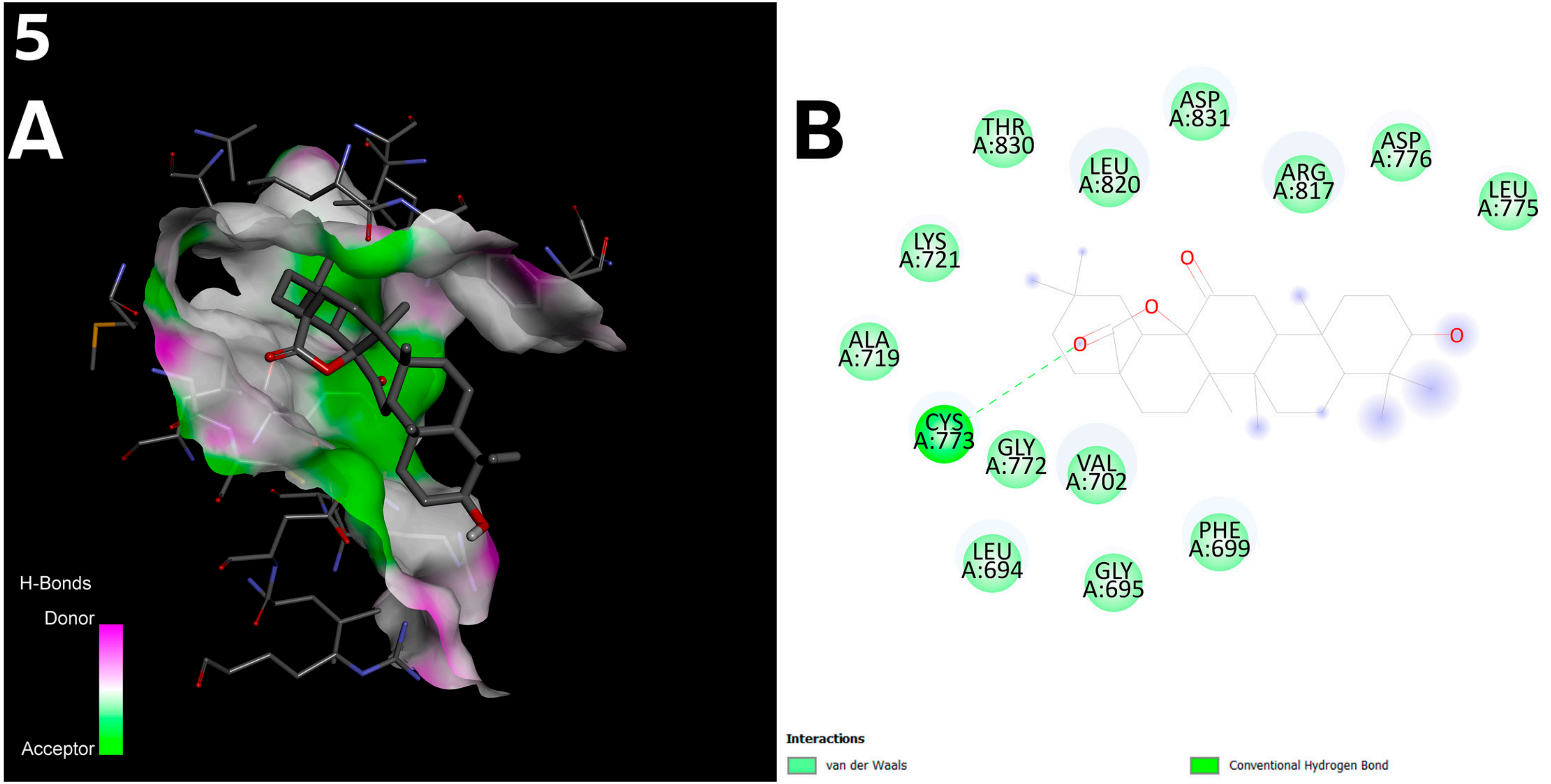

Figure S21. (A) Complex of compound 6 with 1M17 in C2 pocket. (B) Diagram in 2D with interactions.

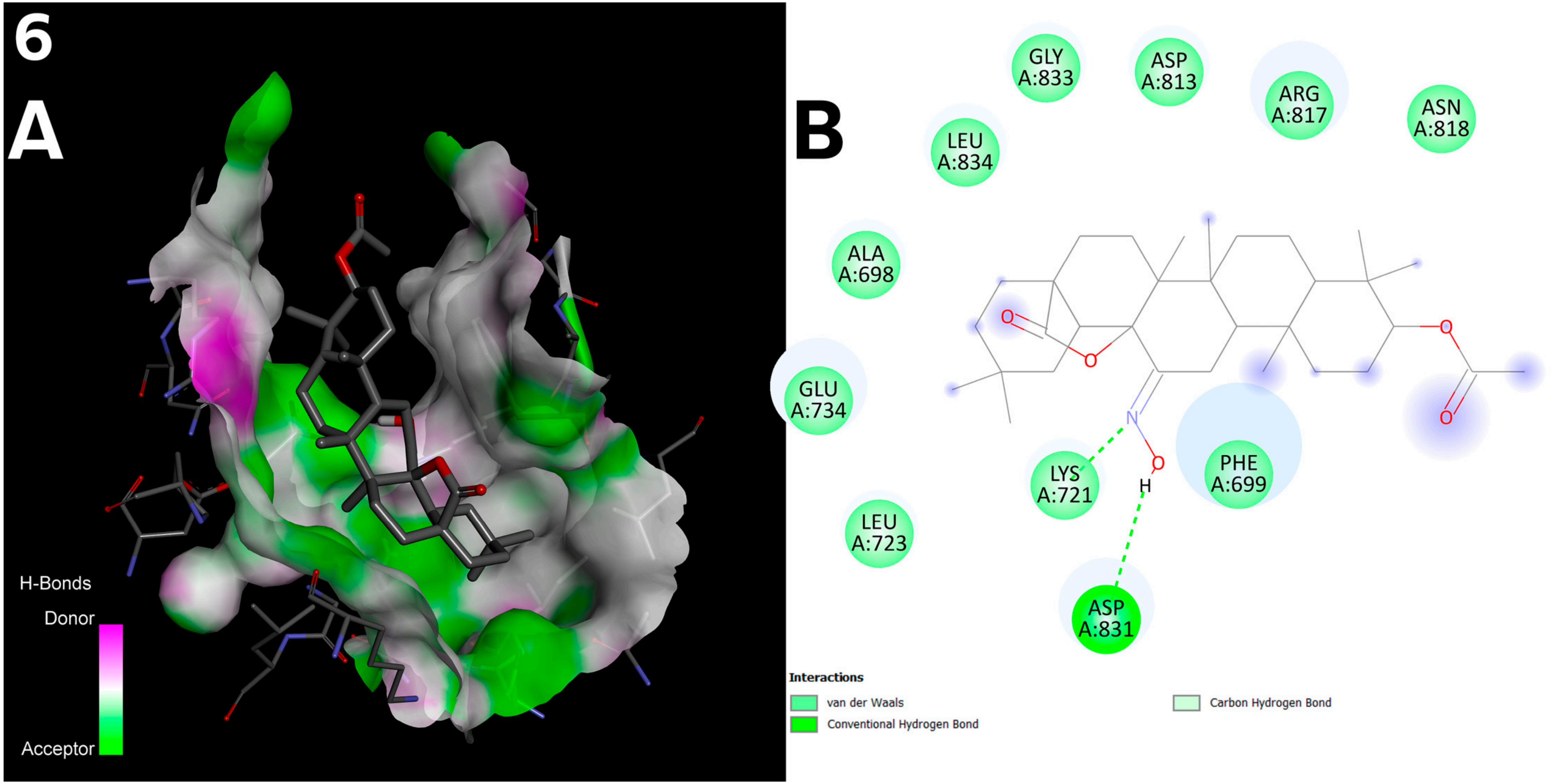

Figure S22. (A) Complex of compound 7 with 1M17 in C2 pocket. (B) Diagram in 2D with interactions.

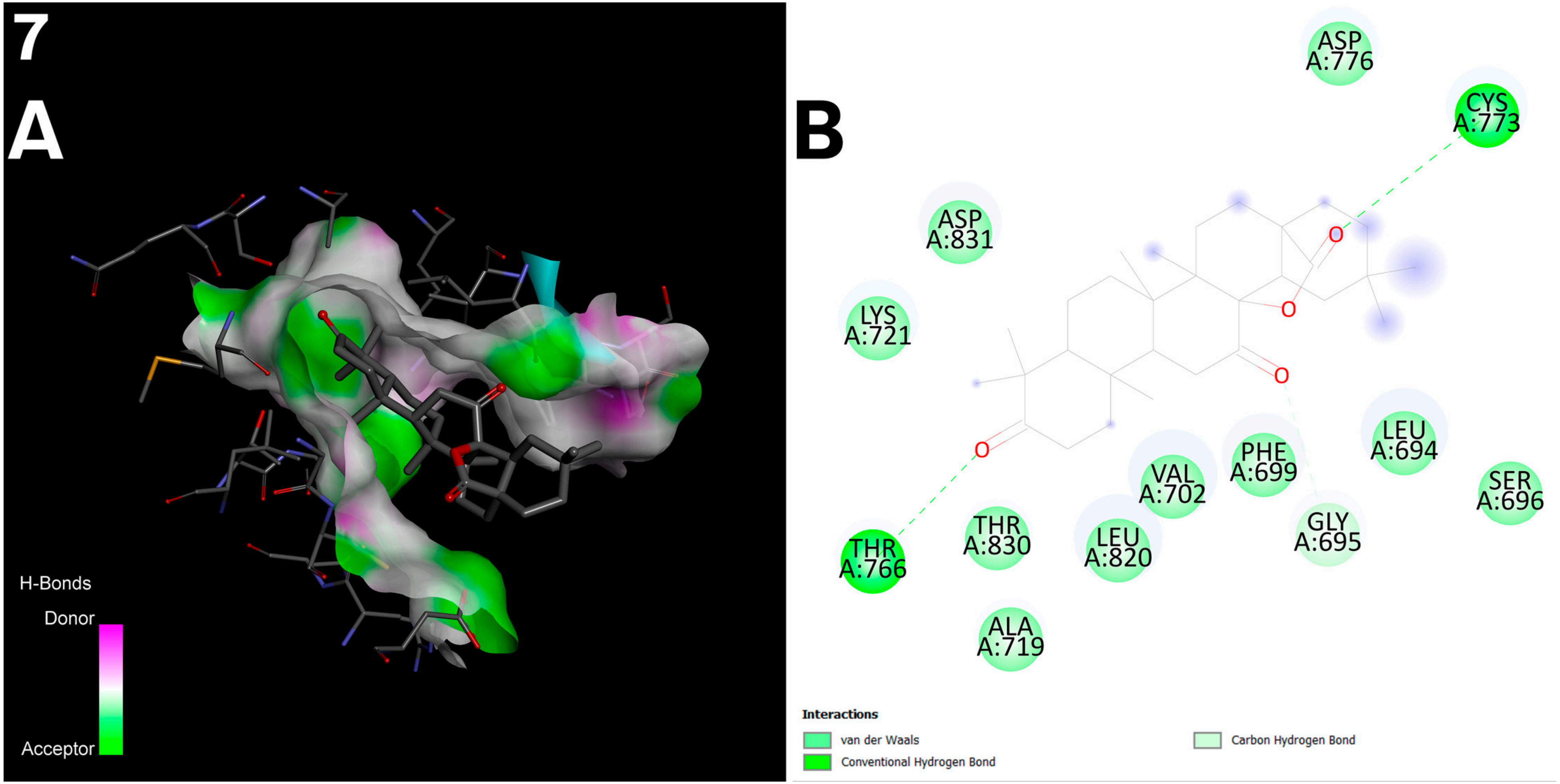

Figure S23. (A) Complex of compound 8 with 1M17 in C2 pocket. (B) Diagram in 2D with interactions.

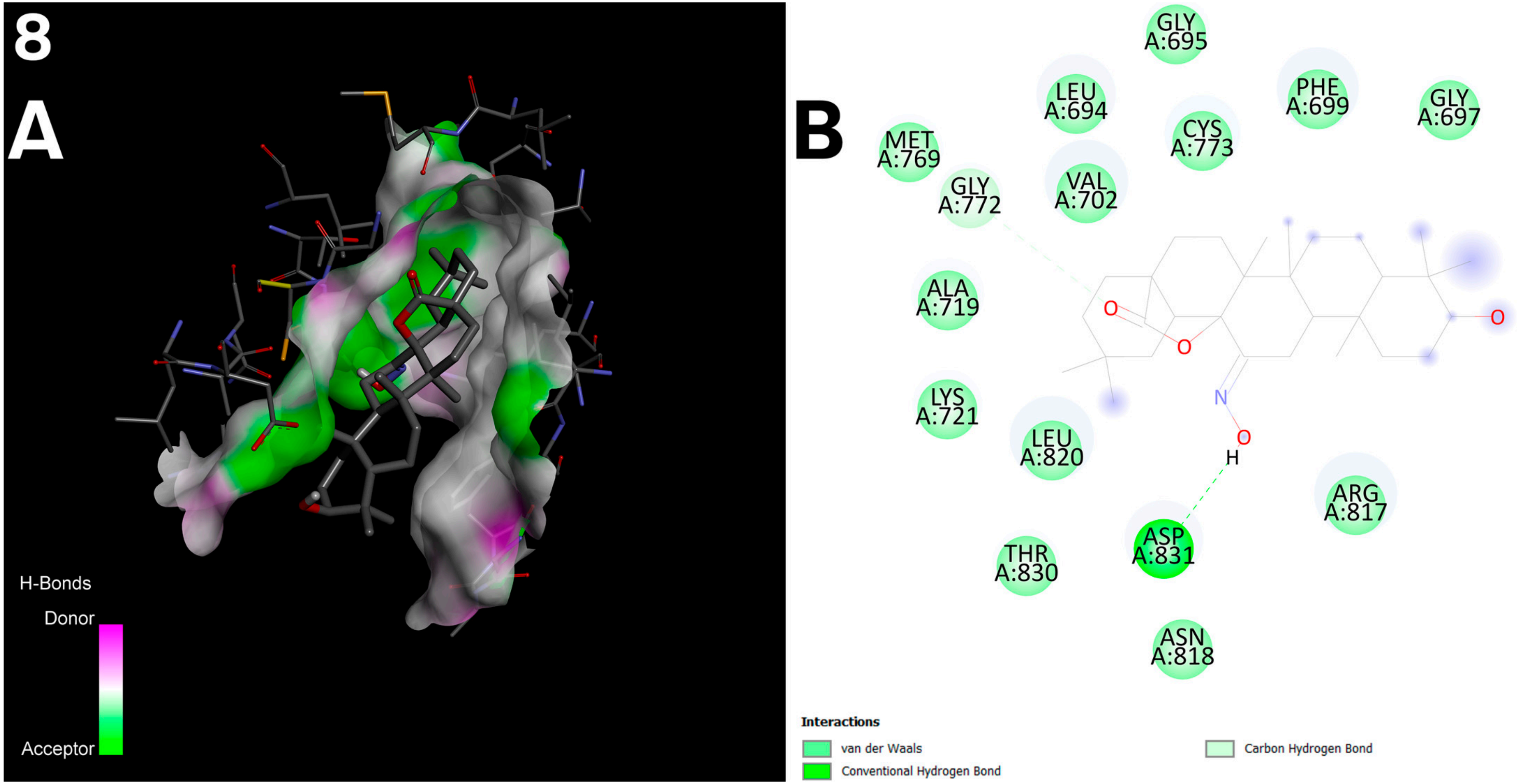

Figure S24. (A) Complex of compound 9 with 1M17 in C2 pocket. (B) Diagram in 2D with interactions.

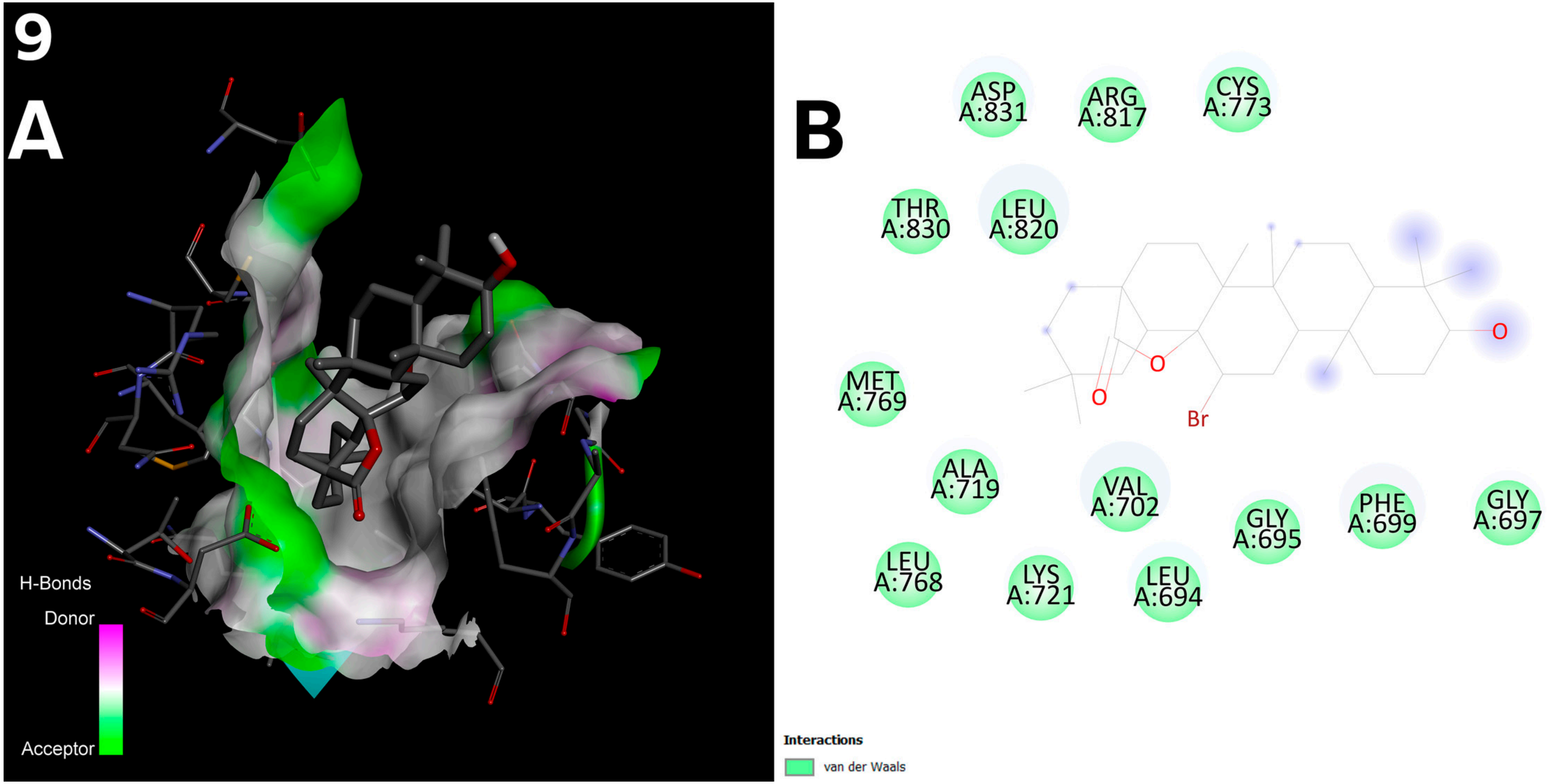

Figure S25. (A) Complex of compound 10 with 1M17 in C2 pocket. (B) Diagram in 2D with interactions.

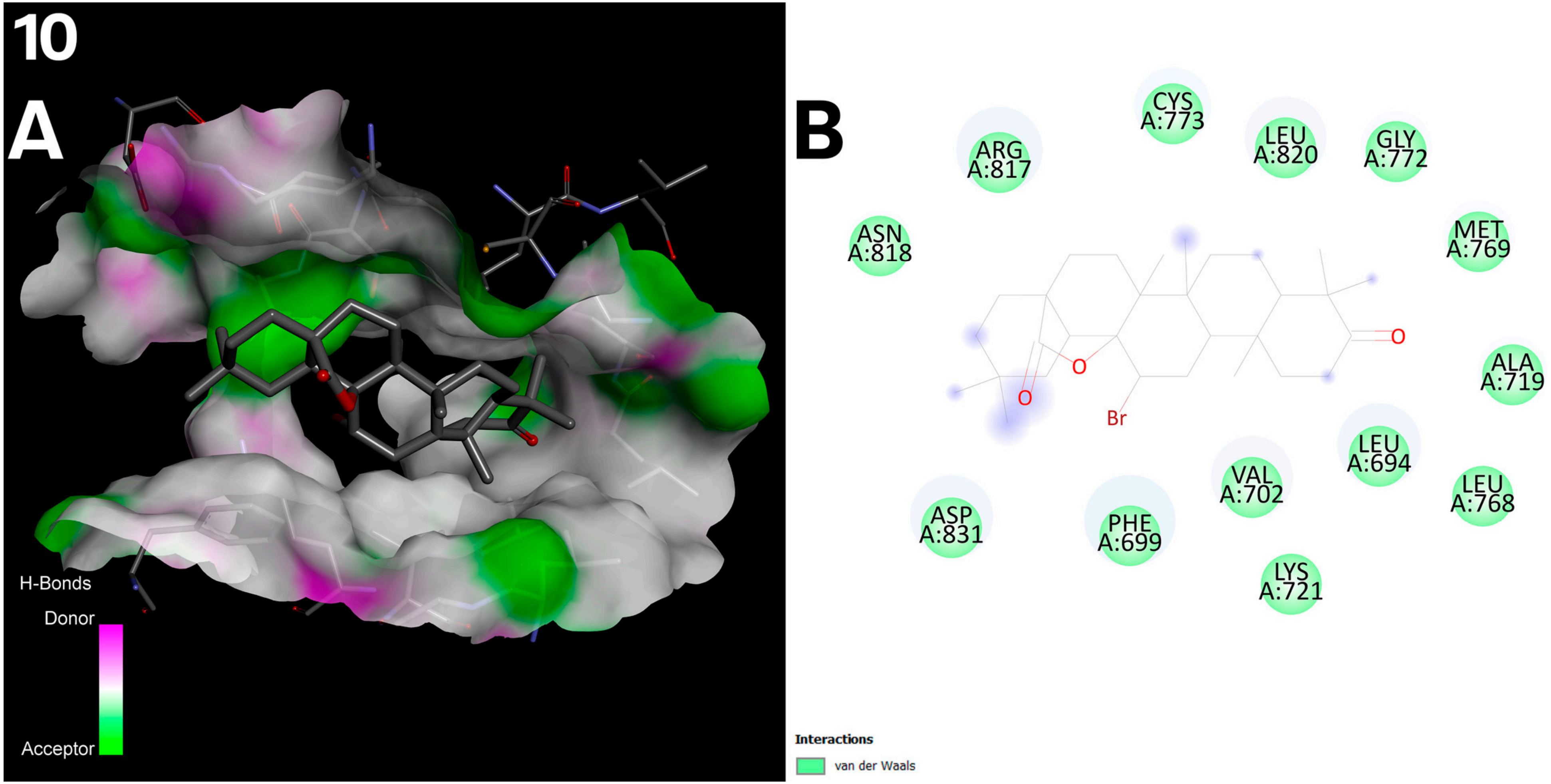

Figure S26. (A) Complex of compound 11 with 1M17 in C2 pocket. (B) Diagram in 2D with interactions.

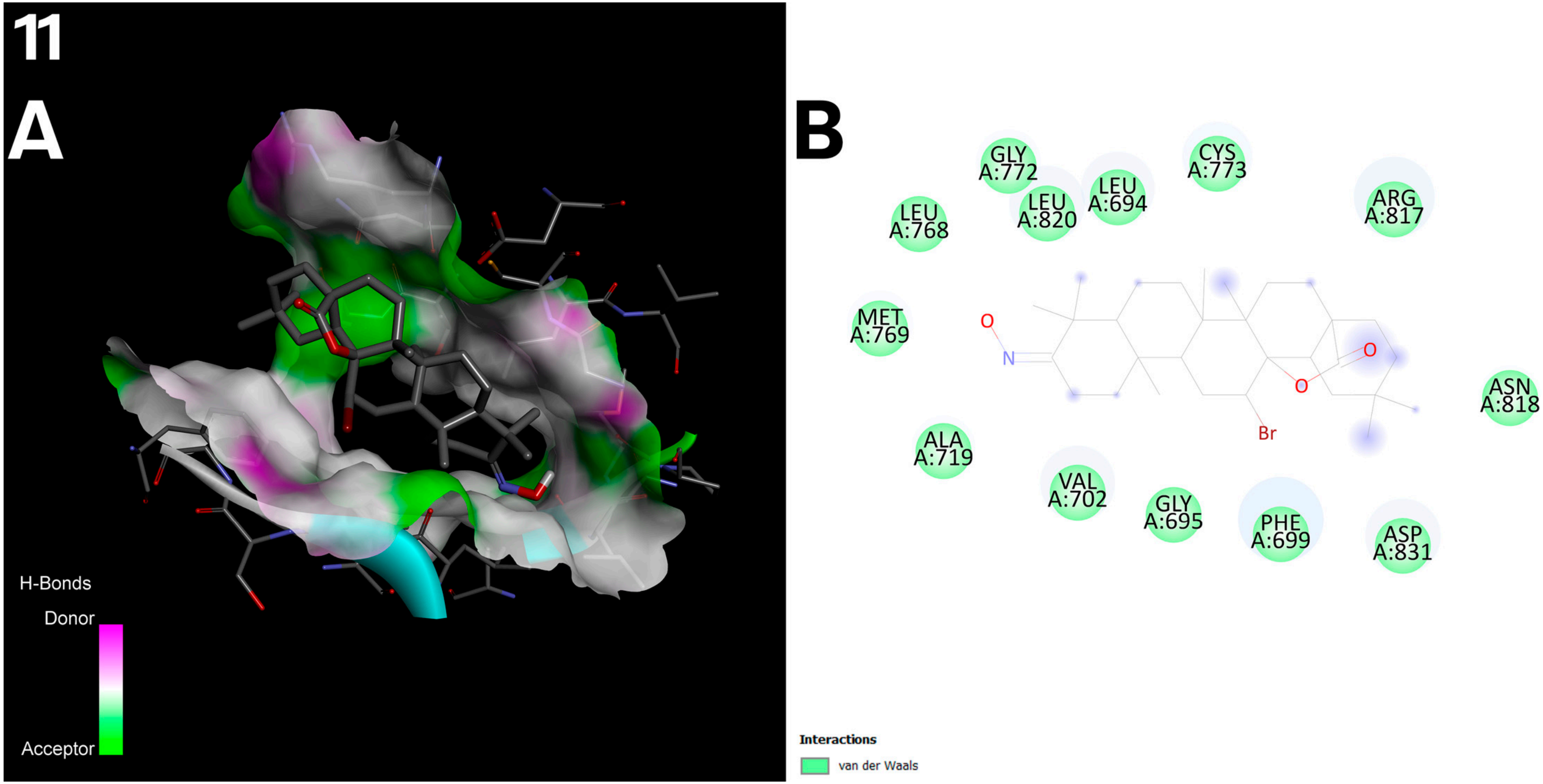

Figure S27. (A) Complex of compound 12 with 1M17 in C2 pocket. (B) Diagram in 2D with interactions.

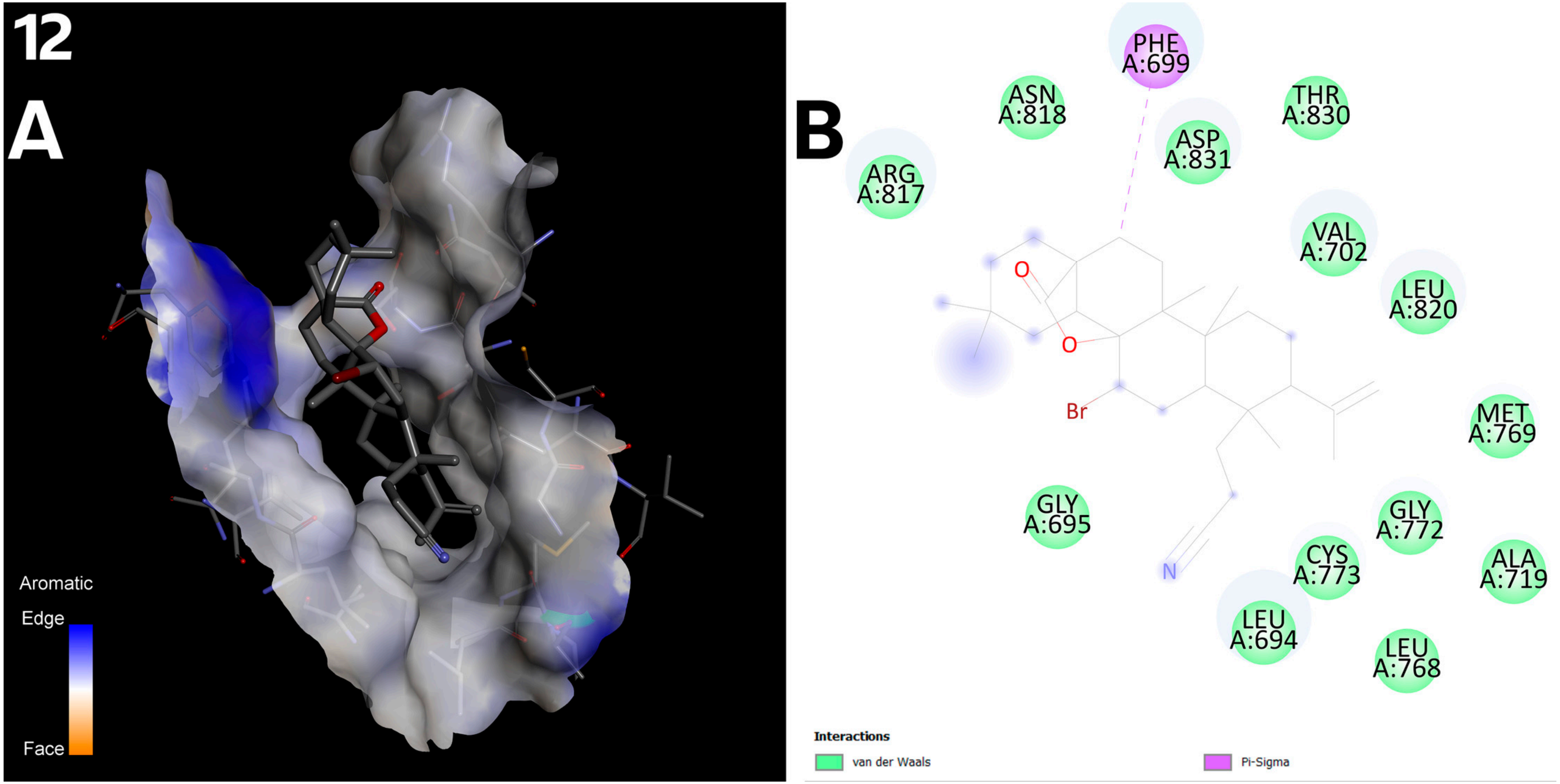

Figure S28. (A) Complex of compound 13 with 1M17 in C2 pocket. (B) Diagram in 2D with interactions.

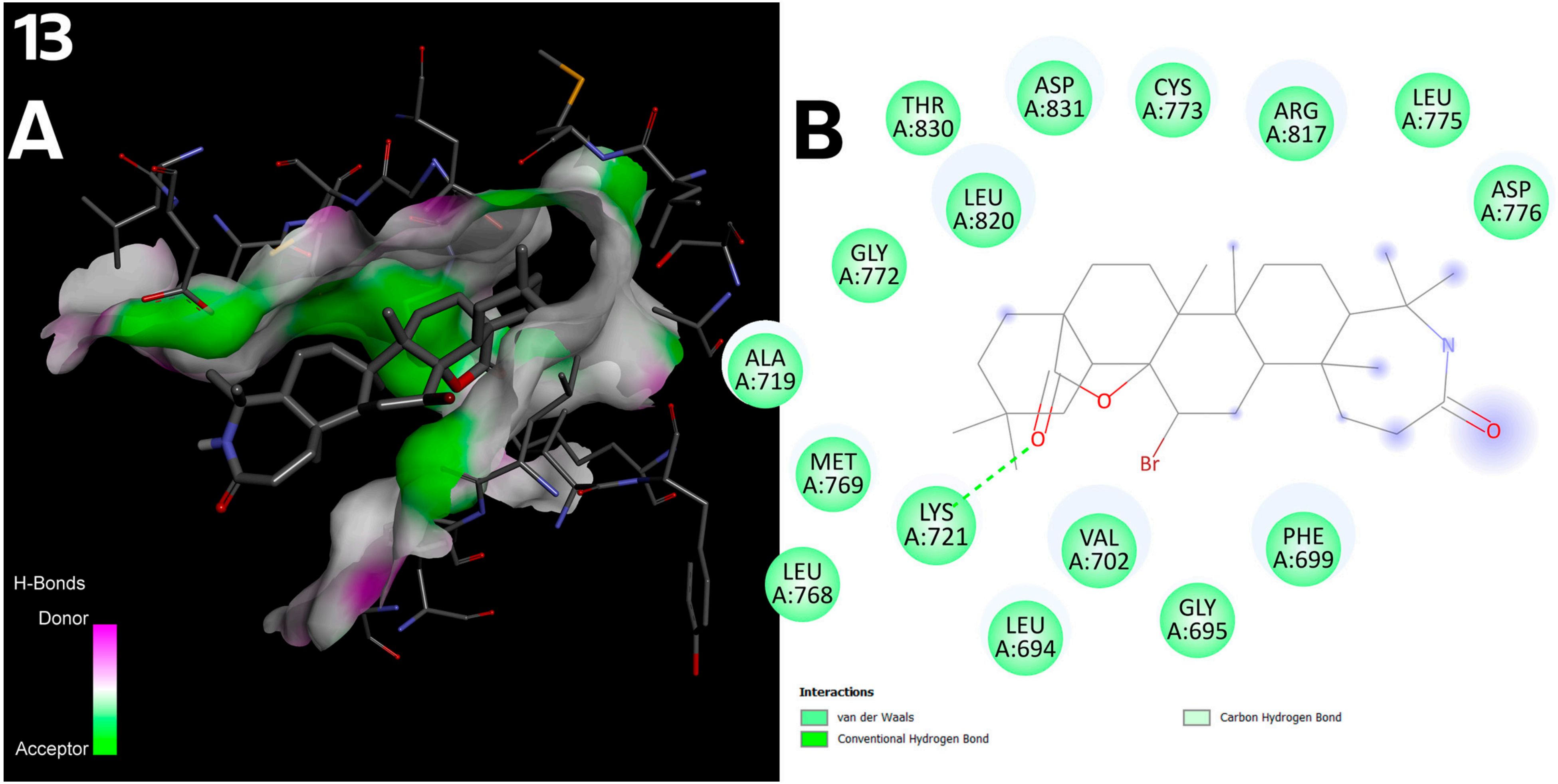

Figure S29. (A) Complex of compound 14 with 1M17 in C2 pocket. (B) Diagram in 2D with interactions.

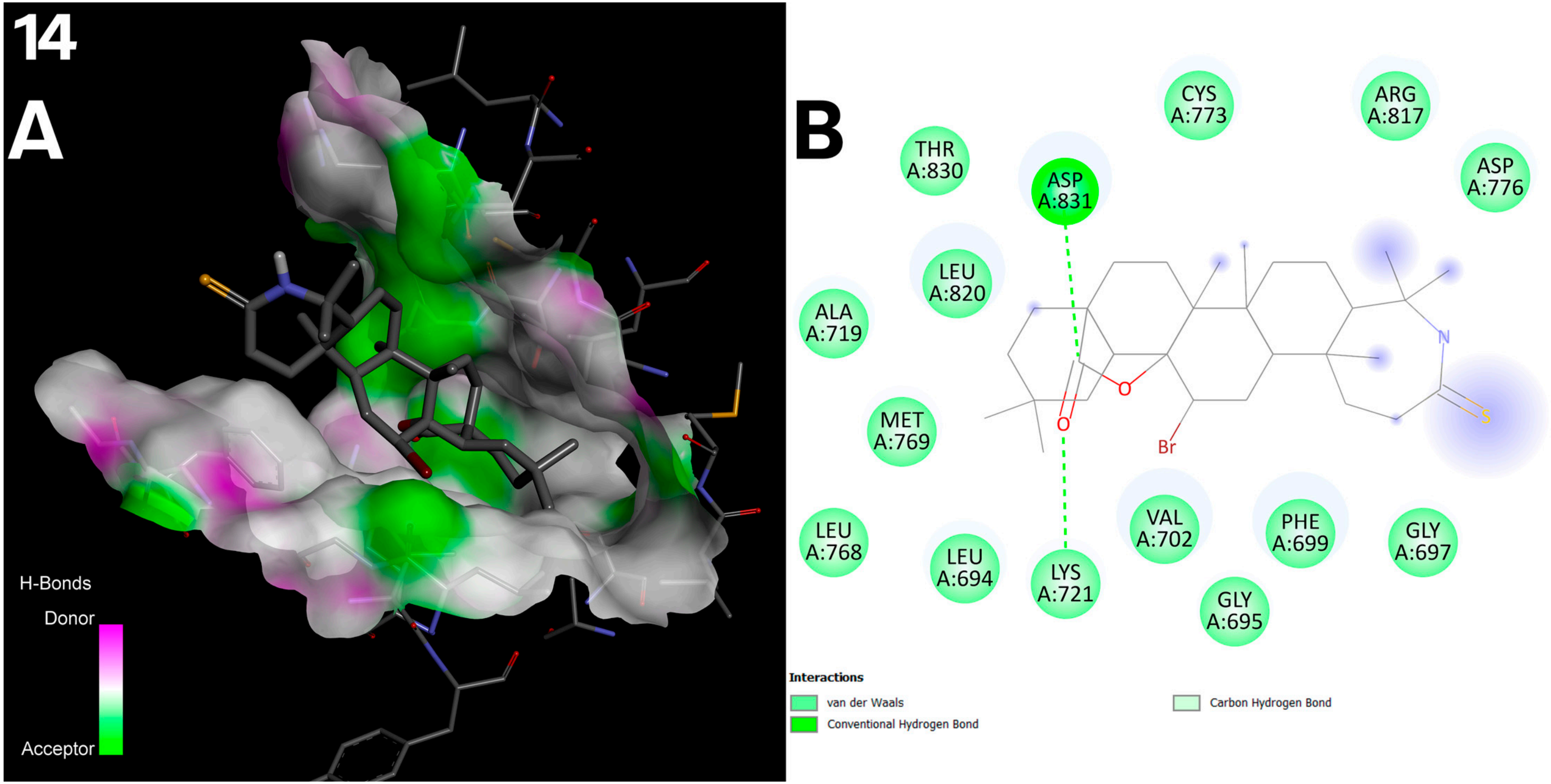

Table S3. Docking result for compounds **1 – 14** with EGFR tyrosine kinase domain molecule (PDB ID 1M17). Vina score in kcal·mol⁻¹.

Compound No	CurPocket ID	Vina score	Cavity volume (Å^3)	Center (x, y, z)	Docking size (x, y, z)
**1 OA**	C1	-8.0	991	36, 8, 51	23, 23, 23
	C2	-8.9	665	24, 0, 54	23, 23, 23
	C3	-6.2	416	31, -4, 40	23, 23, 23
	C4	-7.3	184	40, 18, 67	23, 23, 23
	C5	-7.0	173	3, 15, 63	23, 23, 23
**2**	C1	-8.0	991	36, 8, 51	24, 24, 24
	C2	-9.4	665	24, 0, 54	24, 24, 24
	C3	-6.2	416	31, -4, 40	24, 24, 24
	C4	-8.5	184	40, 18, 67	24, 24, 24
	C5	-6.8	173	3, 15, 63	24, 24, 24
**3**	C1	-7.8	991	36, 8, 51	22, 22, 22
	C2	-8.8	665	24, 0, 54	22, 22, 22
	C3	-6.3	416	31, -4, 40	22, 22, 22
	C4	-8.3	184	40, 18, 67	22, 22, 22
	C5	-7.2	173	3, 15, 63	22, 22, 22
**4**	C1	-8.0	991	36, 8, 51	24, 24, 24
	C2	-9.2	665	24, 0, 54	24, 24, 24
	C3	-6.5	416	31, -4, 40	24, 24, 24
	C4	-7.6	184	40, 18, 67	24, 24, 24
	C5	-7.1	173	3, 15, 63	24, 24, 24
**5**	C1	-8.5	991	36, 8, 51	22, 22, 22
	C2	-8.9	665	24, 0, 54	22, 22, 22
	C3	-7.0	416	31, -4, 40	22, 22, 22
	C4	-7.5	184	40, 18, 67	22, 22, 22

	C5	-8.0	173	3, 15, 63	22, 22, 22
	C1	-9.8	991	36, 8, 51	23, 23, 23
	C2	-10.3	665	24, 0, 54	23, 23, 23
**6**	C3	-6.5	416	31, -4, 40	23, 23, 23
	C4	-6.9	184	40, 18, 67	23, 23, 23
	C5	-7.2	173	3, 15, 63	23, 23, 23
	C1	-7.9	991	36, 8, 51	22, 22, 22
	C2	-9.1	665	24, 0, 54	22, 22, 22
**7**	C3	-7.3	416	31, -4, 40	22, 22, 22
	C4	-8.1	184	40, 18, 67	22, 22, 22
	C5	-7.6	173	3, 15, 63	22, 22, 22
	C1	-7.9	991	36, 8, 51	22, 22, 22
	C2	-9.2	665	24, 0, 54	22, 22, 22
**8**	C3	-7.0	416	31, -4, 40	22, 22, 22
	C4	-7.3	184	40, 18, 67	22, 22, 22
	C5	-7.1	173	3, 15, 63	22, 22, 22
	C1	-9.7	991	36, 8, 51	22, 22, 22
	C2	-8.7	665	24, 0, 54	22, 22, 22
**9**	C3	-6.1	416	31, -4, 40	22, 22, 22
	C4	-7.1	184	40, 18, 67	22, 22, 22
	C5	-7.9	173	3, 15, 63	22, 22, 22
	C1	-8.3	991	36, 8, 51	22, 22, 22
	C2	-9.7	665	24, 0, 54	22, 22, 22
**10**	C3	-5.9	416	31, -4, 40	22, 22, 22
	C4	-6.6	184	40, 18, 67	22, 22, 22
	C5	-7.2	173	3, 15, 63	22, 22, 22
	C1	-9.0	991	36, 8, 51	23, 23, 23
**11**	C2	-9.6	665	24, 0, 54	23, 23, 23
	C3	-6.1	416	31, -4, 40	23, 23, 23

	C4	-7.5	184	40, 18, 67	23, 23, 23
	C5	-7.2	173	3, 15, 63	23, 23, 23
	C1	-6.9	991	36, 8, 51	22, 22, 22
	C2	-8.1	665	24, 0, 54	22, 22, 22
**12**	C3	-6.1	416	31, -4, 40	22, 22, 22
	C4	-6.2	184	40, 18, 67	22, 22, 22
	C5	-6.9	173	3, 15, 63	22, 22, 22
	C1	-8.6	991	36, 8, 51	23, 23, 23
	C2	-9.8	665	24, 0, 54	23, 23, 23
**13**	C3	-6.5	416	31, -4, 40	23, 23, 23
	C4	-7.2	184	40, 18, 67	23, 23, 23
	C5	-8.3	173	3, 15, 63	23, 23, 23
	C1	-8.1	991	36, 8, 51	23, 23, 23
	C2	-9.9	665	24, 0, 54	23, 23, 23
**14**	C3	-6.3	416	31, -4, 40	23, 23, 23
	C4	-6.8	184	40, 18, 67	23, 23, 23
	C5	-7.4	173	3, 15, 63	23, 23, 23
